# MOF-Triggered Synthesis of Subnanometer Ag^0^_2_ Clusters and Fe^3+^ Single Atoms: Heterogenization Led to Efficient and Synergetic One-Pot Catalytic Reactions

**DOI:** 10.1021/jacs.3c02155

**Published:** 2023-04-28

**Authors:** Estefanía Tiburcio, Yongkun Zheng, Cristina Bilanin, Juan Carlos Hernández-Garrido, Alejandro Vidal-Moya, Judit Oliver-Meseguer, Nuria Martín, Marta Mon, Jesús Ferrando-Soria, Donatella Armentano, Antonio Leyva-Pérez, Emilio Pardo

**Affiliations:** † Instituto de Ciencia Molecular (ICMol), Universidad de Valencia, 46980 Paterna, Valencia, Spain; ‡ Instituto de Tecnología Química (UPV-CSIC), Universitat Politècnica de València-Consejo Superior de Investigaciones Científicas, Avda. de los Naranjos s/n, 46022 Valencia, Spain; § Departamento de Ciencia de los Materiales e Ingeniería Metalúrgica y Química Inorgánica, Facultad de Ciencias, Universidad de Cádiz, Campus Universitario Puerto Real, 11510 Puerto Real, Cádiz, Spain; ∥ Dipartimento di Chimica e Tecnologie Chimiche (CTC), Università della Calabria, 87036 Rende, Cosenza, Italy

## Abstract

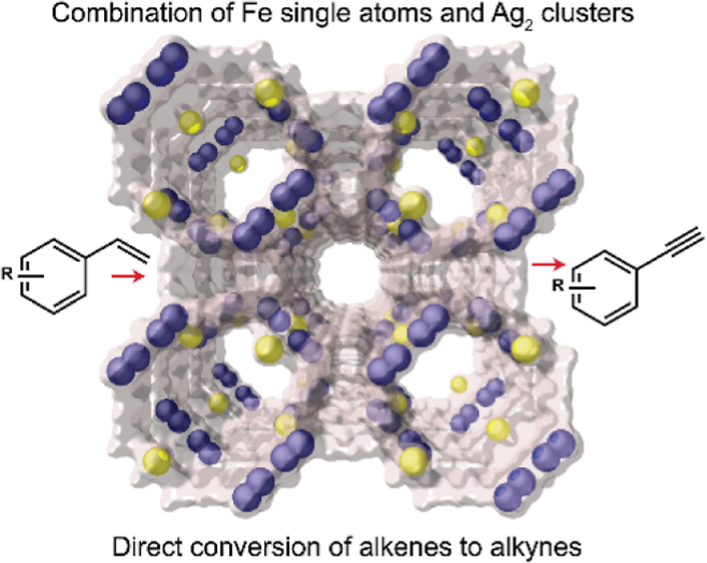

The combination of well-defined Fe^3+^ isolated single-metal atoms and Ag_2_ subnanometer metal clusters within the channels of a metal–organic framework (MOF) is reported and characterized by single-crystal X-ray diffraction for the first time. The resulting hybrid material, with the formula [Ag^0^_2_(Ag^0^)_1.34_Fe^III^_0.66_]@Na^I^_2_{Ni^II^_4_[Cu^II^_2_(Me_3_mpba)_2_]_3_}·63H_2_O (**Fe**^**3+**^**Ag**^**0**^_**2**_**@MOF**), is capable of catalyzing the unprecedented direct conversion of styrene to phenylacetylene in one pot. In particular, **Fe**^**3+**^**Ag**^**0**^_**2**_**@MOF**—which can easily be obtained in a gram scale—exhibits superior catalytic activity for the TEMPO-free oxidative cross-coupling of styrenes with phenyl sulfone to give vinyl sulfones in yields up to >99%, which are ultimately transformed, in situ, to the corresponding phenylacetylene product. The results presented here constitute a paradigmatic example of how the synthesis of different metal species in well-defined solid catalysts, combined with speciation of the true metal catalyst of an organic reaction in solution, allows the design of a new challenging reaction.

## Introduction

Isolated single-metal atoms (SMAs) and subnanometer metal clusters (SNMCs)^1−7^ have been revealed as extraordinary materials with exciting physicochemical properties due to their unique intrinsic chemical nature and electronic features.^8−11^ SMAs and SNMCs are hailed to trigger a paradigmatic shift in diverse technological applications.^4,9^ However, prior to that, certain issues related to the total control of their synthesis, especially the scale-up, as well as stabilization and atomically precise characterization, need to be solved.^4,12−14^ During the last decade, intense research has been performed in order to gain control over the synthesis of both SNMCs and SMAs and, indeed, commendable advances have been obtained.^15−20^ Despite such advances, challenging issues remain to be further studied, and others have not even been explored, which inherently represent new avenues of research full of possibilities waiting to be explored. This is the case, for example, of the preparation and stabilization of different metallic species, with distinct chemical nature and atomicity—that is, SMAs and SNMCs—coexisting and having the potential to be ready to be used concomitantly.^20^

Having in mind the literature precedents on the synthesis of independent SMAs and SNMCs,^1−7^ a priori, porous solids seem to be the most suitable materials to tackle this challenge. Among them, metal–organic frameworks (MOFs)^21−24^ stand out due to their inherent unique features, such as rich host–guest chemistry and high crystallinity.^25−29^ These have enabled the preparation/incorporation and stabilization of intrinsically unstable SMAs and SNMCs within MOFs channels^30,31^ (SMAs@MOFs and SNMCs@MOFs), leading to very performant catalytic materials that outperform in some cases the state-of-the-art catalysts.^32^ These materials are fascinating from a chemical and crystallographic point of view since single-crystal X-ray diffraction (SCXRD) allows obtaining unique snapshots of their chemical nature and atomicity, as well as the stabilizing interactions established between SMAs and SNMCs and the MOFs pore.^32−35^ Thus, it seems reasonable and feasible to envision moving forward the frontiers of knowledge and integrating SMAs and SNMCs of different metals within the same MOF channels (SMAs–SNMCs@MOFs), which represent an unprecedented level of complexity, both structurally and chemically, and, in turn, offer the possibility to study novel catalytic transformations that require or benefit from the participation of distinct metallic catalysts.

The direct catalytic conversion of alkenes to alkynes is an unmet synthetic pathway with high potential impact in organic synthesis.^36^ Alkynes are pluripotential building blocks for organic synthesis, which engage in a multitude of reactions such as hydrogenation, hydroaddition, cross-coupling, metathesis, and pericyclic reactions, to name a few. To give access to that are stereo-defined alkenes, ketones, other alkynes, and cyclic compounds.^37−40^ However, alkynes are generally expensive and difficult to implement in scaling-up (kilogram) studies since their synthesis often relies on highly energetic intermediates, such as, for instance, vicinal per-halides, selenides, diazo, or phosphonate compounds.^39,41−45^ The only bulk alkyne product in industry is acetylene, and, in much less scale, phenylacetylene. Thus, it is not surprising that commercial alkynes with relatively affordable prices are only those coming from acetylene as a starting material, such as propargyl alcohols (acetylides + carbonyl compounds).

**Figure 1:**
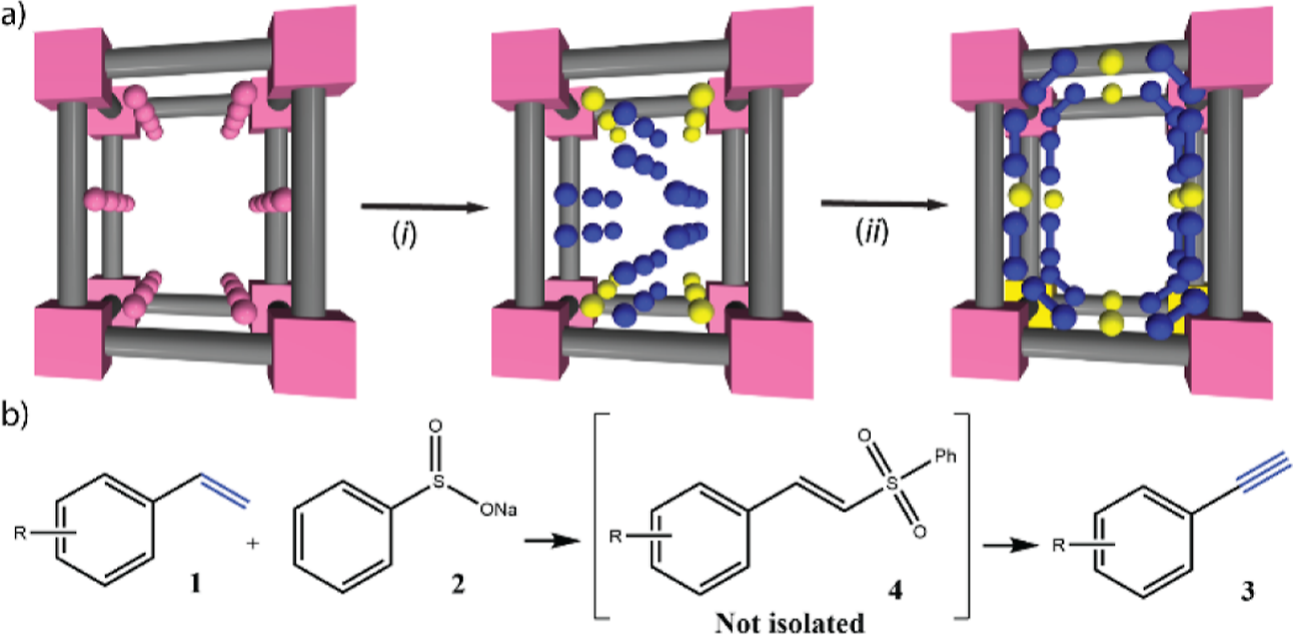
(a) Schematic representation of the three consecutive PS steps followed for the synthesis of **Fe**^**3+**^**Ag**^**0**^_**2**_**@MOF**. Two successive metal-exchange processes leading to the replacement of Ni^II^ cations residing at the pores by Ag^I^ and Fe^III^ ions (i), followed by the immediate reduction of silver cations (ii) to form the SNMCs Ag^0^_2_ in **Fe**^**3+**^**Ag**^**0**^_**2**_**@MOF**. (b) Proposed approach for the one-pot conversion of alkenes into alkynes, based on the metal-catalyzed oxidative cross-coupling of styrenes **1** with phenyl sulfone **2** to give phenylacetylenes **3** after the elimination of the sulfone moiety of intermediates vinyl sulfones **4**.

Aiming to find solutions to both scientific challenges, first, we have taken advantage of solid-state post-synthetic (PS) methodologies^46^ on a water-stable and a robust three-dimensional MOF of formula, Ni_2_^II^{Ni_4_^II^[Cu_2_^II^(Me_3_mpba)_2_]_3_}·54H_2_O (**Ni**_**2**_**@MOF**),^47^ to synthesize, in three consecutive PS steps, a novel MOF with formula [Ag^0^_2_(Ag^0^)_1.34_Fe^III^_0.66_]@Na^I^_2_{Ni^II^_4_[Cu^II^_2_(Me_3_mpba)_2_]_3_}·63H_2_O (**Fe**^**3+**^**Ag**^**0**^_**2**_**@MOF**), which was obtained after two consecutive cation exchanges of Ni^2+^ cations by Ag^+^ and Fe^3+^ ones and concomitant reduction with NaBH_4_ to obtain the final hybrid material **Fe**^**3+**^**Ag**^**0**^_**2**_**@MOF**. This hybrid MOF integrates Fe^3+^-SMAs and both Ag^0^_2_-SNMCs and Ag^0^-SMAs within their channels (Figure 1a). Interestingly, the crystal structure of the final **Fe**^**3+**^**Ag**^**0**^_**2**_**@MOF** could be precisely characterized by means of SCXRD. A close structural analysis of SMAs, SNMCs, and the MOF hosting network in **Fe**^**3+**^**Ag**^**0**^_**2**_**@MOF**, in comparison with two previously reported MOFs containing, separately, Fe^3+^-SMAs^48^ and SNMCs of Ag^0^_2_-dimers^49^ confined within their channels, [Fe^III^(H_2_O)_6_][Fe_2_^III^(μ-O)_2_(H_2_O)_6_]_1/2_{Ni^II^_4_[Cu^II^_2_(Me_3_mpba)_2_]_3_}·72H_2_O (**Fe**^**3+**^**@MOF**) and [Ag^0^_2_]@Ag^I^_2_Na^I^_2_{Ni^II^_4_[Cu^II^_2_(Me_3_mpba)_2_]_3_}·48H_2_O (**Ag**^**0**^_**2**_**@MOF**), respectively, revealed that the metallic entities do not suffer significant changes in terms of their atomicity, oxidation state, chemical surroundings, and relative position in the channels, and there is no structural distortion of the MOF network. Thus, these systems represent an exceptional opportunity to study in a neat manner, without structural/electronic ambiguities, the catalytic role and activity of each metallic entity for a given reaction. To this end, we have evaluated the potential of **Fe**^**3+**^**Ag**^**0**^_**2**_**@MOF** in the challenging conversion of styrenes to phenylacetylenes in one pot. We based our approach on the metal-catalyzed oxidative cross-coupling of styrenes **1** with phenyl sulfone **2** to give phenylacetylenes **3**, after elimination of the sulfone moiety in intermediates **4** (Figure 1b)—an otherwise incompatible procedure in the homogenous phase with previously reported methods.^50−54^ We have found that **Fe**^**3+**^**Ag**^**0**^_**2**_**@MOF** outperforms all the tested catalysts, efficiently catalyzing this conversion for phenylacetylene in one pot (99%), without the addition of external organic oxidants, and with a good reusability (up to 5 cycles). Noteworthily, this contrasts with the low catalytic activity not only of the combination of both silver and iron metals in homogenous solution but also when just one metal is supported and the other remains in solution, or even when using physically mixed mixtures of **Fe**^**3+**^**@MOF** and **Ag**^**0**^_**2**_**@MOF**. Thus, the heterogenization within MOF channels, integrating SMAs and SNMCs of different metal ions, represents an exceptional example of how structural complexity/diversity could be transformed into evolved catalytic functionality.

## Results and Discussion

Herein, we present a synthetic pathway to obtain metallic species of distinct chemical nature and atomicity/nuclearity supported and stabilized through interactions within MOFs pores. In particular, **Fe**^**3+**^**Ag**^**0**^_**2**_**@MOF**, containing Fe^3+^-SMAs and Ag^0^_2_-SNMCs within MOF’s large octagonal channels, was obtained in a sequential single-crystal to single-crystal manner after three PS steps, two successive metal-exchange processes on Ni_2_^II^{Ni_4_^II^[Cu_2_^II^(Me_3_mpba)_2_]_3_}·54H_2_O (**Ni**_**2**_**@MOF**), where Ni^II^ cations residing in the pores were exchanged by silver(I) and iron(III), and a concomitant reduction of silver atoms. Alternatively, **Fe**^**3+**^**Ag**^**0**^_**2**_**@MOF** could be obtained on a multi-gram scale following a similar procedure using polycrystalline powder (see the Experimental Section). Remarkably, we have obtained defined and ordered metallic entities of different metal ions with distinct numbers of atoms constituting each entity within the same MOF, which contrasts with previously reported methods in porous materials, where more commonly mixtures of different metallic species with random distribution are obtained.^55^ In addition, we would like to stress that supporting well-defined SNMC entities of silver on solids is already a very challenging task.^49,56−59^ Silver atoms present a strong tendency to be reduced and form nanoparticles even under the simple action of light and also possess a very reactive nature toward other metals, leading to redox reaction and alloys in a wide variety of reaction conditions. Thus, the development of well-defined SNMCs Ag^0^_2_ coexisting with other metal ions, in our case Fe^3+^-SMAs, within the same MOF is extremely valuable on its own, beyond the targeted catalytic application (see below). The nature of such a complex hybrid assembly was established by a combination of physical characterization techniques: inductively coupled plasma mass spectrometry (ICP–MS, Table S1), CHN and thermogravimetric analysis (TGA), powder X-ray diffraction (PXRD) analysis, scanning electron microscopy–energy dispersive X-ray spectroscopy (SEM–EDX, Table S1), N_2_ adsorption isotherm, Fourier transform infrared (FTIR) spectroscopy, and X-ray photoelectron spectroscopy (XPS). Finally, the real crystal structure of **Fe**^**3+**^**Ag**^**0**^_**2**_**@MOF** was unveiled by SCXRD. This was possible thanks to the structural robustness and nice crystallinity of the MOF selected as the host to perform the PS processes, as well as the application of cutting-edge crystallography.

### Structure of **Fe**^**3+**^**Ag**^**0**^_**2**_**@MOF**

**Figure 2:**
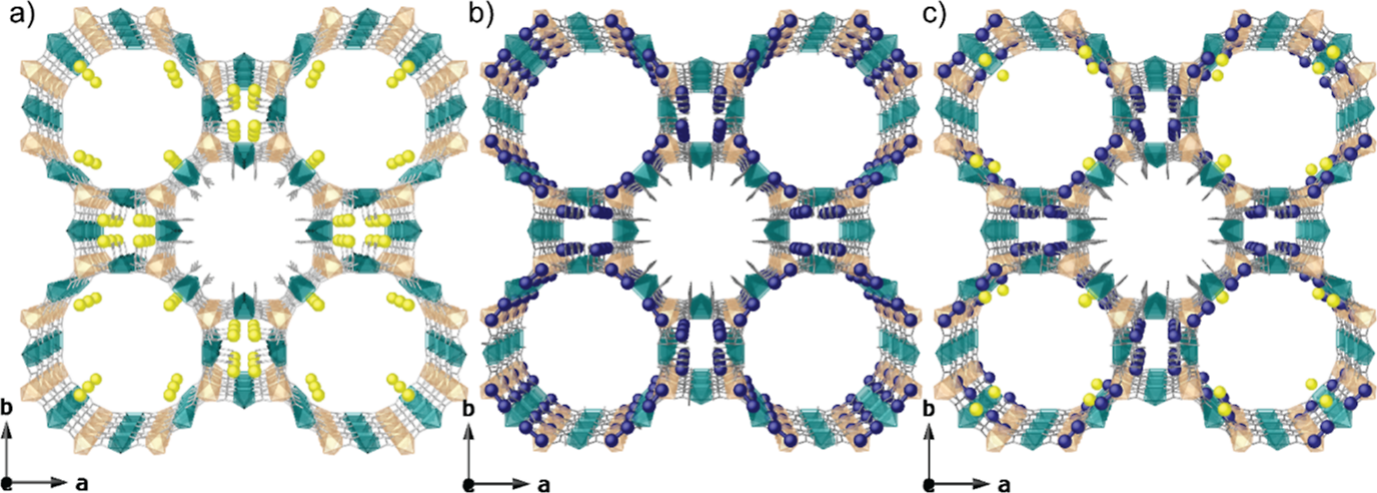
Crystal structures of **Fe**^**3+**^**@MOF**(48) (a), **Ag**^**0**^_**2**_**@MOF**(49) (b), and **Fe**^**3+**^**Ag**^**0**^_**2**_**@MOF** (c). Copper and nickel atoms from the network are represented by cyan and orange polyhedra, respectively, whereas organic ligands are depicted as grey sticks. Yellow and dark blue spheres represent Fe and Ag atoms, respectively.

The SCXRD data of **Fe**^**3+**^**Ag**^**0**^_**2**_**@MOF** evidenced an anionic Ni^II^_4_Cu^II^_6_ open-framework structure isoreticular to its ancestor, which crystallizes in the *P*4/*mmm* space group of the tetragonal system. Although an important statistical and dynamic disorder affect the visualization of metal positions in large pores (Crystallographic section in Supporting Information), crystallography unambiguously unveils the formation of Ag^0^_2_ dimers, stabilized by the walls of the hydrophilic octagonal channels [virtual diameter of 2.2 nm], (crystallographically) mixed with Fe^3+^ cations residing as well in the larger accessible hydrophilic octagonal channels, in proximity to the preferential cationic sites already unveiled in **Fe**^**3+**^**@MOF** (Figure 2). These results are strongly supported by SEM–EDX, ICP–MS, and XPS analyses (see below).

**Figure 3:**
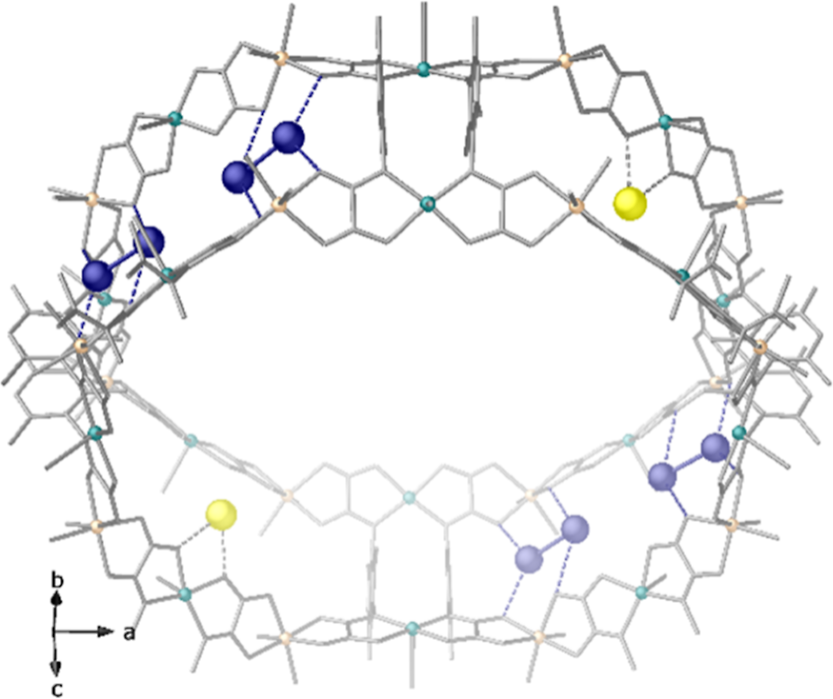
Details of **Fe**^**3+**^**Ag**^**0**^_**2**_**@MOF** X-ray structure showing supramolecular interactions stabilizing Ag^0^_2_ dimers and Fe^3+^ ions in hydrophilic octagonal channels (intradimer Ag···Ag distance of 2.67(3) Å). The copper and nickel atoms in the network are represented by cyan and orange spheres, respectively, whereas organic ligands are depicted as grey sticks. Yellow and dark blue spheres represent Fe and Ag atoms, respectively.

Both Ag^0^_2_ dimers and Fe^3+^ cations, decorating pore walls of the large octagonal channels, are mixed with a population of 33 and 67%, respectively, and stabilized by non-covalent interactions involving oxamate oxygen atoms. In fact, such disorder gives a mixed view of **Fe**^**3+**^**Ag**^**0**^_**2**_**@MOF**, understandable considering that a crystal structure is the spatial average of all chemical fragments in the crystal via only one unit cell. Figure 3 displays Ag^0^_2_ dimers [intradimer Ag···Ag distance of 2.67(3) Å] trapped and stabilized nearby the walls of the largest pores of the network by Ag···O_oxamate_ interactions [Ag^0^_2_···O_oxamate_ of 2.87(2) and 3.09(3) Å]. Further Ag^0^_2_ dimers [intradimer Ag···Ag distance of 2.93(3) Å] are formed and blocked in the small and thus less accessible square pores of the porous network, stabilized by Ag^0^···O_oxamate_ interactions at a distance of 2.83(2) Å] (Figure S1). The Fe^3+^ surrounding in the **Fe**^**3+**^**Ag**^**0**^_**2**_**@MOF** crystal structure confirms that these metal ions are retained via Fe^3+^···O_oxamate_ contacts [Fe^3+^···O_oxamate_ of 2.53(2) Å] (Figures S1–S3). Further hydrated charge-counterbalancing alkali Na^+^ cations are retained, as constantly found in this hosting matrix, in the preferential cationic sites, contributing to the outstanding robustness of the final material (Figures S4 and S5). Interestingly, when comparing the crystal structure of **Fe**^**3+**^**Ag**^**0**^_**2**_**@MOF** with the ones of the previously reported materials **Fe**^**3+**^**@MOF** and **Ag**^**0**^_**2**_**@MOF** (Table S2), certain similarities can be observed together with important differences. In **Fe**^**3+**^**@MOF**, the Fe^3+^···MOF contacts, stabilizing the metal ions, were water-mediated.^48^ Indeed, the [Fe^III^(H_2_O)_6_]^3+^ monomers were well-equipped to establish strong hydrogen bonds with the oxygen atoms of the pore walls and water molecules surrounding Fe^III^ ions. In the **Fe**^**3+**^**Ag**^**0**^_**2**_**@MOF** crystal structure, Fe^3+^ ions are located in the same preferential cationic sites but exhibit the shortest Fe^3+^···O_oxamate_ separation, suggesting a direct contact with the walls of the network (likely at the origin of the highly disordered and not detected by Δ*F* maps water molecules completing the Fe^3+^ sphere of coordination).

On the other hand, from the comparison between **Fe**^**3+**^**Ag**^**0**^_**2**_**@MOF** and **Ag**^**0**^_**2**_**@MOF**, it is worth underlining the strict similarity, even in terms of structural parameters (i.e. intradimer Ag···Ag distances), which confirms the intrinsic capability of the anionic Ni^II^_4_Cu^II^_6_ open-framework to synthesize and stabilize the really challenging Ag^0^_2_ dimers. So, apart from the structural richness gained with **Fe**^**3+**^**Ag**^**0**^_**2**_**@MOF**, these systems are excellent test benches to study, without structural/electronic ambiguities, the activity of each metallic entity, either isolated or integrated in the same MOF, for a given catalytic reaction. Offering a singular opportunity to investigate the potential cooperative/symbiotic effect of having simultaneously two different and well-defined metallic species at a molecular distance in a solid support (see below).

The experimental PXRD pattern of **Fe**^**3+**^**Ag**^**0**^_**2**_**@MOF**, as well as those of previously reported **Fe**^**3+**^**@MOF** and **Ag**^**0**^_**2**_**@MOF** are identical to the calculated ones (Figure S6), which confirms both the homogeneity of the samples and the isostructurality of the single-crystals selected for SCXRD. A mapping analysis with SEM–EDX of **Fe**^**3+**^**Ag**^**0**^_**2**_**@MOF** showed a homogenous distribution of iron and silver metal ions at the analyzed crystals (Figure S7), which evidences the close proximity of distinct metallic entities within MOF channels observed with SCXRD, ruling out a segregation of SMAs and SNMCs and the formation of particles of higher size. In order to establish the solvent content of **Fe**^**3+**^**Ag**^**0**^_**2**_**@MOF**, TGA analysis was performed under a dry N_2_ atmosphere (Figure S8). The observed weight loss corresponds to 63 water molecules, which agree with the CHN analysis (see Supporting Information). XPS of **Fe**^**3+**^**Ag**^**0**^_**2**_**@MOF** (Figure S9) is consistent with those previously reported for **Fe**^**3+**^**@MOF**(48) and **Ag**^**0**^_**2**_**@MOF**(49) and suggests the presence of single-site Fe^3+^-SMAs and both Ag^0^_2_-SNMCs and Ag^0^-SMAs within the same MOF. Thus, we carried out XPS measurements for this heterometallic material before (Figure S9a) and after reduction with NaBH_4_ (Figure S9b), which confirmed that only Ag^+^ cations are effectively reduced by NaBH_4_. Before reduction, Ag 3d_5/2_ and Ag 3d_3/2_ bands are observed at 367.7 and 373.7 eV, which are indicative of Ag^+^^49^ (Figure S9a left). After reduction, peaks at 368.9 and 374.7 eV can be observed (Figure S9b left), which are attributed to reduced Ag^0^ atoms and confirm the full reduction of Ag^+^ atoms after reacting with NaBH_4_. This point contrasts with that observed for the previously reported homometallic **Ag**^**0**^_**2**_**@MOF**,^49^ where only 50% of Ag^+^ cations (those located in larger channels) were reduced by NaBH_4_ forming Ag^0^_2_ nanoclusters, which could be explained by the higher content of Ag^+^ present in the previously reported MOF. On the other hand, the analysis of the iron bands before and after introduction of NaBH_4_ allows confirming that Fe^3+^ cations neither are reduced by NaBH_4_ nor reoxidized upon exposure to air. Indeed, the fitting of the Fe 2p_3/2_ and Fe 2p_1/2_ shows well-known peaks for Fe(III).^60^ In particular, peaks at 711.2/711.1 and 713.8/713.9 eV, before and after reduction, respectively, can be clearly associated with Fe^3+^ 2p_3/2_, while peaks at 724.9/724.2 and 726.9/726.7 eV are associated with Fe^3+^ 2p_1/2_. Additionally, other satellite peaks, typical of Fe^3+^ ions, can be also observed (Figure S9). The N_2_ adsorption isotherms at 77 K for **Fe**^**3+**^**@MOF**, **Ag**^**0**^_**2**_**@MOF**, and **Fe**^**3+**^**Ag**^**0**^_**2**_**@MOF** are shown in Figure S10. The calculated Brunauer–Emmett–Teller surface areas^61,62^ are 1013, 1183, and 1197 m^2^/g for **Fe**^**3+**^**@MOF**, **Ag**^**0**^_**2**_**@MOF**, and **Fe**^**3+**^**Ag**^**0**^_**2**_**@MOF**, respectively, with calculated pore sizes^63^ of 1.11, 1.28, and 1.31 nm. Thus, despite the increase in complexity within the MOFs channels, the hybrid material maintains the required porosity to perform catalytic events within their channels.

The formation of Ag^0^_2_ clusters in **Fe**^**3+**^**Ag**^**0**^_**2**_**@MOF** is also supported by the observation in UV–vis emission spectrophotometry measurements of the expected fluorescence bands for Ag_2_, which according to the jellium model should appear at ∼300 nm after irradiation at ∼250 nm (Figure S11). These emission bands were not observed in the corresponding **Fe**^**3+**^**@MOF**. However, in order to further confirm the presence of the Ag_2_ clusters, aberration-corrected high-angle annular dark-field scanning transmission electron microscopy (AC-HAADF-STEM) measurements of **Fe**^**3+**^**Ag**^**0**^_**2**_**@MOF** were carried out in combination with EDX analyses. The results (Figure S12) show the homogeneous distribution of all metal atoms of Cu, Ni, Fe, and Ag in the MOF structure. A denoised and background removed analysis of an AC-HAADF-STEM image (Figure S13) confirms the presence of the Ag atoms in sub-nanometer clusters, and the corresponding histogram shows that the average diameter of these Ag clusters is ∼0.3 nm, which corresponds to Ag_2_ species. The analysis of two individual Ag_2_ clusters (Figure 5) shows that the bond distances between atoms fit well with Ag_2_ species. These results strongly support the formation of the Ag^0^_2_ species in **Fe**^**3+**^**Ag**^**0**^_**2**_**@MOF**. Notice that individual Fe atoms cannot be visualized by HAADF-STEM due to the presence of Cu and Ni in the MOF.

**Figure 4:**
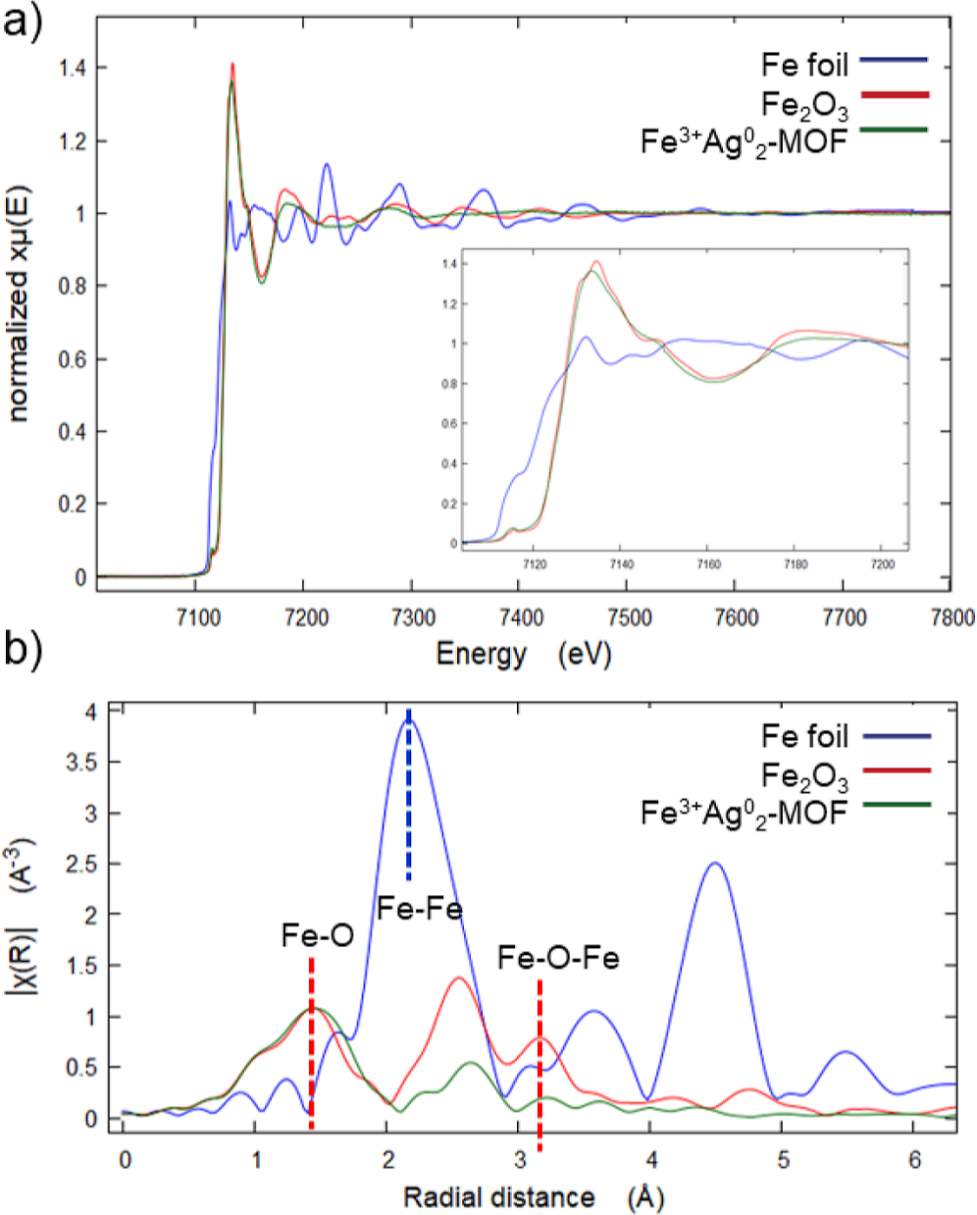
Fe K-edge XANES (top) and EXAFS (bottom) spectra of the **Fe**^**3+**^**Ag**^**0**^_**2**_**@MOF** (green lines), compared to Fe foil (blue lines) and Fe_2_O_3_ (red lines) as standard samples.

**Figure 5:**
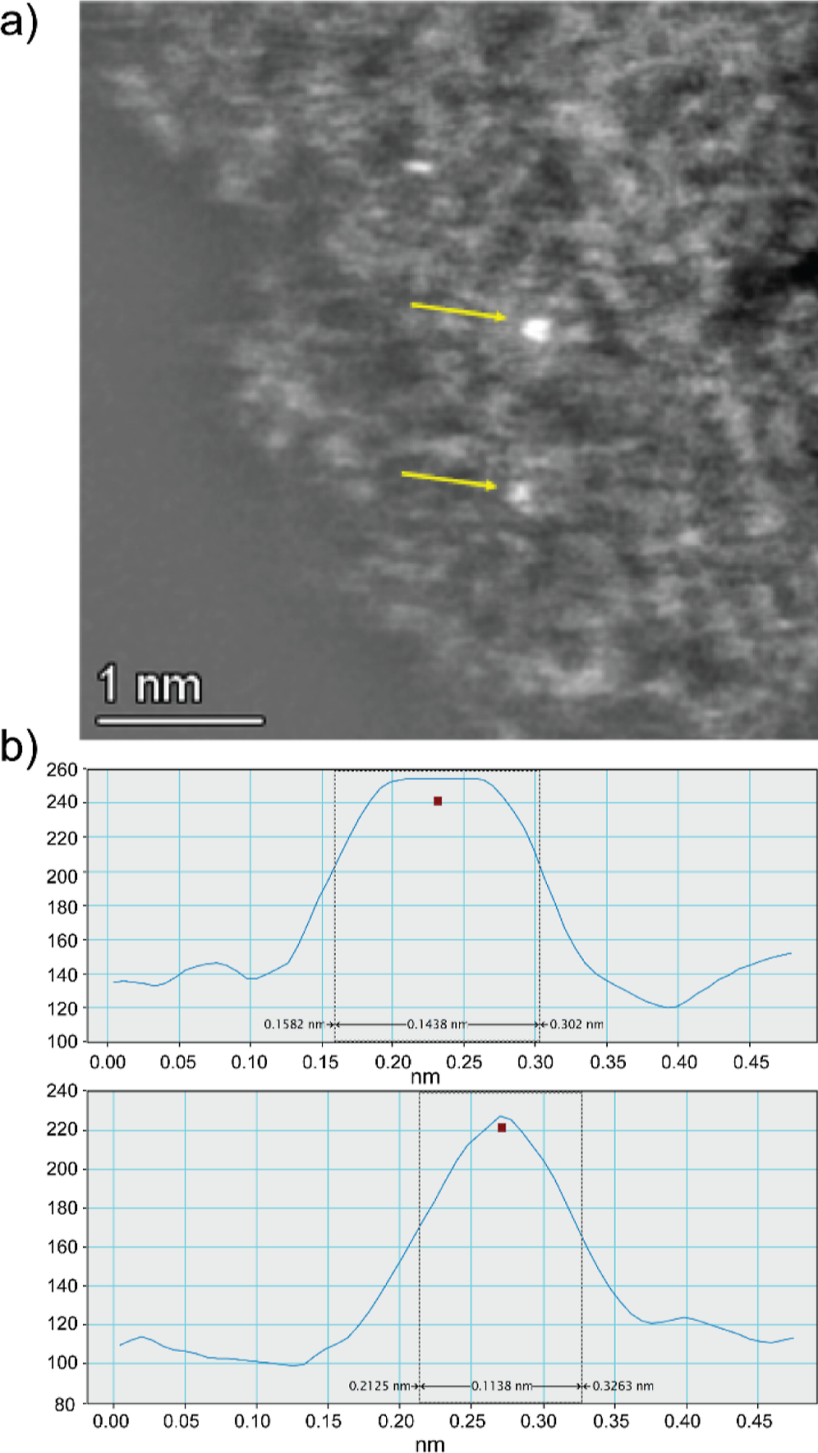
Magnified HAADF-STEM image of **Fe**^**3+**^**Ag**^**0**^_**2**_**@MOF**, with yellow arrows indicating the analyzed Ag aggregates (a), and the corresponding analysis of distances in the pointed Ag(0) aggregation (b).

In order to confirm the presence of single site Fe^3+^-SMAs, X-ray absorption near edge structure (XANES) and extended X-ray absorption fine structure (EXAFS) measurements were carried out. XANES spectra (Figure 4a) show that the more plausible oxidation state of Fe in **Fe**^**3+**^**Ag**^**0**^_**2**_**@MOF** is Fe^3+^, due to the presence of a first pre-edge peak at 7115 eV. The absorption edge of the MOF was closer to Fe_2_O_3_ (7133 eV for isolated Fe^3+^ atoms and 7134 eV for the standard). For the Fe K-edge EXAFS (Figure 4b), the peak at 1.47 Å can be attributed to Fe–O scattering, as the standard Fe_2_O_3_ samples indicate, while the peak corresponding to the Fe–Fe scattering path in Fe foil (2.21 Å) was not observed in **Fe**^**3+**^**Ag**^**0**^_**2**_**@MOF**, indicating the absence of clusters or nanoparticles. The absence of a peak at 3.17 Å in the Fe_2_O_3_ sample, but not in the MOF indicates the absence of Fe–O–Fe structures in the latter. Indeed, the result of the fit predicts a first shell of oxygen corresponding to the oxamate ligand, with a coordination number: 2.63 ± 0.21 at a distance of 1.97 Å (Table S3 and Figure S14 top). These results strongly support the presence of single-site Fe^3+^ atoms in **Fe**^**3+**^**Ag**^**0**^_**2**_**@MOF**. We also measured the Ag K-edge of the **Fe**^**3+**^**Ag**^**0**^_**2**_**@MOF** and compared it with different oxidation state standards (Ag foil and Ag_2_O). In this case, the signal for Ag in the MOF is clearly attributable to Ag(0) (Figure S14 middle). Unfortunately, due to the large time measurements required to obtain enough EXAFS scans (hours vs minutes in XANES), the beam light from the synchrotron could have reduced the Ag dimers forming bulk species, which does not allow us to see the dimer species inside the MOF (Figure S14 bottom).

### Catalytic Results

The direct conversion of styrene to phenylacetylene in one pot represents a challenge in organic synthesis. Here, we propose an approach based on the metal-catalyzed oxidative cross-coupling reaction of styrene **1a** with phenyl sulfone **2**, followed by the elimination of the sulfone moieties from intermediates vinyl sulfones **4a**, to lead to the targeted phenylacetylene **3a** (Figure 1b). Vinyl sulfones **4** have been previously prepared by a variety of methods.^50−54^ However, the engagement of **4a** with a base-mediated elimination to form the corresponding alkyne is not obvious with any of these methods. For instance, the synthesis of **4a** has been reported with large catalytic amounts of AgNO_3_/TEMPO [TEMPO: (2,2,6,6-tetramethylpiperidin-1-yl)oxyl, 15–20 mol %].^50^ Although this is incompatible with the elimination step (see below Table 2). Thus, there is a necessity to find novel catalysts able to circumvent the existing drawbacks on the synthesis of alkynes and, consequently, expand the library of available alkynes from readily available cheap starting materials.

The synthesis of vinyl sulfone **4a** from styrene **1a** and phenyl sulfone **2** was first studied with different homogenous metal catalysts, using TEMPO or not as an organic oxidant (Table 1, entries 1–9). As expected, we found that the oxidative coupling of styrene **1a** and phenyl sulfone **2** barely proceeded without AgNO_3_ or TEMPO (entries 1–2), but gave 74% of **4a** when both AgNO_3_ (15 mol %) and TEMPO (20 mol %) were combined (entry 3). Notice that these catalytic results were obtained under the optimized reaction conditions reported in the literature and following the reactants disappearance and product appearance by gas chromatography (GC). The roles of both catalysts are established, and while AgNO_3_ is the active coupling catalyst, TEMPO acts as a one-electron oxidant to expel the H atom and give the final alkene.^50^ Therefore, one-electron metal oxidants were then tested in order to explore the possibility of performing the coupling and oxidation processes in one-SMA and avoid TEMPO. However, different copper and iron metal salts were found not to be catalytically competent in solution (entries 4–7). Indeed, the combination of AgNO_3_ and Fe_2_(SO_4_)_3_ was not catalytically active (entry 8) even in the presence of TEMPO (entry 9), which evidences the incompatibility of both metals in solution, precluding Ag to independently play as a catalyst. This incompatibility plausibly comes from a redox reaction in solution, according to their very similar potentials (*E*^0^ ∼ +0.13 V), since Ag^0^ will rapidly oxidize to Ag^+^ and Fe^3+^ will rapidly reduce to Fe^2+^.

**Table 1:**
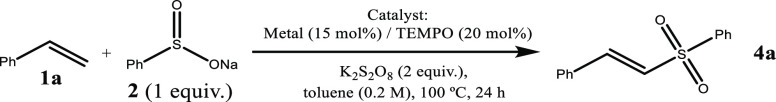
Catalytic Results for the Oxidative Cross-coupling Reaction of Styrene **1a** with Phenyl Sulfone **2**^a^

entry	catalyst	TEMPO	**4a** (yield, %)
1	none	yes	6
2	AgNO_3_	none	8
3		yes	74
4	CuCl_2_	none	7
5	Cu(OAc)_2_	none	6
6	Fe(acac)_3_	none	3
7	Fe_2_(SO_4_)_3_	none	28
8	AgNO_3_ + Fe_2_(SO_4_)_3_	none	5
9		yes	22
10	**Ag**^**0**^_**2**_**@MOF**	yes	48
11		none	45
12	Ni_2_@MOF	none	11
13	AgNO_3_ + Ni_2_@MOF	none	61
14	**Fe**^**3+**^**@MOF**	yes	63
15		none	61
16	**Ag**_**02**_**@MOF + Fe**^**3+**^**@MOF**	yes	53
17		none	73
18	**Fe**^**3+**^**Ag**^**0**^_**2**_**@MOF**	yes	73
19		none	95
20	Fe_2_(SO_4_)^3^ + **Ag**^**0**^_**2**_**@MOF**	none	6
21	AgNO_3_ + **Fe**^**3+**^**@MOF**	none	32

a GC yields; average of, at least, two runs. The total metal loading was consistently 15 mol %, i.e., 7.5 mol % of Ag plus 7.5 mol % of Fe when combined.

With all this information in hand, we extended our study to the heterogenous phase. First, we investigated the catalytic activity of **Ag**^**0**^_**2**_**@MOF**, possessing SNMCs Ag^0^_2_ within MOF channels. We observed that **Ag**^**0**^_**2**_**@MOF** showed moderate catalytic activity for the reaction (ca. 45%, entries 10–11 in Table 1), with and without TEMPO. The fact that **Ag**^**0**^_**2**_**@MOF** was able to catalyze the reaction without TEMPO can be explained by the co-catalysis exerted by the framework of the MOF itself, which was able to trigger the catalytic activity even with AgNO_3_ in solution (entries 12–13), but with moderate yields of **4a**. Besides, if one considers that TEMPO is only active with Ag in solution, we can reasonably accept that the co-catalysis exerted by the MOF framework is at least similar to or higher than that exerted by TEMPO; thus, the action of TEMPO did not have any influence on the **Ag**^**0**^_**2**_**@MOF** catalyst. Then, on the basis of the results obtained with homogeneous solutions of Fe_2_(SO_4_)_3_, we investigated the catalytic performance of **Fe**^**3+**^**@MOF**, obtaining moderately good yields (ca. 62%) in the presence or absence of TEMPO (entries 14–15), which evidenced the suitability of Fe^3+^-SMAs to substitute TEMPO. In this context, we envisioned the heterogenization of metallic species in **Fe**^**3+**^**Ag**^**0**^_**2**_**@MOF** would be an appealing candidate to catalyze efficiently the required chemical events for the oxidative cross-coupling of **1a** and **2**. In fact, **Fe**^**3+**^**Ag**^**0**^_**2**_**@MOF** showed a very efficient performance that exceeded not only the individual MOF-supported metallic entities (**Fe**^**3+**^**@MOF** and **Ag**^**0**^_**2**_**@MOF**, entries 10–11 and 14–15) but also a 1:1 physical mixture of them (entries 16–17) and any soluble catalyst under the same reaction conditions (entries 2–9), to give **4a** in 95% yield (entry 19). In addition, we further confirmed TEMPO is not needed for the reaction when the catalyst contains Fe^3+^-SMAs since it only hampers the final reaction yield for **Fe**^**3+**^**Ag**^**0**^_**2**_**@MOF** (compare entries 18–19). The decrease in catalytic activity by TEMPO from 95 to 73% is tentatively assigned to a partial deactivation of the Fe^3+^ sites (see ahead).

The superior catalytic performance of **Fe**^**3+**^**Ag**^**0**^_**2**_**@MOF** with respect to any other catalyst can be explained on the basis of its unique chemical structure, where isolated Fe^3+^-SMAs and Ag^0^_2_-SNMCs are firmly supported within the same MOF and, noteworthily, coexist at molecular distances. On one hand, the fixed position of the single site metallic entities avoids metal interaction and reactivity within the MOF channels, which explains the dramatic increase in catalytic action compared to the corresponding silver(I) and iron(III) salts in solution (5% yield of **4a**, entry 10). This point was further confirmed when we studied the reaction using one of the individual MOFs together with the corresponding salt of the other metal (Fe_2_(SO_4_)_3_ + **Ag**^**0**^_**2**_**@MOF** and AgNO_3_ + **Fe**^**3+**^**@MOF**, entries 20–21), where we found an abrupt decrease in the catalytic activity with respect to **Fe**^**3+**^**Ag**^**0**^_**2**_**@MOF**. In accordance with these results, the hot filtration test for **Fe**^**3+**^**Ag**^**0**^_**2**_**@MOF** showed that the catalytic activity completely stopped after the removal of the **Fe**^**3+**^**Ag**^**0**^_**2**_**@MOF** solid from the reaction mixture, after adding the insoluble K_2_S_2_O_8_ to the filtrates (Figure S15). Besides, the comparison between the mixing tests for Ag/Fe salts and MOFs (compare entries 8–9 and 16–17) also supports the synergistic effect in **Fe**^**3+**^**Ag**^**0**^_**2**_**@MOF**. Thus, one can soundly affirm that supporting both Fe^3+^-SMAs and Ag^0^_2_-SNMCs metallic entities within the MOFs walls is beneficial for having an efficient reaction. On the other hand, the proximity at the molecular distance between metal sites in **Fe**^**3+**^**Ag**^**0**^_**2**_**@MOF** is of key relevance to allow a much better co-catalysis. This was supported with the study of the 1:1 physical mixture of **Ag**^**0**^_**2**_**@MOF** + **Fe**^**3+**^**@MOF**—where the metallic entities in each of these materials are structurally identical to the ones in **Fe**^**3+**^**Ag**^**0**^_**2**_**@MOF** but just residing in distinct MOF particles, which gave a significantly lower yield of **4a** (73 vs 95%, compare entries 17 and 19) since intermediates must diffuse interparticle.

In view of the good matching in catalytic activity between AgNO_3_ (+TEMPO) and **Fe**^**3+**^**Ag**^**0**^_**2**_**@MOF**, we wondered if the true active Ag species in solution were also Ag^0^_2_, formed in situ under the reaction conditions.^64^ The formation of ultrasmall noble metal clusters in solution during organic reactions when starting from metal salts is well-established in the literature^19,33^ and is based on the tendency of the noble metal salt, without stabilizing ligands, to reduce and aggregate but, at the same time, to be stabilized in the form of metastable ultrasmall clusters by the action of the reactants/products. The monitoring of the reaction using AgNO_3_ and TEMPO (entry 3) by UV–vis absorption and emission spectrophotometry does not show the presence of plasmonic Ag nanoparticles (Figure S16), but the appearance of fluorescence bands that are compatible with the formation of Ag_2–10_ clusters during the reaction (Figure S17). This result was also supported by a kinetic study (Figure S18), which revealed that the initial reaction rate (v_0_) is exponentially dependent on the [Ag] and more precisely follows a linear dependence with [Ag].^2^ The complete kinetic study (Figures S19–S22) for the species involved in the coupling reaction gave the following rate equation, *v*_0_ = *k*_exp_[Ag]^2^[TEMPO][**2**][**1a**]^−1^. In other words, the reaction rate is first order with respect to Ag dimers, TEMPO, and vinyl sulfone **2**, shows zero dependence with the K_2_S_2_O_8_ oxidant, and inverse dependence with respect to styrene **1a**. Thus, the radical activation and coupling of **1a** with **2** seems to be involved in the rate-determining step of the reaction, since the reaction rate increases linearly with the concentrations of both co-catalysts and **2**, but inversely with the concentration of **1a**, which can be explained by the expected undesired radical reactions of alkene **1a** with TEMPO. Monitoring of the reaction without K_2_S_2_O_8_ by electronic paramagnetic resonance (EPR) experiments shows the consumption of TEMPO during the reaction (Figure S23), and the use of 5,5-dimethyl-1-pyrroline *N*-oxide (DMPO) as a spin trapping led to the unambiguous characterization of the DMPO adduct radicals DMPO-PhSO_2_ (*A*_N_ = 12.45 G and *A*_Hβ_ = 13.68 G)^65^ and DMPO-OOH (*A*_N_ = 12.73 G, *A*_Hβ_ = 6.56 G, and *A*_Hβ_ = 1.74 G),^66^ formed during the reaction according to the hyperfine parameters (Figure S24). The formation of the PhSO_2_·radical during the Ag-catalyzed reaction perfectly matches the proposed mechanism for the reaction,^50^ and the observation than that of hydroperoxide radicals HOO·are also formed during the reaction may explain the often observed formation of ketone by-products under air.^67,68^ These results are compatible with the role of Fe^3+^ as a substituting catalytic site for TEMPO in the **Fe**^**3+**^**Ag**^**0**^_**2**_**@MOF** solid catalyst.^69^ Indeed, the action of TEMPO on the separate or joined Fe^3+^/Ag^0^_2_ MOF sites is exactly the same (compare entries 16 and 18 in Table 1), which further validates the catalytic comparison. Here, we speculate that the proximity of the catalytic sites shortens diffusion issues and provides the necessary reactive environment to get to the product. This would be in accordance with the highly reactive nature of the radical intermediates found.

The oxidation step should occur lately since the reaction rate is not affected by the concentration of K_2_S_2_O_8_. In other words, AgNO_3_ evolves to catalytically active Ag^0^_2_ clusters (and other clusters of higher atomicity) under reaction conditions in solution (with TEMPO), which explains the superior catalytic activity of **Fe**^**3+**^**Ag**^**0**^_**2**_**@MOF** with pre-formed and stabilized Ag^0^_2_ sites, moreover considering that the proximity of the Ag^0^_2_ and Fe^3+^ catalytic sites is key for the reaction rate.

Once we confirmed the superiority of the hybrid assembly **Fe**^**3+**^**Ag**^**0**^_**2**_**@MOF** to obtain the intermediate vinyl-sulfone **4a**, we studied, independently, the elimination step that led to phenylacetylene **3a** under basic conditions (Table S4). The results show that KO^*t*^Bu (2 equiv) in tetrahydrofurane (THF, 1 M) solvent is the more effective system of those studied, which include different carbonates, phosphonates, and *tert*-butoxides in toluene or acetonitrile. At 70 °C, vinyl sulfone **4a** is completely converted to phenylacetylene **3a** in 1 h reaction time. We also found that commercially available KO^*t*^Bu in THF gives the same result as solid KO^*t*^Bu dispersed in THF, so both can be used indistinctly.

Then, with the treasured knowledge of the best catalyst and reaction conditions in hand, we proceed to study the one-pot conversion of styrene **1a** to phenylacetylene **3a** through intermediate vinyl-sulfone **4a** (Table 2). We observe that the one-pot synthesis of phenylacetylene **3a** from styrene **1a** was not possible when the soluble catalytic system AgNO_3_ + TEMPO is employed (entries 1–3), even if the remaining salts after the oxidative coupling of the toluene solvent are removed prior to KO^*t*^Bu treatment in THF. In contrast, **Fe**^**3+**^**Ag**^**0**^_**2**_**@MOF** allows the one-pot synthesis of phenylacetylene **3a** from styrene **1a**, after filtration of the catalyst and toluene removal, to give phenylacetylene **3a** together with some heavier molecules in >99% isolated yield—after water/ethyl acetate washings, drying, and solvent removal. Here, we would like to comment on the relevance of this result. In fact, it is difficult to find in the literature any procedure that directly converts an alkene to an alkyne in the same flask^70−72^ and, to our knowledge, a catalytic version of this transformation has only previously been reported with Pd supported in polyamine,^70^ which we could not reproduce in our hands. Besides, the **Fe**^**3+**^**Ag**^**0**^_**2**_**@MOF** catalyst could be reused after filtration, being able to be recycled up to 5 times while retaining moderately good yields (Figure S25). In fact, ICP–MS analyses (Table S1) and PXRD (Figure S26) and XPS (Figure S27) measurements for **Fe**^**3+**^**Ag**^**0**^_**2**_**@MOF**, after 5 consecutive cycles, confirm that the chemical formula remains unaltered (Table S1), and that no metal nanoparticles are formed during the catalytic experiments (Figure S26), indicating that Ag SNMCs and Fe SACs are still present and maintain the same oxidation states (Figure S27). Dealing with the structure of the heavier molecules, they could be assigned to the corresponding oligomers of alkyne **3a**, according to gas chromatography–mass spectrometry (GC–MS) analysis of the product mixture, in order to complete the mass balance. The **3a**/oligomer mixture was also characterized by FTIR (Figure S28) and ^1^H nuclear magnetic resonance (^1^H NMR, Figure S29), which confirmed the total conversion of the intermediate vinyl sulfone **4a** and the unambiguous formation of **3a** and the corresponding oligomers. Experiments with neat phenylacetylene **3a** under the KO^*t*^Bu conditions confirmed that the base-triggered oligomerization reaction is faster than the deprotonation/elimination of vinyl sulfone **4a**, which explains the irremediable appearance of these oligomers at the end of the reaction.^73−75^

**Table 2:**
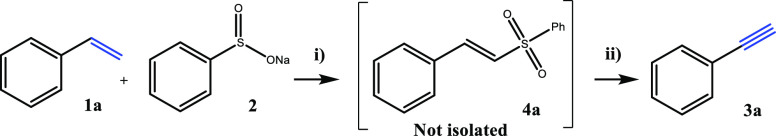
Results for the One-pot Synthesis of Phenylacetylene **3a** from Styrene **1a**, after Oxidative Cross-coupling Reaction with Phenyl Sulfone **2** and In Situ Base Treatment, under Optimized Conditions (see Tables 1 and S3)^a^

entry	coupling catalyst	filtration prior to KO^*t*^Bu reaction	solvent for KO^*t*^Bu	**3a** (yield, %)^b^
1	AgNO_3_ + TEMPO	no	toluene + THF	<5
2		yes		<5
3		yes	THF	<5
4	**Fe**^**3+**^**Ag**^**0**^_**2**_**@MOF**	no	toluene + THF	<5
5		yes		<5
6		yes	THF	>99

a GC yields; average of, at least, two runs.

b Isolated yield; some oligomers of **3a** were also found and are included in the yield.

**Figure 6:**
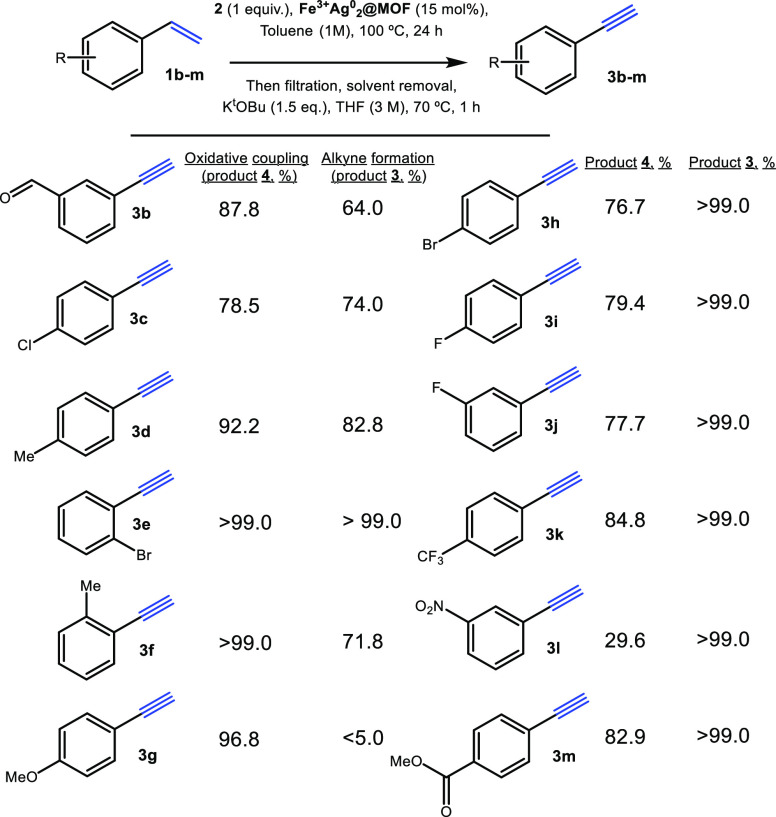
Results for the one-pot synthesis of alkynes **3b–m** from styrenes **1b–m** after the oxidative cross-coupling reaction with phenyl sulfone 2 catalyzed by **Fe**^**3+**^**Ag**^**0**^_**2**_**@MOF** and in situ KO^*t*^Bu treatment. GC yields for products **4b–m** and isolated yields for products **3b–m**, taking in account the oligomers formed.

Finally, we explored the performance of **Fe**^**3+**^**Ag**^**0**^_**2**_**@MOF** for the one-pot oxidative cross-coupling reaction of different styrenes **1b–m** with phenyl sulfone **2** and in situ KO^*t*^Bu treatment (Figure 6, see experimental data and NMR copies for intermediates **4b–m** in the Supporting Information; the final phenylacetylene products are characterized by GC–MS). It can be seen that ∼70% yields for different phenylacetylenes **3b–m** were consistently obtained, with a quantitative yield (>99%) for alkynes **3e** and **3h–m**. Thus, aldehyde, ester, nitro, halogen, and methyl substitutions in different positions of the aromatic ring are tolerated. In contrast, only the anisole derivative **4g** does not engage in the final elimination reaction; however, it reacts well (96.8%) during the **Fe**^**3+**^**Ag**^**0**^_**2**_**@MOF**-catalyzed oxidative coupling.

**Figure 7:**
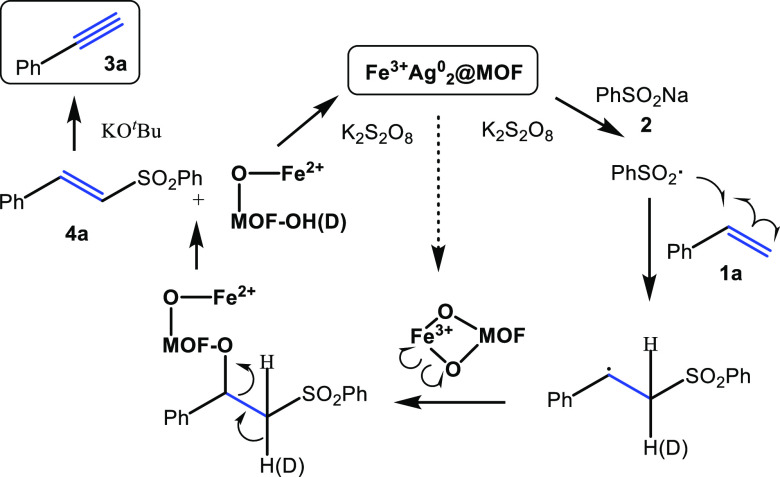
Proposed mechanism for the one-pot conversion of styrene **1a** to phenylacetylene **3a** through intermediate vinyl-sulfone **4a**, catalyzed by **Fe**^**3+**^**Ag**^**0**^_**2**_**@MOF**.

Figure 7 shows the proposed mechanism for the one-pot synthesis of phenylacetylene **3a** from styrene **1a** catalyzed by **Fe**^**3+**^**Ag**^**0**^_**2**_**@MOF**. The first step is the formation of the PhSO_2_·radical, as assessed above by EPR. This radical adds to styrene **1a** to form the corresponding radical benzyl adduct, which must eliminate the H atom in anti-position to form stereoselectively the observed *trans* vinyl-sulfone **4a**. This elimination step has been attributed in previous mechanisms^50^ to the action of TEMPO, and since the redox pair Fe^2+^/Fe^3+^ could be playing that role here, we envisaged that the Fe^3+^–O–MOF bonds could be homolytically broken during the elimination step. To check that, we performed the coupling reaction with isotopically labeled styrene-*d*8 (**1a–d**8 >98% deuterium incorporation) and without K_2_S_2_O_8_, to monitor the formation of the corresponding new OD bonds in the MOF by FT-IR spectroscopy. The results show the appearance of a new signal at ∼2750 cm^–1^, consistent with the formation of the new OD bond in the spent MOF (Figure S30). At the same time, a classical analytical test with KMnO_4_ showed the formation of Fe^2+^ in the same spent MOF catalysts,^76^ thus confirming the homolytic breaking of the MOF–O–Fe^3+^ bond. This step participates in the reaction rate according to the rate equation (see above) and the observation of a kinetic isotopic effect (KIE) = 1.3(9) (Figure S31). The unnecessity of TEMPO to catalyze the reaction was checked by performing the AgNO_3_-catalyzed reaction in acetonitrile rather than in toluene solution since acetonitrile is a privileged solvent for radical shuttling. The kinetic results show that the reaction proceeds without TEMPO (Figure S32), thus further supporting that alternative radical shuttles to TEMPO are possible under exactly the same reaction conditions. The metal catalytic sites in **Fe**^**3+**^**Ag**^**0**^_**2**_**@MOF** are then regenerated with K_2_S_2_O_8_ and alkyne **3a** is finally obtained after deprotonation of **4a**. Although the mechanism has been studied by a combination of results with the homogenous Ag catalyst and **Fe**^**3+**^**Ag**^**0**^_**2**_**@MOF**, we think that the results are valid since the mechanism in solution and in the solid should be similar, and this approach avoids the diffusion problems associated with the solid catalyst when calculating accurate measures such as the KIE.

## Conclusions

Ag dimers are formed within a MOF in the presence of Fe^3+^ sites after reduction with NaBH_4_ of the corresponding Ag^+^-exchanged material. Solid **Fe**^**3+**^**Ag**^**0**^_**2**_**@MOF** can be obtained in multigram amounts and is a superior catalyst for the TEMPO-free oxidative cross-coupling of styrenes **1** with phenyl sulfone **2**, to give vinyl sulfones **4** in yields up to >99%. It has been assessed here that Ag dimers are the truly catalytic active species during the reaction in solution catalyzed by AgNO_3_ and TEMPO. Vinyl sulfones **4** can be transformed, in situ, to the corresponding phenylacetylene products **3** in high yields, up to >99%, after filtrating the **Fe**^**3+**^**Ag**^**0**^_**2**_**@MOF** catalyst, which can be reused. Thus, a final **Fe**^**3+**^**Ag**^**0**^_**2**_**@MOF**-catalyzed, one-pot conversion of styrenes **1** to phenylacetylenes **3** is achieved. The results here presented also constitute a paradigmatic example of how the speciation of the true metal catalyst of an organic reaction in solution, combined with the synthesis of this metal species in well-defined solid catalysts, allows to perform the otherwise very difficult reaction in solution.

## Experimental Section

### Preparation of [Ag^0^_2_(Ag^0^)_1.44_Fe^III^_0.66_]@Na^I^_2_{Ni^II^_4_[Cu^II^_2_(Me_3_mpba)_2_]_3_}·63H_2_O (**Fe**^**3+**^**Ag**^**0**^_**2**_**@MOF**)

Well-formed dark green prisms of **Fe**^**3+**^**Ag**^**0**^_**2**_**@MOF**, which were suitable for XRD, were obtained in a three-step PS process:

First, crystals of Ni^II^_2_{Ni^II^_4_[Cu^II^_2_(Me_3_mpba)_2_]_3_}·54H_2_O (ca. 5 mg, 0.0015 mmol) were suspended, for 24 h, in 5 mL of AgNO_3_ aqueous solutions (1 mg, 0.006 mmol) until complete replacement of Ni^2+^ cations hosted in the pores by Ag^+^ ones (assessed by SEM). Then, the resulting material was resuspended in a (NH_4_)_2_Fe(SO_4_)_2_·6H_2_O water/methanol (1:1) solution (1.2 mg, 0.003 mmol) under aerobic conditions. The process was repeated several times, but the iron contents were identical to those obtained after 24 h. The crystals were isolated by filtration on paper and air-dried.

After this double PS process, the resulting crystals (ca. 5 mg) were soaked in a H_2_O/CH_3_OH (1:1) solution to which NaBH_4_, divided into 15 fractions (0.4 mmol of NaBH_4_ per mmol of MOF each), were added progressively in the space of 72 h. Each fraction was allowed to react for 1.5 h. After this period, samples were gently washed with a H_2_O/CH_3_OH solution and filtered on paper. Anal. calcd for Cu_6_Ni_4_Fe_0.66_Ag_3.44_Na_2_C_78_H_186_N_12_O_99_ (**Fe**^**3+**^**Ag**^**0**^_**2**_**@MOF**) (MW: 3946.29): C, 23.74; H, 4.75; N, 4.26. Found: C, 23.79; H, 4.73; N, 4.36. IR (KBr) ν: 3008, 2967 and 2923 cm^–1^ (C–H), 1611 cm^–1^ (C=O).

Alternatively, a multigram scale procedure was also carried out by using the same synthetic procedure but with greater amounts of both a powder sample of compound Ni^II^_2_{Ni^II^_4_[Cu^II^_2_(Me_3_mpba)_2_]_3_}·54H_2_O (5 g), AgNO_3_ (1 g), (NH_4_)_2_Fe(SO_4_)_2_·6H_2_O (1.2 g) and NaBH_4_ (ca. 3 g divided in 15 fractions), with the same successful results and a high yield (5.13 g, 96%). Anal. calcd (%) for Cu_6_Ni_4_Fe_0.66_Ag_3.44_Na_2_C_78_H_186_N_12_O_99_ (**Fe**^**3+**^**Ag**^**0**^_**2**_**@MOF**) (MW: 3946.29): C, 23.74; H, 4.75; N, 4.26. Found: C, 23.79; H, 4.73; N, 4.36. IR (KBr) ν: 3011, 2956 and 2917 cm^–1^ (C–H), 1607 cm^–1^ (C=O).

### Catalysis Details

Reaction procedure for oxidative styrene couplings with soluble catalysts: products **4** were prepared following the reaction scheme. Reagents **1** (1 equiv, 0.4 mmol) and **2** (1 equiv, 0.4 mmol) were introduced in a glass reactor equipped with a magnetic stirrer, together with K_2_S_2_O_8_ (2 equiv, 0.8 mmol), TEMPO (0.2 equiv, 0.08 mmol), AgNO_3_ (15% mol, 0.06 mmol), and 2 mL of toluene, and allowed to react overnight at 100 °C under N_2_. After the reaction is complete, the resulting mixture is quenched by the addition of water, extracted with dichloromethane, and dried over Na_2_SO_4_. The products obtained are characterized by GC–MS.

Reaction procedure for styrene couplings with solid MOF catalysts: reagents **1** (1 equiv, 0.05 mmol) and **2** (1 equiv, 0.05 mmol) were introduced in a small glass vial with a magnetic stirrer, together with K_2_S_2_O_8_ (2 equiv, 0.1 mmol), TEMPO (0.2 equiv, 0.01 mmol), **Fe**^**3+**^**Ag**^**0**^_**2**_**@MOF** (11 mg, 5 mol % Ag), and 0.25 mL of toluene, and allowed to react for 24 h at 100 °C under N_2_, after sealing the vial. When the reaction is complete, the mixture is filtrated to eliminate the catalyst, and the resulting liquid is extracted with dichloromethane and dried over Na_2_SO_4_. The products obtained are characterized by GC–MS. GC yields are obtained after using 1 equiv respect to the limiting agent of an external standard (typically *n*-dodecane) and referring the obtained areas to the standard, following the formula: yield (product) = [(area product/response factor product)/(area standard/response factor standard)] × 100.

Reaction procedure for vinyl sulfone conversion to phenylacetylenes with KO^t^Bu: products **3** were prepared following the reaction scheme. Reagent **4** (1 equiv, 0.12 mmol) was introduced in a glass reactor equipped with a magnetic stirrer and a solution of KO^t^Bu (1 M) in THF (2 equiv, 0.24 mmol) at 70 °C. When the reaction finishes, water is added, and the mixture is extracted with THF and dried over Na_2_SO_4_. The products obtained are characterized by GC–MS.

Typical reaction procedures for catalyst reuse. Reuses of the **Fe**^**3+**^**Ag**^**0**^_**2**_**@MOF** solid catalyst were performed after separating the solids at the end of the reaction by centrifugation, and washing the solid mixture with deionized water and methanol (three times) to remove excess reagent **2**, TEMPO, K_2_S_2_O_8_, and any soluble product. Subsequently, the **Fe**^**3+**^**Ag**^**0**^_**2**_**@MOF** solid catalyst is dried under vacuum and directly used in the next reaction.

Hot-filtration test: following the general reaction procedure above, the hot reaction mixture was filtered through a 0.25 μm Teflon filter into a new glass reactor containing the insoluble **2** and K_2_S_2_O_8_ reagents and equipped with a magnetic stirrer. The mixture was placed at the reaction temperature, and the filtrates were periodically analyzed by GC to compare with the results obtained with the solid catalyst still in.

Reaction procedure for one-pot conversion of styrenes **1** to phenylacetylenes **3**: reagents **1** (1 equiv, 0.05 mmol) and **2** (1 equiv, 0.05 mmol) were introduced in a glass vial equipped with a magnetic stirrer, together with **Fe**^**3+**^**Ag**^**0**^_**2**_**@MOF** (11 mg, 5 mol % Ag), K_2_S_2_O_8_ (2 equiv, 0.1 mmol), and 0.25 mL of toluene and allowed to react for 24 h at 100 °C under N_2_. After the reaction is complete, filtration is carried out to remove the catalyst. The solution obtained is concentrated under vacuum and then introduced in a glass reactor equipped with a magnetic stirrer, with the help of some THF solvent if necessary. A solution of KO^*t*^Bu (1 M) in THF (2 equiv, 0.1 mmol) is then added, and the reaction is stirred at 70 °C for 1 h. When the reaction finishes, water is added, the mixture is extracted with THF, and it is dried over Na_2_SO_4_. Products **4** are characterized by GC–MS.

### X-ray Crystallographic Details

Diffraction data for **Fe**^**3+**^**Ag**^**0**^_**2**_**@MOF** was collected on a Bruker-Nonius X8APEXII CCD area detector diffractometer using graphite-monochromated Mo Kα radiation (λ = 0.71073 Å). Crystal data for **Fe**^**3+**^**Ag**^**0**^_**2**_**@MOF**: tetragonal, space group *P*4/*mmm*, *T* = 150(2), *Z* = 4. C_78_H_186_Ag_3.44_Fe_0.66_Na_2_Cu_6_Ni_4_N_12_O_99_, *a* = 35.8023(16) Å, *c* = 15.2143(9) Å, *V* = 19502(2) Å^3^. Further details can be found in the Supporting Information. CCDC 2157534 contains the supplementary crystallographic data for this paper. These data can be obtained free of charge via www.ccdc.cam.ac.uk/data_request/cif, by emailing data_request@ccdc.cam.ac.uk, or by contacting The Cambridge Crystallographic Data Centre, 12 Union Road, Cambridge CB2 1EZ, UK; fax: +44 1223 336033.

### X-ray Powder Diffraction Measurements

Polycrystalline samples of **Fe**^**3+**^**@MOF**, **Ag**^**0**^_**2**_**@MOF and Fe**^**3+**^**Ag**^**0**^_**2**_**@MOF** were introduced into 0.5 mm borosilicate capillaries prior to being mounted and aligned on an Empyrean PANalytical powder diffractometer using Cu Kα radiation (λ = 1.54056 Å). For each sample, five repeated measurements were collected at room temperature (2θ = 2–60°) and merged into a single diffractogram. A polycrystalline sample of **Fe**^**3+**^**Ag**^**0**^_**2**_**@MOF** was also measured after catalysis following the same procedure.

### XPS Measurements

A sample of **Fe**^**3+**^**Ag**^**0**^_**2**_**@MOF** was prepared by sticking, without sieving, the MOF onto a molybdenum plate with scotch tape film, followed by air drying. Measurements were performed on a K-Alpha X-ray photoelectron spectrometer (XPS) system using a monochromatic Al K(alpha) source (1486.6 eV). As an internal reference for the peak positions in the XPS spectra, the C 1s peak has been set at 284.8 eV.

### Thermogravimetric Analysis

TGA was performed on membrane samples under a dry N_2_ atmosphere with a Mettler Toledo TGA/STDA 851^e^ thermobalance. The experiments were carried out within a temperature range of 25 up to 800 °C at a heating rate of 10 K/min. Approximately, 20 mg of the membrane was placed in a ceramic pan for the measurements.

### Microscopy Measurements

SEM elemental analysis was carried out for **Fe**^**3+**^**Ag**^**0**^_**2**_**@MOF** using a HITACHI S-4800 electron microscope coupled with an EDX detector. Data was analyzed with QUANTAX 400.

### Gas Adsorption

The N_2_ adsorption–desorption isotherms at 77 K were carried out on polycrystalline samples of **Fe**^**3+**^**@MOF**, **Ag**^**0**^_**2**_**@MOF**, and **Fe**^**3+**^**Ag**^**0**^_**2**_**@MOF** with a BELSORP-mini-X instrument. Samples were first activated with methanol and then evacuated at 348 K during 19 h under 10^–6^ Torr prior to their analysis.

### UV–Vis Absorption and UV–Visible Emission (Fluorimetry) Spectrophotometry

The photophysical measurements were performed under air at room temperature in a quartz cell of 1.0 cm optical path length. Absorption spectra were recorded on a Cary 300 UV–vis spectrophotometer (UV0811M209, Varian) and fluorescence spectra were obtained with a LP S-220B (Photon Technology International) equipped with a 75 W Xe lamp.

### Electronic Paramagnetic Resonance

The EPR measurements were performed at −170 °C using an EMX-12 Bruker spectrometer working at the X band, with a frequency modulation of 100 kHz and 1 G amplitude. Portions at different times of each reactions were introduced inside an EPR quartz probe cell and were measured.

### X-ray Absorption Spectroscopy

Measurements were carried out on the CLAESS beamline at the ALBA Synchrotron Light Source, Barcelona (Spain). Together with the samples, several standard references (Fe foil, Fe_2_O_3_, Ag foil, and Ag_2_O) have been finely powdered, uniformly mixed with cellulose, and pressed into pellets to ensure the correct absorption jump in fluorescence. Data reduction has been done using the Demeter program suite: raw data has been normalized by subtracting and dividing pre-edge and post-edge backgrounds as low-order polynomial smooth curves. By assuming a linear dependency between the “white line” intensity (taken at the zero of the derivative spectra) and the corresponding electron valence (known for the set of reference compounds), we estimated the oxidation state of the sample. The local structure of the sample has been then refined using the EXAFS signal in the *k* range 3: 12 Å^–1^.

### Aberration-Corrected High-Angle Annular Dark-Field Scanning Transmission Electron Microscopy

High resolution transmission electron microscopy measurements were performed in a double-aberration-corrected, monochromated, FEI Titan3 Themis 60–300 microscope working at 300 kV, by impregnating a gold filmed grid (Cu grids were not employed to measure the Cu content in the MOF) with a drop of **Fe**^**3+**^**Ag**^**0**^_**2**_**@MOF** dispersed in dichloromethane and leaving evaporation for at least 5 h. The microscope was also used to perform chemical mapping using the high-efficiency SuperX G2 detection system equipped in the microscope, which integrates four windowless detectors surrounding the sample and high-performance signal-processing hardware.

## Supporting Information Available

Physical techniques; crystallographic and catalytic details; crystal structure; PXRD pattern profiles; SEM images; TGA; XPS; N_2_ sorption and desorption isotherms; fluorescence measurements; HAADF-STEM images; EXAFS; hot filtration test; UV-vis absorption spectra; UV-vis emission spectra; kinetic plot; EPR spectra; FT-IR spectra; ^1^H-NMR; kinetics of reactions; data from the ICP–MS and SEM/EDX analyses; crystallographic data; Fe K-edge EXAFS fitting results; results for the synthesis of alkyne 3a from vinyl sulfone 4a; characterization of isolated compounds; and NMR spectra (PDF)
CCDC reference number: 2157534 for **Fe**^**3+**^**Ag**^**0**^_**2**_**@MOF**.
