# Supplementary material for: MOF-Triggered Synthesis of Subnanometer Ag02 Clusters and Fe3+ Single Atoms: Heterogenization Led to Efficient and Synergetic One-Pot Catalytic Reactions
Source: J Am Chem Soc. 2023 Apr 28;145(18):10342–54. doi: 10.1021/jacs.3c02155 (PMC10176469; doi:10.1021/jacs.3c02155)
Supplement: Supplementary file 1 — ja3c02155_si_001.pdf [file ja3c02155_si_001.pdf]

Supporting Information (SI) for the manuscript:

MOF-triggered Synthesis of Subnanometer Ag^0_2 clusters and Fe^{3+} Single Atoms: Heterogenization Led to Efficient and Synergetic One-pot Catalytic Reactions

Estefanía Tiburcio,[†,◊] Yongkun Zheng,[‡,◊] Cristina Bilanin,[‡,◊] Juan Carlos Hernández–Garrido,[♣] Alejandro Vidal–Moya,[‡] Judit Oliver–Meseguer,[‡] Nuria Martín,[†] Marta Mon,[*,‡] Jesús Ferrando-Soria,[*,†] Donatella Armentano,[*,♠] Antonio Leyva–Pérez,[*,‡] and Emilio Pardo[*,†]

[†]Instituto de Ciencia Molecular (ICMol), Universidad de Valencia, 46980 Paterna, Valencia, Spain

[‡]Instituto de Tecnología Química (UPV–CSIC), Universitat Politècnica de València–Consejo Superior de Investigaciones Científicas, Avda. de los Naranjos s/n, 46022 Valencia, Spain

[♣]Departamento de Ciencia de los Materiales e Ingeniería Metalúrgica y Química Inorgánica, Facultad de Ciencias, Universidad de Cádiz, Campus Universitario Puerto Real, 11510 Puerto Real, Cádiz, Spain.

[♠]Dipartimento di Chimica e Tecnologie Chimiche (CTC), Università della Calabria, Rende 87036, Cosenza, Italy

[◊]These authors equally contributed to the work.

Table of Contents

Experimental Section SI-3

Chemicals SI-3

Physical Techniques SI-4

X-ray crystallographic data SI-6

Catalysis SI-8

Figures S1-S32 SI-11

Tables S1-S4 SI-43

Characterization of isolated compounds SI-47

NMR spectra SI-51

Experimental Section

Chemicals

All chemicals were of reagent grade quality. They were purchased from commercial sources and used as received. $Ni_2^{II}\{Ni_4^{II}[Cu_2^{II}(Me_3mpba)_2]_3\} \cdot 54H_2O$, $[Fe^{III}(H_2O)_6][Fe_2^{III}(\mu\text{–}O)_2(H_2O)_6]_{1/2}\{Ni^{II}_4[Cu^{II}_2(Me_3mpba)_2]_3\} \cdot 72H_2O$ (**Fe^{3+}@MOF**) and $[Ag^0_2]$@$Ag^I_2Na^I_2\{Ni^{II}_4[Cu^{II}_2(Me_3mpba)_2]_3\} \cdot 48H_2O$ (**Ag^0_2@MOF**) were prepared as reported earlier.[1]

$[Ag^0_2(Ag^0)_{1.44}Fe^{III}_{0.66}]$@$\{Ni^{II}_4[Cu^{II}_2(Me_3mpba)_2]_3\} \cdot 63H_2O$ (**$Fe^{3+}Ag^0_2$@MOF**) : Well-formed dark green prisms of **$Fe^{3+}Ag^0_2$@MOF**, which were suitable for X-ray diffraction, were obtained in a three-step PS process:

First, crystals of $Ni^{II}_2\{Ni^{II}_4[Cu^{II}_2(Me_3mpba)_2]_3\} \cdot 54H_2O$ (*ca.* 5 mg, 0.0015 mmol) were suspended, for 24 hours, in 5 mL of $AgNO_3$ aqueous solutions (1 mg, 0.006 mmol), until complete replacement of Ni^{2+}, cations hosted in the pores, by Ag^+ ones (assessed by SEM). Then, the resulting material was resuspended in a $(NH_4)_2Fe(SO_4)_2 \cdot 6H_2O$ water/methanol (1:1) solution (1.2 mg, 0.003 mmol) under aerobic conditions. The process was repeated several times but the iron contents were identical to those obtained after 24 h. The crystals were isolated by filtration on paper and air-dried.

After this double PS process, the resulting crystals (*ca.* 5 mg) were soaked in a H_2O/CH_3OH (1:1) solution to which $NaBH_4$, divided in 15 fractions (0.4 mmol of $NaBH_4$ per mmol of MOF each), were added progressively in the space of 72 hours. Each fraction was allowed to react for 1.5 h. After this period, samples were gently washed with a H_2O/CH_3OH solution and filtered on paper. Anal.: calcd for $Cu_6Ni_4Fe_{0.66}Ag_{3.44}Na_2C_{78}H_{186}N_{12}O_{99}$ (**$Fe^{3+}Ag^0_2$@MOF**) (MW: 3946.29): C, 23.74; H, 4.75; N, 4.26. Found: C, 23.79; H, 4.73; N, 4.36. IR (KBr): ν = 3008, 2967 and 2923 cm^{-1} (C–H), 1611 cm^{-1} (C=O).

Alternatively, a multigram scale procedure was also carried out by using the same synthetic procedure but with greater amounts of both, a powder sample of compound

[1] (a) Grancha, T.; Ferrando-Soria, J.; Zhou, H.-C.; Gascon, J.; Seoane, B.; Pasán, J.; Fabelo, O.; Julve, M.; Pardo, E. *Angew. Chemie Int. Ed.* **2015**, *54* (22), 6521–6525. (b) Tejeda-Serrano, M.; Mon, M.; Ross, B.; Gonell, F.; Ferrando-Soria, J.; Corma, A.; Leyva-Pérez, A.; Armentano, D.; Pardo, E. *J. Am. Chem. Soc.* **2018**, *140* (28), 8827–8832. (c) Tiburcio, E.; Zheng, Y.; Mon, M.; Martín, N.; Ferrando−Soria, J.; Armentano, D.; Leyva−Pérez, A.; Pardo, E. *Inorg. Chem.* **2022**, *61* (30), 11796–11802.

$Ni^{II}_2\{Ni^{II}_4[Cu^{II}_2(Me_3mpba)_2]_3\} \cdot 54H_2O$ (5 g), $AgNO_3$ (1 g), $(NH_4)_2Fe(SO_4)_2 \cdot 6H_2O$ (1.2 g) and $NaBH_4$ (*ca.* 3 g divided in 15 fractions), with the same successful results and a high yield (5.13 g, 96%). Anal.: calcd (%) for $Cu_6Ni_4Fe_{0.66}Ag_{3.44}Na_2C_{78}H_{186}N_{12}O_{99}$ (**$Fe^{3+}Ag^0_2$@MOF**) (MW: 3946.29): C, 23.74; H, 4.75; N, 4.26. Found: C, 23.88; H, 4.72; N, 4.23. IR (KBr): ν = 3011, 2956 and 2917 cm^{-1} (C–H), 1607 cm^{-1} (C=O).

Physical Techniques

Elemental (C, H, N), and ICP-MS analyses were performed at the Microanalytical Service of the Universitat de València. FT–IR spectra were recorded on a Perkin-Elmer 882 spectrophotometer as KBr pellets. The thermogravimetric analysis was performed on crystalline samples under a dry N_2 atmosphere with a Mettler Toledo TGA/STDA 851e thermobalance operating at a heating rate of 10 $^oC\ min^{-1}$.

X–ray Powder Diffraction Measurements: Polycrystalline samples of **Fe^{3+}@MOF, Ag^0_2@MOF** and **$Fe^{3+}Ag^0_2$@MOF** were introduced into 0.5 mm borosilicate capillaries prior to being mounted and aligned on a Empyrean PANalytical powder diffractometer, using Cu Kα radiation (λ = 1.54056 Å). For each sample, five repeated measurements were collected at room temperature (2θ = 2–60°) and merged in a single diffractogram. A polycrystalline sample of **$Fe^{3+}Ag^0_2$@MOF** was also measured after catalysis following the same procedure.

X–ray photoelectron spectroscopy (XPS) measurements: Samples of **$Fe^{3+}Ag^+$@MOF** and **$Fe^{3+}Ag^0_2$@MOF** were prepared by sticking, without sieving, the MOF onto a molybdenum plate with scotch tape film, followed by air drying. Measurements were performed on a K-Alpha™ X-ray Photoelectron Spectrometer (XPS) System using a monochromatic Al K(alpha) source (1486.6 eV). As an internal reference for the peak positions in the XPS spectra, the C1s peak has been set at 284.8 eV.

Thermogravimetric Analysis: The thermogravimetric analysis was performed on polycrystalline samples under a dry N_2 atmosphere with a Mettler Toledo TGA/STDA 851^e thermobalance. The experiments were carried out within a temperature range from 25 °C up to 800 °C at a heating rate of 10 K/min. Approximately 20 mg of the membrane was placed in a ceramic pan for the measurements.

Microscopy measurements: Scanning Electron Microscopy (SEM) elemental analysis was carried out for **$Fe^{3+}Ag^{0}_{2}$@MOF**, using a HITACHI S-4800 electron microscope coupled with an Energy Dispersive X–ray (EDX) detector. Data was analyzed with QUANTAX 400.

Gas adsorption: The N_2 adsorption-desorption isotherms at 77 K, were carried out, on polycrystalline samples of **Fe^{3+}@MOF, Ag^{0}_{2}@MOF** and **$Fe^{3+}Ag^{0}_{2}$@MOF** with a BELSORP-mini-X instrument. Samples were first activated with methanol and then evacuated at 348 K during 19 hours under 10^{-6} Torr prior to their analysis.

UV–Vis absorption and UV–Visible emission (fluorimetry) spectrophotometry: The photophysical measurements were performed under air at room temperature in a quartz cell of 1.0 cm optical path length. Absorption spectra were recorded on a Cary 300 UV–Vis spectrophotometer (UV0811M209, Varian) and fluorescence spectra were obtained with a LP S–220B (Photon Technology International) equipped with 75 W Xe lamp.

Electronic paramagnetic resonance (EPR) measurements: The EPR measurements were performed at -170 ºC using an EMX-12 Bruker spectrometer working at the X band, with a frequency modulation of 100 kHz and 1 G amplitude. Portions at different times of each reactions were introduced inside an EPR quartz probe cell and were measured.

X-ray absorption spectroscopy (XAS) measurements were carried out on CLAESS beamline at ALBA Synchrotron Light Source, Barcelona (Spain). Together with the samples, several standard references (Fe foil, Fe_2O_3, Ag foil and Ag_2O) have been finely powdered, uniformly mixed with cellulose, and pressed in pellets to ensure the correct absorption jump in fluorescence. Data reduction has been done using the Demeter program suit: raw data has been normalized by subtracting and dividing pre-edge and post-edge backgrounds as low order polynomial smooth curves. By assuming a linear dependency between the "white line" intensity (taken at the zero of the derivative spectra) and the corresponding electron valence (known for the set of reference compounds), we estimated the oxidation state of the sample. The local structure of the sample has been than refined using the EXAFS signal in the k range 3:12 $Å^{-1}$.

Aberration-corrected high–angle annular dark-field scanning transmission electron microscopy (AC-HAADF-STEM) measurements were performed in a double-aberration-corrected, monochromated, FEI Titan3 Themis 60–300 microscope working at 300 kV, by impregnating a gold filmed grid (Cu grids were not employed to measure the Cu content in the MOF) with a drop of

$Fe^{3+}Ag^{0}_{2}$@MOF dispersed in dichloromethane and leaving evaporation for at least 5 hours. The microscope was also used to perform chemical mapping using the high-efficiency SuperX G2 detection system equipped in the microscope, which integrates four windowless detectors surrounding the sample and high-performance signal-processing hardware.

X-ray crystallographic data collection and structure refinement.

Crystal of **$Fe^{3+}Ag^{0}_{2}$@MOF** with *ca.* 0.10 x 0.12 x 0.12 mm as dimensions was selected and mounted on a MITIGEN holder in Paratone oil and very quickly placed on a liquid nitrogen stream cooled at 150 K to avoid the possible degradation upon dehydration. Diffraction data were collected on a Bruker-Nonius X8APEXII CCD area detector diffractometer using graphite-monochromated Mo-K_{α} radiation (λ = 0.71073 Å). The data were processed through SAINT[2] reduction and SADABS[3] multi-scan absorption software. The structure was solved with the SHELXS structure solution program, using the Patterson method. The model was refined with version 2018/3 of SHELXTL against F^2 on all data by full-matrix least squares.[4]

Crystals of **3**, suitable for X-ray diffraction, were obtained after the three-step PS process reported at page 3 of SI. For that reason, it is reasonable to observe a diffraction pattern sometimes affected by expected internal imperfections of the crystals. Furthermore, considering the huge cell in which compound crystallize and high porosity of the system, it is reasonable to observe a quite poor diffraction power of the samples even if in presence of heavy atoms as copper, nickel, iron and silver. In fact, a completeness of data was obtained at θ_{max} of 21°, (Table S1) (detected as Alerts A in the checkcifs). However, the solution and refinement parameters are suitable, compared with MOFs structures generally reported, thus we are convinced that the model structure found is consistent.

In the refinement non-hydrogen were refined anisotropically except disordered Fe^{3+}, Na^{+} and Ag^{+} ions and lattice water molecules. All attempts to perform improved measurements on a single crystal of **$Fe^{3+}Ag^{0}_{2}$@MOF**, resulting after a crystal-to-crystal transformation and featuring a very huge cell, either at I19 beamline of DIAMOND or in-house X-ray facilities failed, due to partial crystal damage / crystal deterioration under reduction conditions. The occupancy factors, of both Fe^{3+} and Ag^{+} ions have been defined in agreement with SEM results. The use of some C-C bond lengths restrains, SIMU

[2] SAINT, version 6.45, Bruker Analytical X-ray Systems, Madison, WI, 2003.
[3] Sheldrick G.M. SADABS Program for Absorption Correction, version 2.10, Analytical X-ray Systems, Madison, WI, 2003
[4] (a) G. M. Sheldrick, *Acta Cryst.* **2015**, *C71*, 3-8. (b) G. M. Sheldrick, *Acta Cryst.* **2008**, *A64*, 112-122.

and DELU, SADI, DFIX and FLAT during the refinement has been reasonable imposed and related to flexibility of the three–substituted phenyl rings of the Me_3mpba ligand that are dynamic components of the frameworks. In the refinement, some further restrains, to make the refinement more efficient, have been applied. For instance, ADP components have been restrained to be similar to other related atoms, EADP for group of atoms of the guest Fe^{3+} and Ag^+ ions expected to have essentially similar ADPs.

The occupancy factors of Ag atoms and Fe^{3+} ions have been defined in agreement with their thermal factors and SEM results [0.1667 for Fe1, 0.1075 for Ag1/Ag2 and 0.1250 for Ag3. Furthermore, it is important to underline that, depending to their occupancy, Fe^{3+} metal ions are statistically disordered with Ag^0_2 dimers, exhibiting a random distribution with a population of 34 and 67% respectively.

The solvent molecules were disordered, only the molecules interaction with copper metal ions and in part with sodium ions have been in some way modelled. For that reason, in refinement, the contribution to the diffraction pattern from the disordered water molecules located in the voids was subtracted from the observed data through the SQUEEZE method, implemented in PLATON.[5] The hydrogen atoms of the ligand were set in calculated positions and refined as riding atoms whereas for detected water molecules were neither found nor calculated.

Overall the “Alert A” notifications found in the validation program CheckCIF are also related to intrinsic imperfections (as the presence of large outliers in the data set) quite normal for crystals that suffered a single–crystal to single–crystal process and disorder. They are unavoidable due to the expected severe disorder of both solvent and guest molecules. The comments for the main alerts A and B are described in the CIF using the validation reply form (vrf).

A summary of the crystallographic data and structure refinement for the **$Fe^{3+}Ag^0_2$@MOF** compound is given in Table S1. Indeed, the somewhat high R values is, most likely, also affected by the contribution of the highly disordered solvent to the intensities of the low angle reflections. CCDC 2157534.

[5] Spek, A. L. *Acta Crystallogr. Sect. D, Biol. Crystallogr.* **2009**, *65*, 148.

The final geometrical calculations on free voids (total potential solvent accessible void volume of 10156.2 Å^3 accounting for 52% of the cell volume) and the graphical manipulations were carried out with PLATON[7] implemented in WinGX,[6] and CRYSTAL MAKER[7] programs, respectively.

<u>*Catalysis details:*</u>

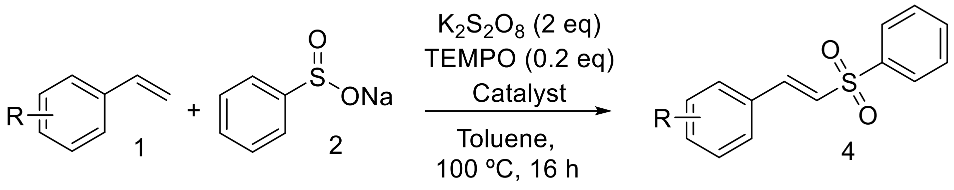

Reaction procedure for oxidative styrene couplings with soluble catalysts. Products **4** were prepared following the reaction scheme. Reagent **1** (1 eq, 0.4 mmol) and **2** (1 eq, 0.4 mmol) were introduced in a glass reactor equipped with a magnetic stirrer, together with $K_2S_2O_8$ (2 eq, 0.8 mmol), TEMPO (0.2 eq, 0.08 mmol), $AgNO_3$ (15% mol, 0.06 mmol) and 2 mL of toluene, and allowed to react overnight at 100 ºC under N_2, after sealing the reactor. When reaction is complete, the resulting mixture is quenched by addition of water, extracted with dichloromethane and dried over Na_2SO_4. The products obtained are characterized by GC-MS. GC yields are obtained after using one equivalent respect to the limiting agent of an external standard (typically *n*-dodecane) and referring the obtained areas to the standard, following the formula: yield (product)= [(area product / response factor product) / (area standard / response factor standard)] x 100.

Reaction procedure for styrene couplings with solid MOF catalysts. Reagent **1** (1 eq, 0.05 mmol) and **2** (1 eq, 0.05 mmol) were introduced in a small glass vial with a magnetic stirrer, together with $K_2S_2O_8$ (2 eq, 0.1 mmol), TEMPO (0.2 eq, 0.01 mmol), **$Fe^{3+}Ag^0_2$@MOF** (11 mg, 5 mol% Ag) and 0.25 mL of toluene, and allowed to react for 24 h at 100 ºC under N_2. When a combination of **Fe^{3+}@MOF** (1.5 wt%) + **Ag^0_2@MOF** (4.4 wt%) was used, the amounts added of each catalyst were 11.5 mg (6.0 mol% Fe) and 11 mg (9.0 mol% Ag), to keep a total 15 mol% metal amount. After reaction is complete, the

[6] Farrugia, L. J. *J. Appl. Crystallogr.* **1999**, *32*, 837.
[7] D. Palmer, CRYSTAL MAKER, Cambridge University Technical Services, C. No Title, 1996.

mixture is filtrated to eliminate the catalyst, and the resulting liquid is extracted with dichloromethane and dried over Na_2SO_4. The products obtained are characterized by GC-MS.

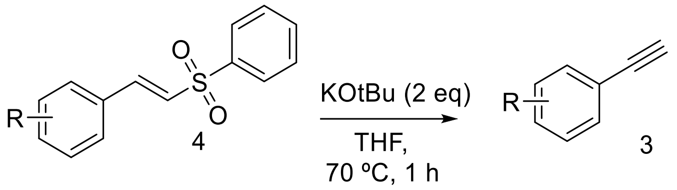

Reaction procedure for vinyl sulfone conversion to phenylacetylenes with KO^{t}Bu. Products **3** were prepared following the reaction scheme. Reagent **4** (1 eq, 0.12 mmol) was introduced in a glass reactor equipped with a magnetic stirrer and with a solution of KO^{t}Bu (1M) in THF (2 eq, 0.24 mmol) at 70° C. When the reaction finishes, water is added, the mixture is extracted with THF and dried over Na_2SO_4. The products obtained are characterized by GC-MS.

Typical reaction procedure for catalyst reuse. Reuses of the **$Fe^{3+}Ag^0{}_2$@MOF** solid catalyst were performed after separating the solids at the end of reaction by centrifugation, and washing the solid mixture with deionized water and methanol (three times) to remove excess reagent **2**, TEMPO, $K_2S_2O_8$, and any soluble product. Subsequently, the **$Fe^{3+}Ag^0{}_2$@MOF** solid catalyst is dried under vacuum and directly use in the next reaction.

Hot-filtration test. Following the general reaction procedure above, the hot reaction mixture was filtered, at intermediate conversion, through a 0.25 μm Teflon filter. Filtrates were placed into a new glass reactor equipped with a magnetic stirrer and fresh insoluble reactant **2** and $K_2S_2O_8$, and placed at the reaction temperature. The filtrates were periodically analyzed by GC, comparing the results obtained with the solid catalyst still in.

*Reaction procedure for one-pot conversion of styrenes **1** to phenylacetylenes **3**.* Reagents **1** (1 eq, 0.05 mmol) and **2** (1 eq, 0.05 mmol) were introduced in a glass vial equipped with a magnetic stirrer, together with **$Fe^{3+}Ag^0{}_2$@MOF** (11 mg, 5 mol% Ag), $K_2S_2O_8$ (2 eq, 0.1 mmol) and 0.25 mL of toluene, and allowed to react for 24 h at 100 ºC under N_2. After the reaction is complete, filtration is carried out

to remove the catalyst. The solution obtained is concentrated under vacuum, and then introduced in a glass reactor equipped with a magnetic stirrer, with the help of some THF solvent if necessary. A solution of KO^{t}Bu (1 M) in THF (2 eq, 0.1 mmol) is then added, and the reaction stirred at 70 ºC for 1 h. When the reaction finishes, water is added, the mixture is extracted with THF and dried over Na_2SO_4. Products **4** are characterized by GC-MS.

Figures

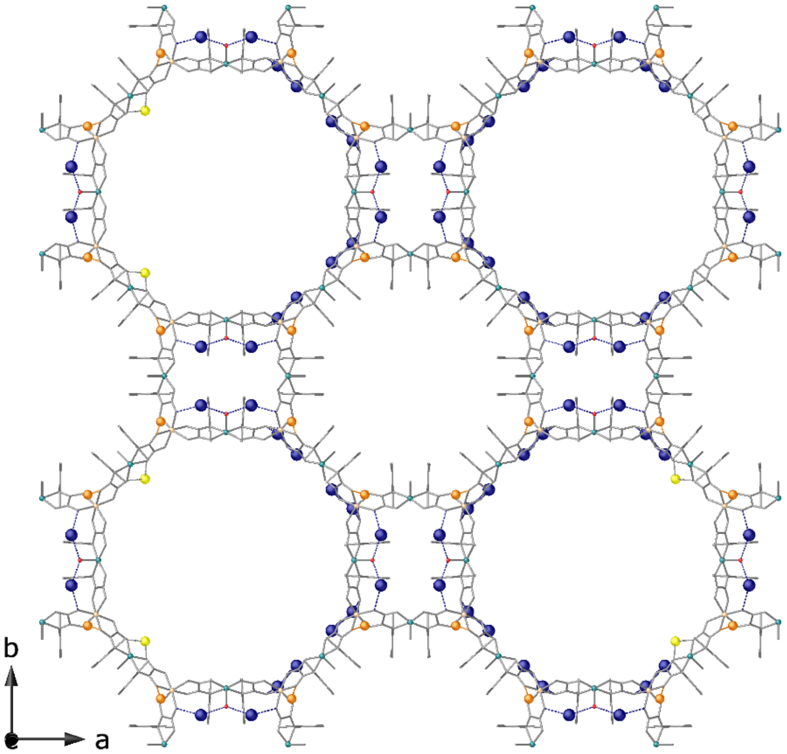

Figure S1. View along *c* crystallographic axis of **$Fe^{3+}Ag^0{}_2$@MOF** crystal structure showing randomly distribution of Fe^{3+} (yellow spheres) and $Ag^0{}_2$ dimers (blue spheres) inside octagonal hydrophilic pores together with $Ag^0{}_2$ dimers formed and blocked in the small square pores of the porous network. Na^+ alkali metal ions are represented by orange spheres. Copper and nickel atoms from the network are represented by cyan and orange spheres respectively, whereas organic ligands are depicted as grey sticks.

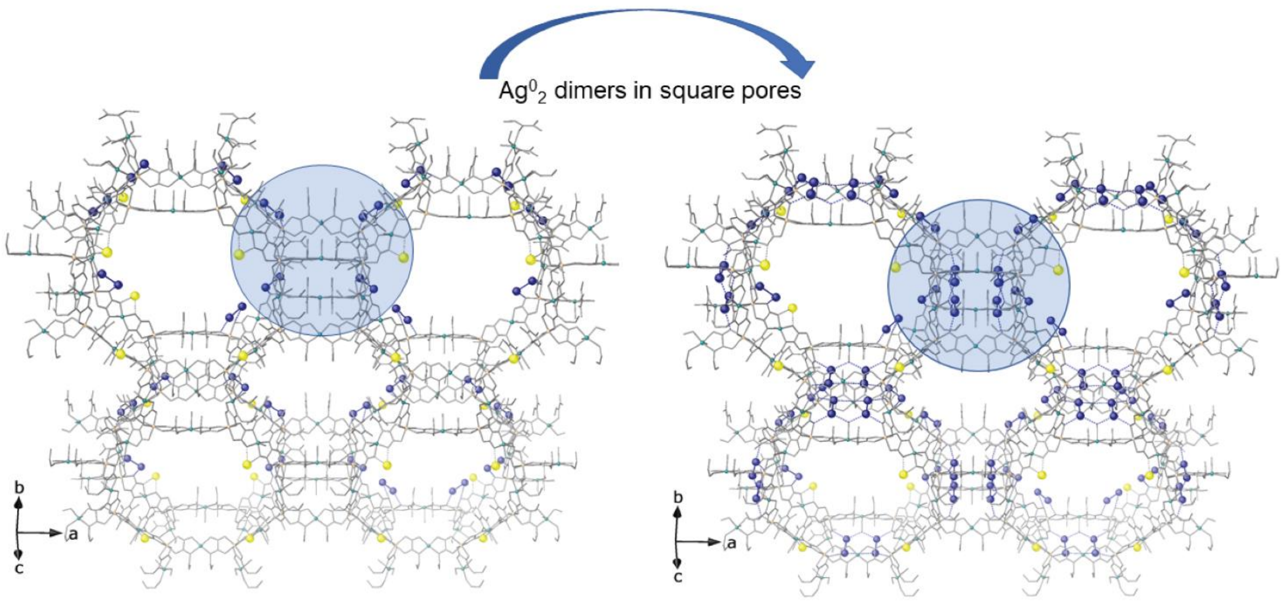

Figure S2. Perspective views of **$Fe^{3+}Ag^0_2$@MOF** crystal structure emphasising Ag^0_2 dimers (blue spheres) located in large hydrophilic pores (right) and small square pores (left). Fe^{3+} metal ions are depicted as yellow spheres. Copper and nickel atoms from the network are represented by cyan and orange spheres respectively, whereas organic ligands are depicted as grey sticks.

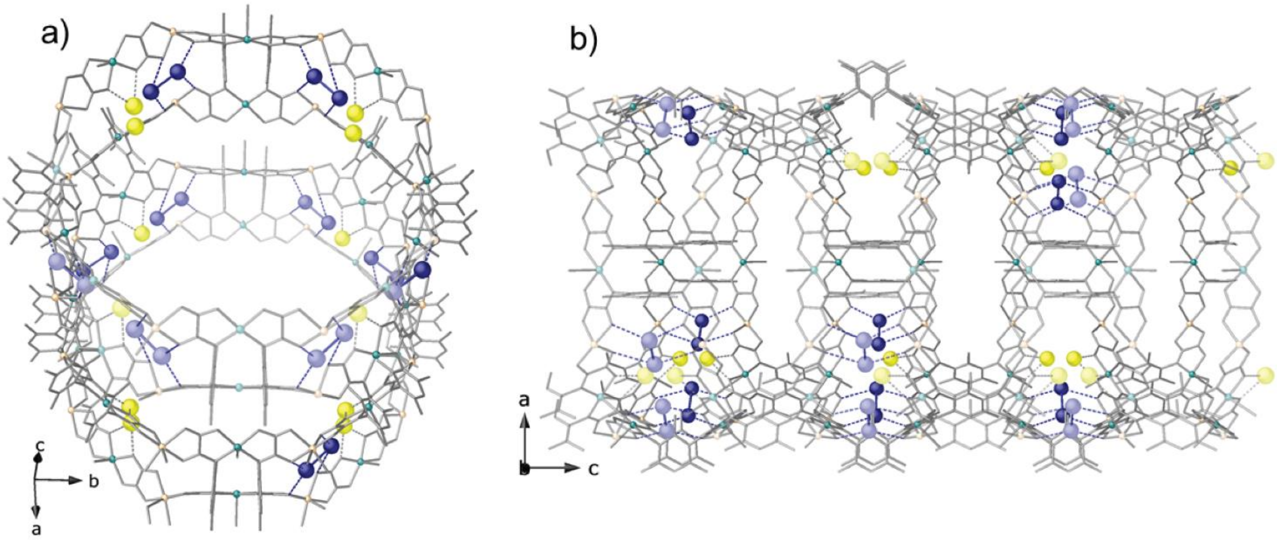

Figure S3. Top (a) and side view (b) of a single channel displaying decorated pore walls by uniform distribution of active Fe^{3+} isolated single metal atoms (yellow spheres) and Ag^0_2 subnanometric metal clusters (blue spheres). Copper and nickel atoms from the network are represented by cyan and orange spheres respectively, whereas organic ligands are depicted as grey sticks.

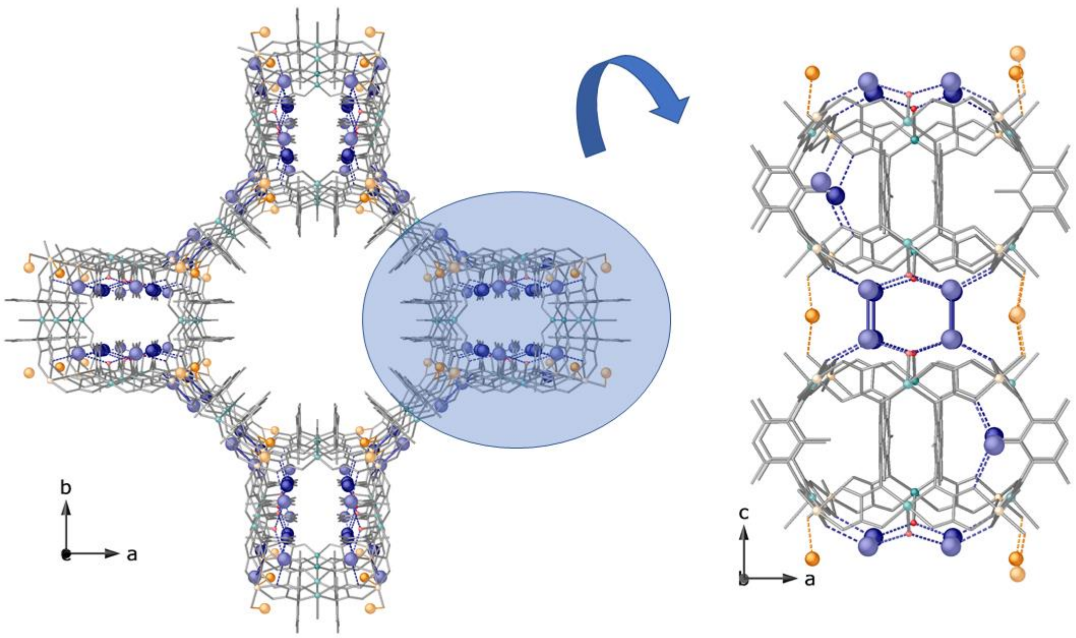

Figure S4. Perspective view along *c* and *b* axis of Ag^0_2 dimers (blue spheres) residing in the small square pores of the porous network stabilized by $Ag^0 \cdots O_{oxamate}$ interactions at 2.83(2) Å (blue dashed lines) and Na^+ alkali metal ions (orange spheres) connected to the walls of the network by means of non-covalent interactions. Copper and nickel atoms from the network are represented by cyan and orange spheres respectively, whereas organic ligands are depicted as grey sticks.

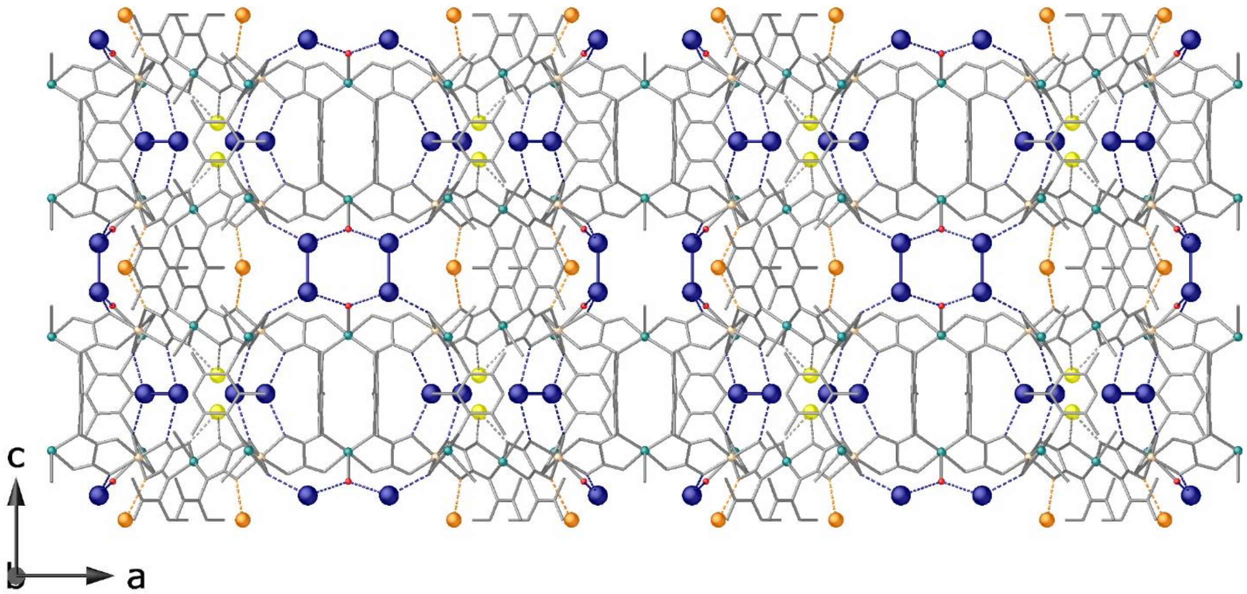

Figure S5. Perspective view of **$Fe^{3+}Ag^0_2$@MOF** crystal structures along *b* crystallographic axis clearly showing distribution of Ag^0_2 dimers (blue spheres) and Fe^{3+} metal ions (yellow spheres) together with hydrated alkali Na^+ cations (orange spheres), retained in preferential cationic sites, further contributing to the robustness of the final material. Copper and nickel atoms from the network are represented by cyan and orange spheres respectively, whereas organic ligands are depicted as grey sticks.

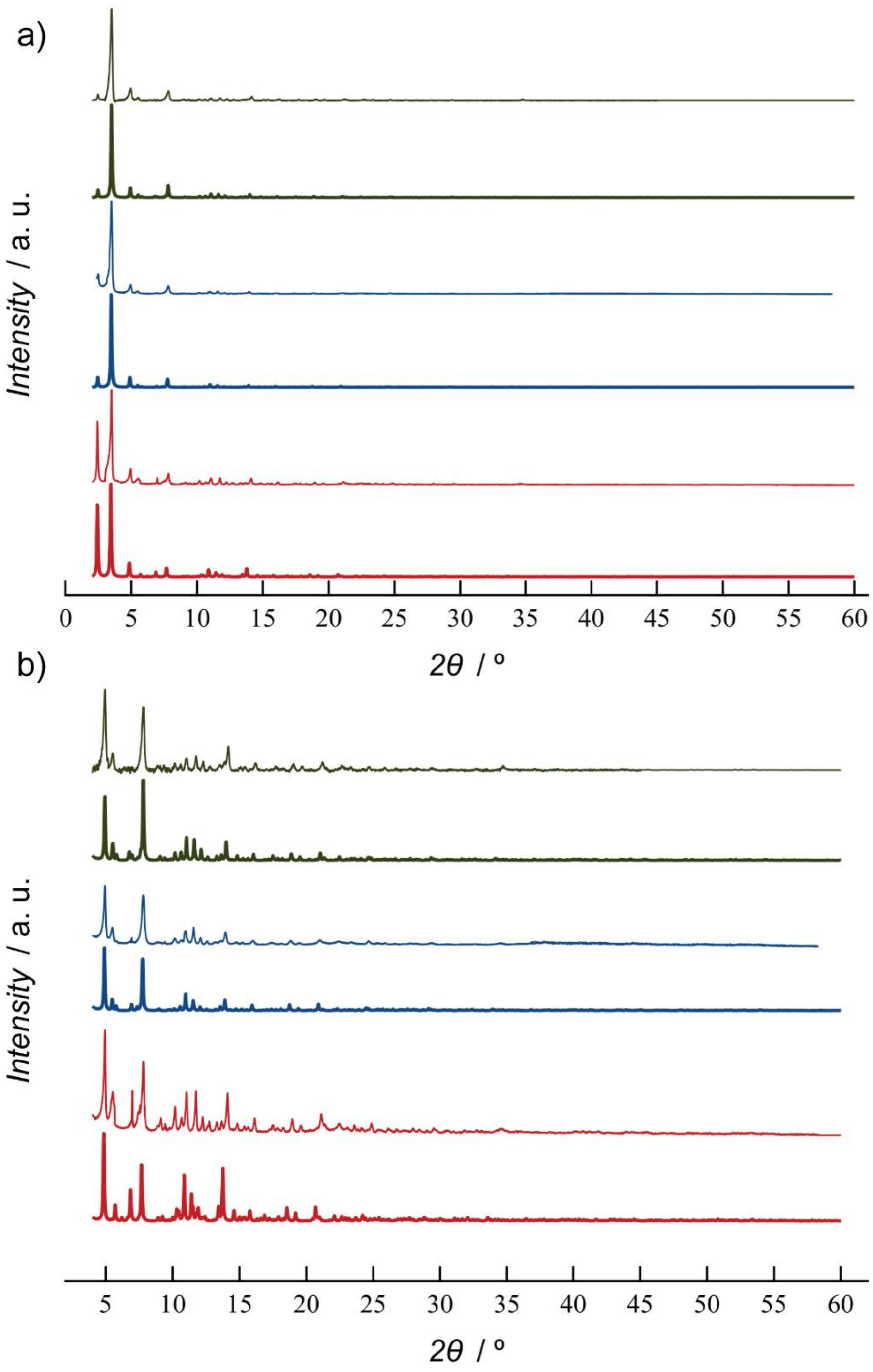

Figure S6. Theoretical (bold lines) and experimental (solid lines) PXRD pattern profiles of **Fe^{3+}@MOF** (red), **Ag^0_2@MOF** (blue) and **$Fe^{3+}Ag^0_2$@MOF** (green) in the 2*θ* range 2–60° (a) and 4–60° (b) for the sake of clarity.

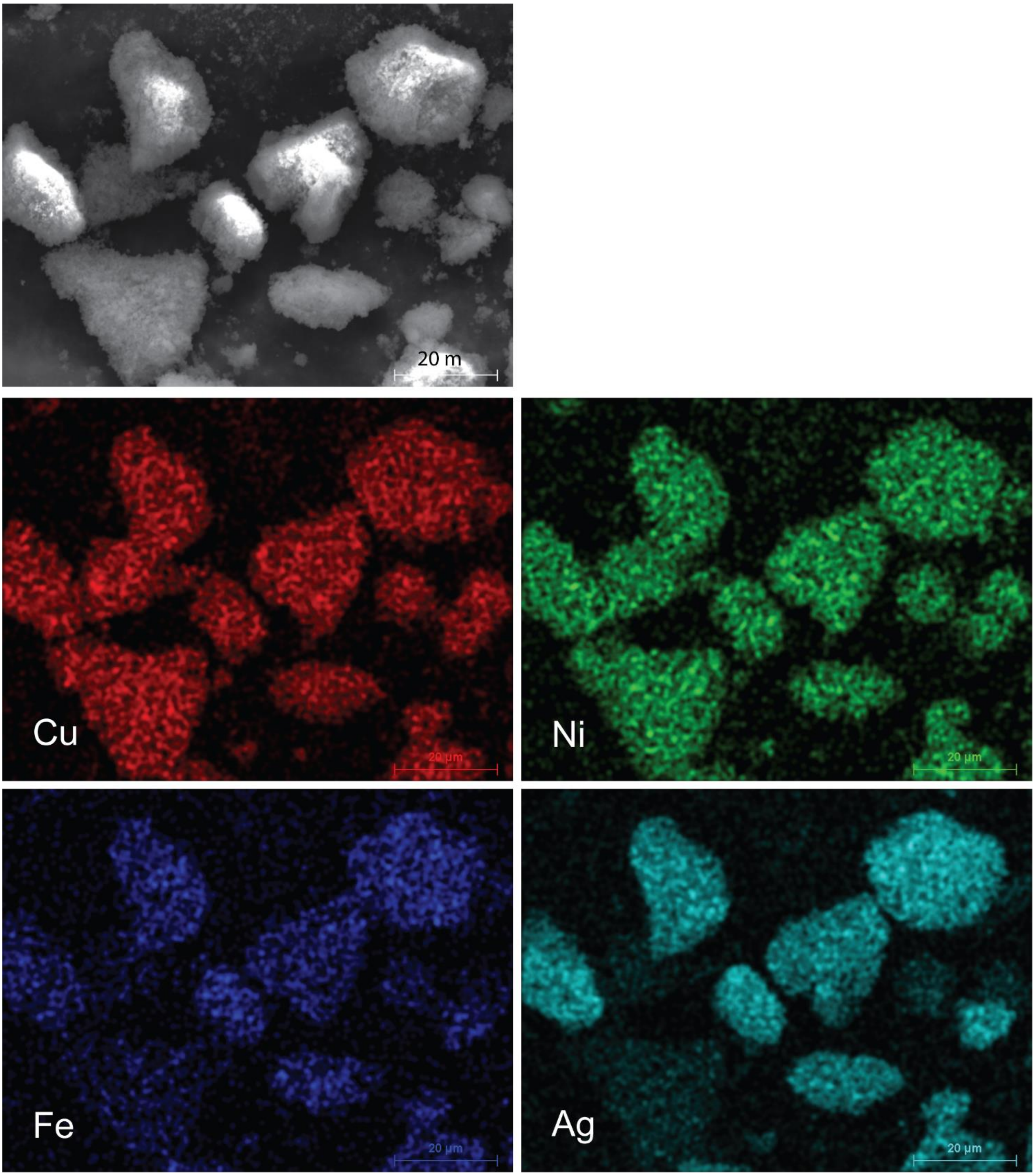

Figure S7. Backscattered SEM image of **$Fe^{3+}Ag^{0}_{2}$@MOF** and the corresponding EDX elemental mapping for Cu (red), Ni (green) Fe (blue) and Ag (light blue) elements. The backscattering detector highlights the MOF particles as brighter areas due to crystalline MOF structure and to the presence of heavier atoms in the MOF than in the polymer matrix.

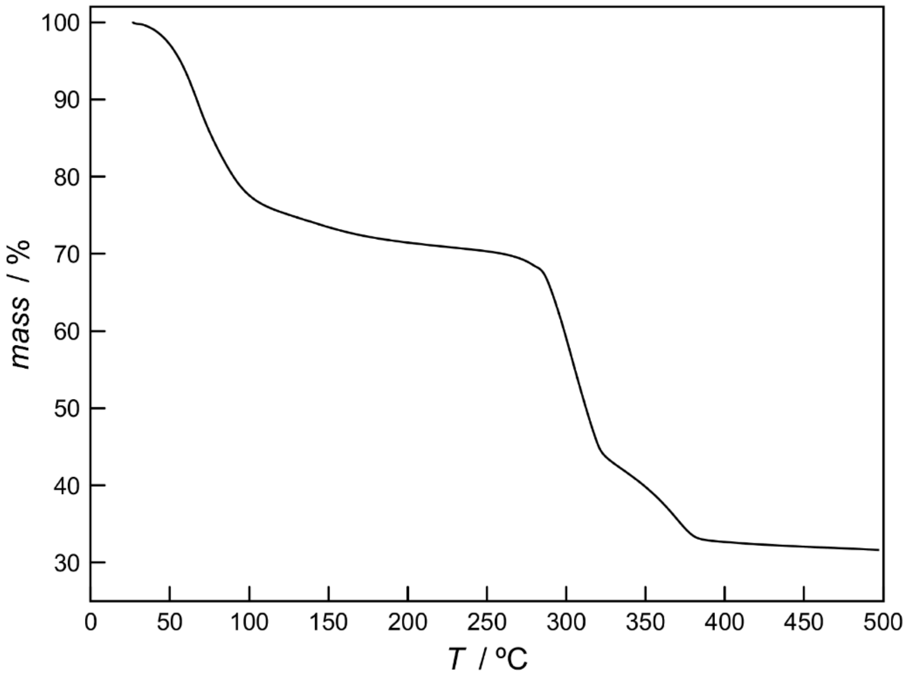

Figure S8. Thermo-Gravimetric Analyses (TGA) of **$Fe^{3+}Ag^{0}_{2}$@MOF** under a dry N_2 atmosphere.

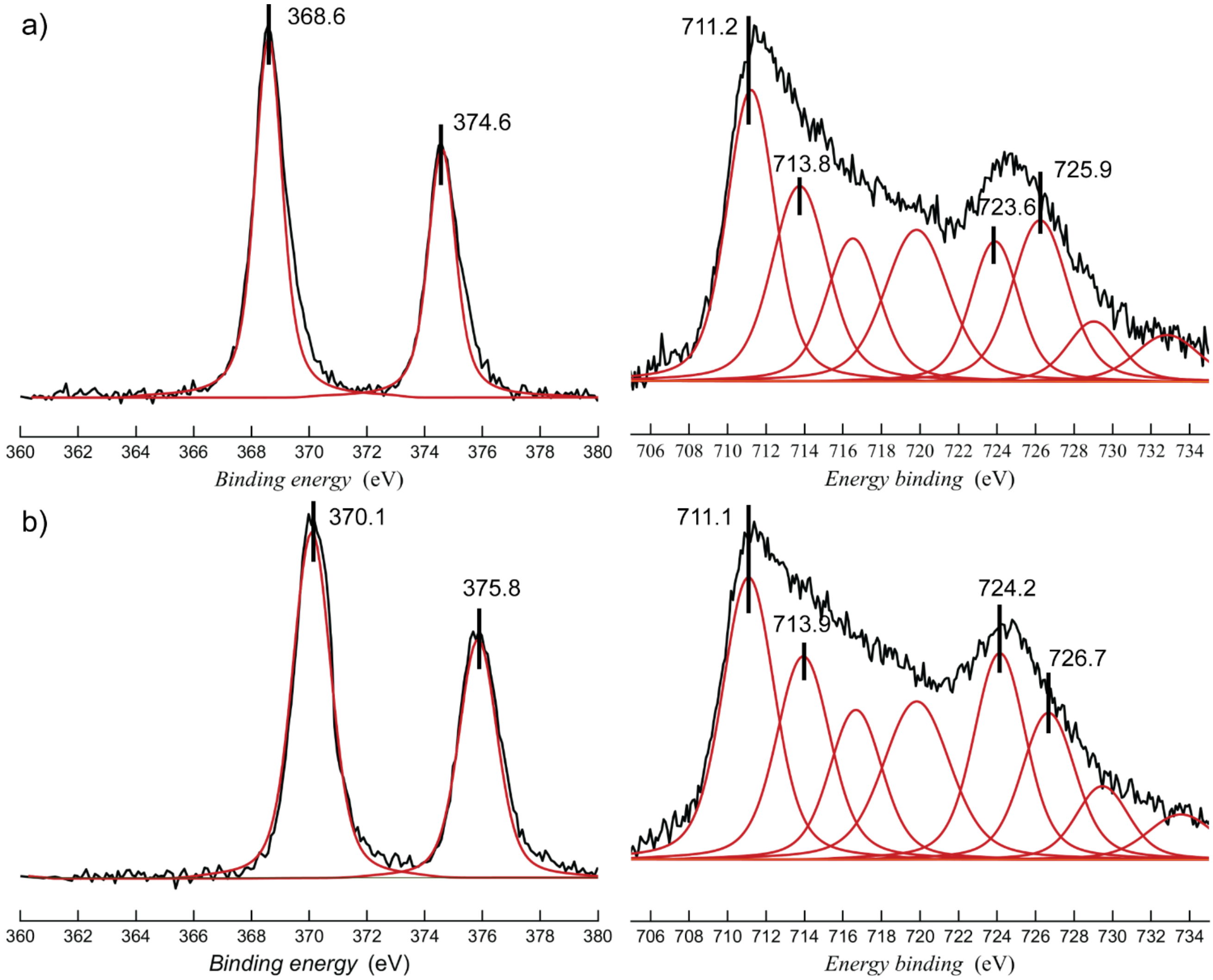

Figure S9. X–ray photoelectron spectroscopy (XPS) of **$Fe^{3+}Ag^{+}$@MOF** (a) and **$Fe^{3+}Ag^{0}_{2}$@MOF** (b) showing the deconvoluted signals of Ag $3d_{5/2}$ and Ag $3d_{3/2}$ (left) and Fe $2p_{3/2}$ and Fe $2p_{1/2}$ (right).

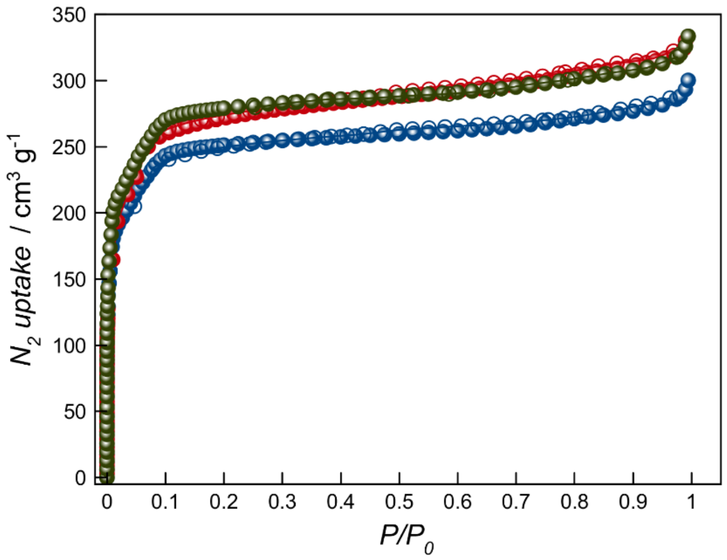

Figure S10. N_2 sorption (filled circles) and desorption (empty circles) isotherms for the activated compounds **Fe^{3+}@MOF** (blue), **Ag^0_2@MOF** (red) and **$Fe^{3+}Ag^0_2$@MOF** (green) at 77 K.

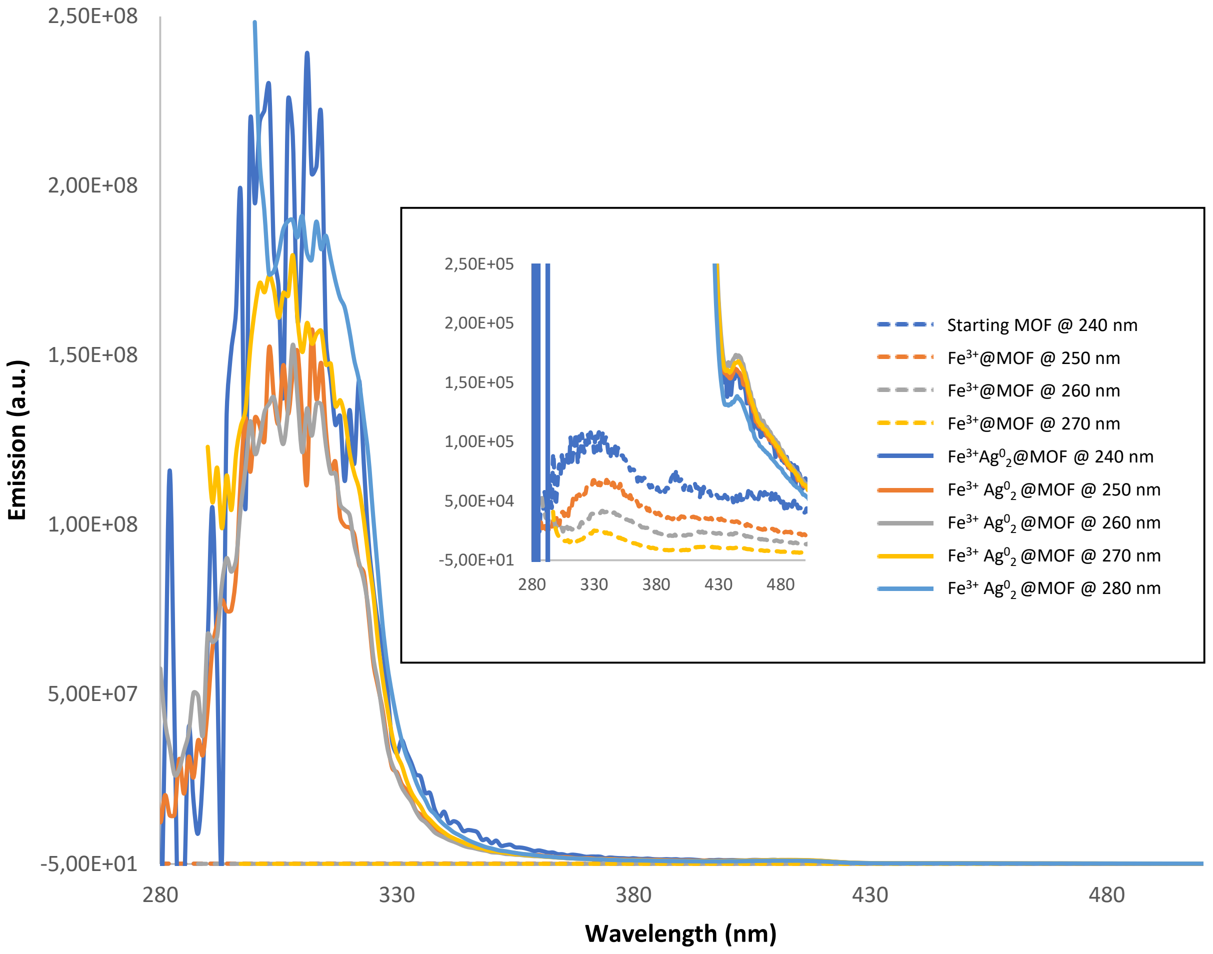

Figure S11. Fluorescence measurements in the UV-visible wavelength range for $\mathbf{Fe^{3+}@MOF}$ and $\mathbf{Fe^{3+}Ag^0_2@MOF}$ after irradiation at the indicated wavelengths, showing the expected fluorescence bands for Ag_2 at ~300 nm. These emission bands were not observed for $\mathbf{Fe^{3+}@MOF}$ (see inset), notice the three orders of magnitude increase in the emission of $\mathbf{Fe^{3+}Ag^0_2@MOF}$.

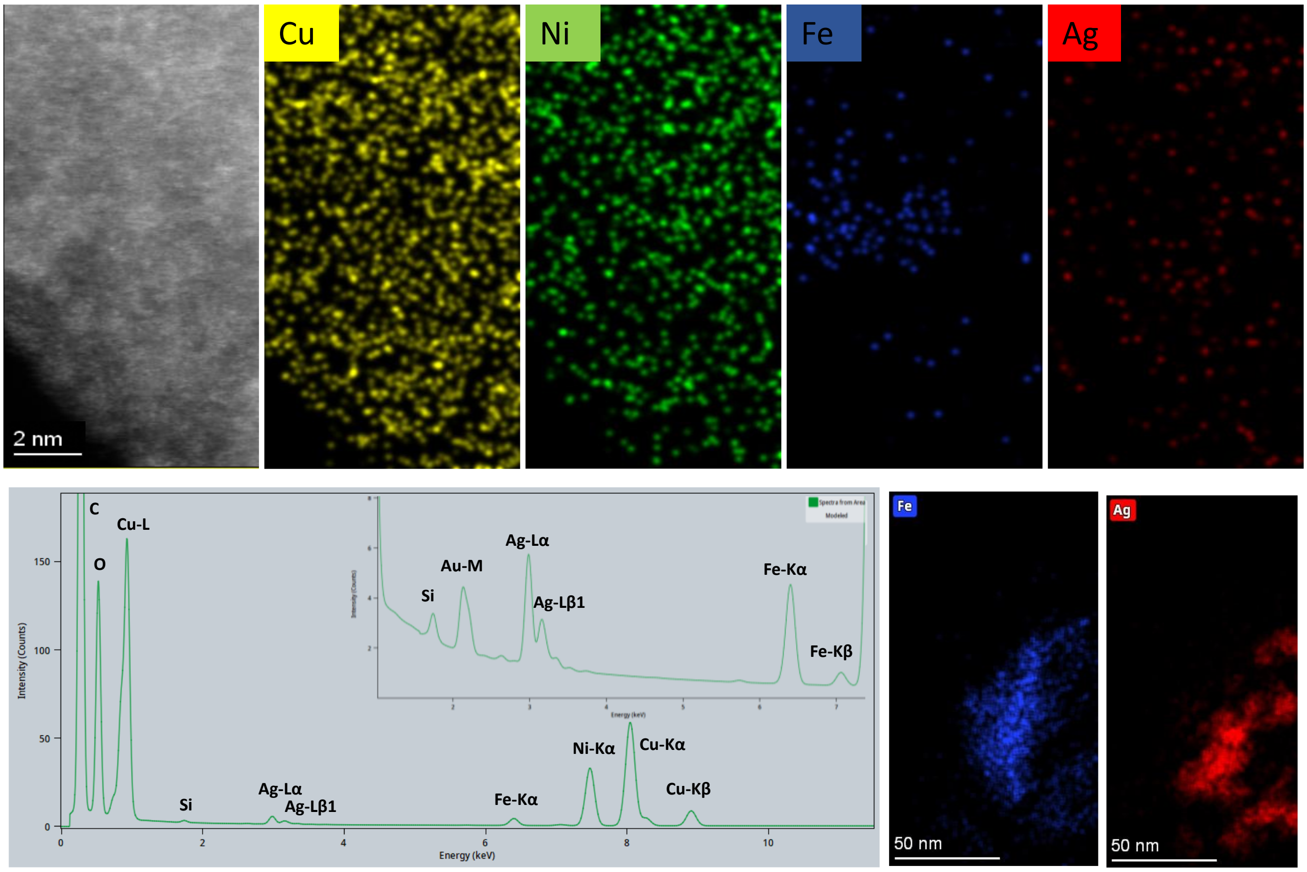

Figure S12. Representative HAADF-STEM image of **$Fe^{3+}Ag^0_2$@MOF** (top) with the corresponding elemental mapping and EDX spectrum (bottom left), and two additional mappings at lower magnification of Fe and Ag (bottom right).

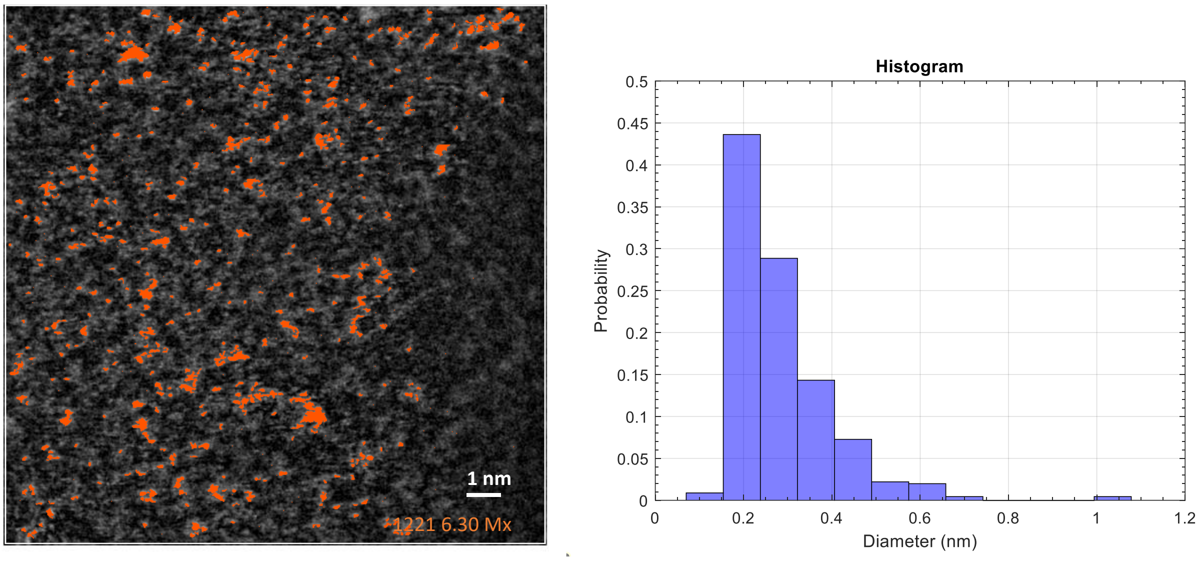

Figure S13. Denoised and background removed HAADF-STEM image of $\mathbf{Fe^{3+}Ag^{0}{}_{2}@MOF}$ with the Ag(0) aggregations shown in orange (left), and the corresponding histogram (right).

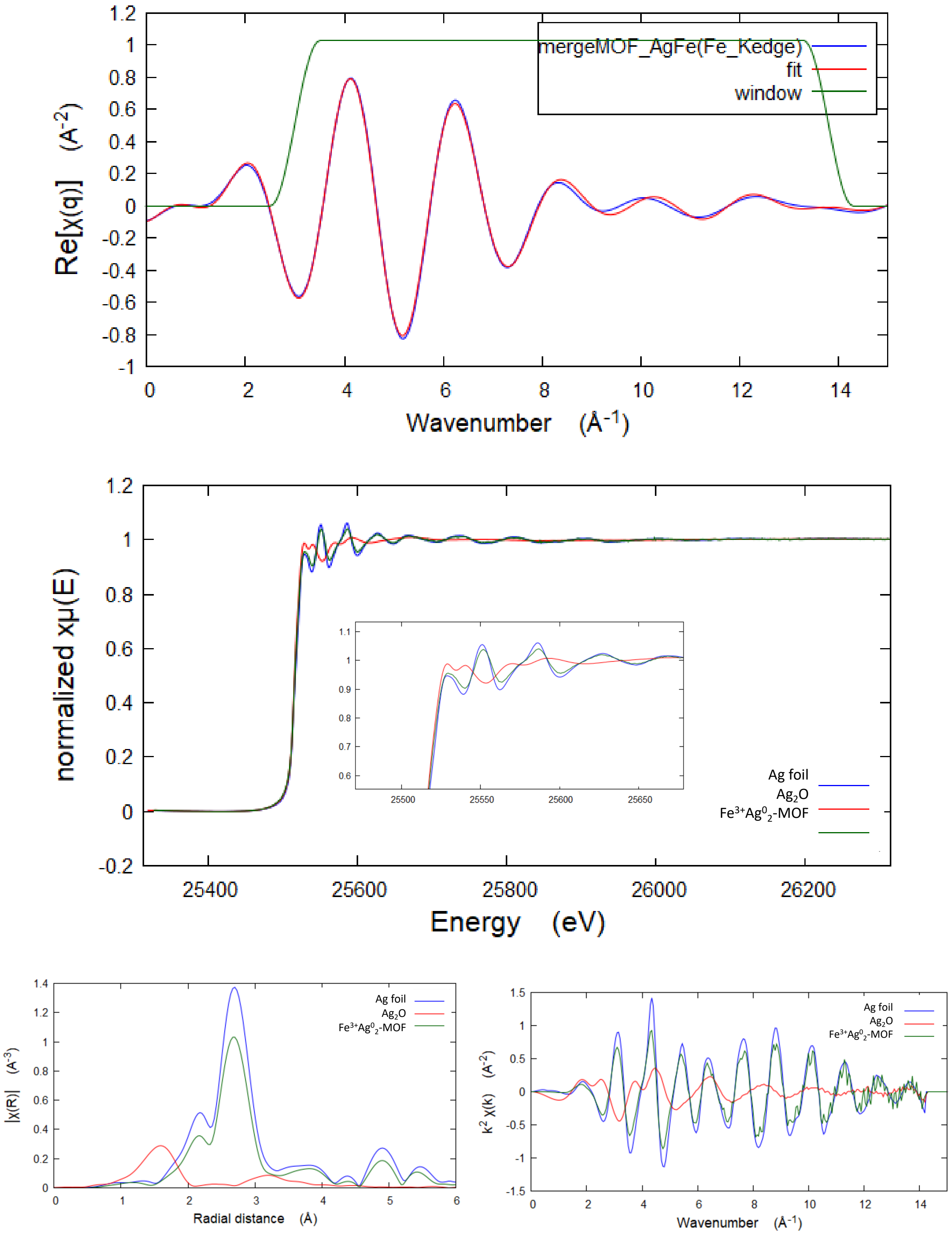

Figure S14. Extended X-ray absorption fine structure (EXAFS, top) spectrum of the k-weighted Fe-K edge first shell of **$Fe^{3+}Ag^{0}{}_{2}$@MOF**, fitting against wavenumber, and radial distance in the q-space; Ag K-edge X-ray absorption near-edge structure (XANES, middle); and EXAFS (bottom left) spectra and the Fourier transformed EXAFS (bottom right) of the **$Fe^{3+}Ag^{0}{}_{2}$@MOF** (green lines), compared to Ag foil (blue lines) and Ag_2O (red lines) as standard samples.

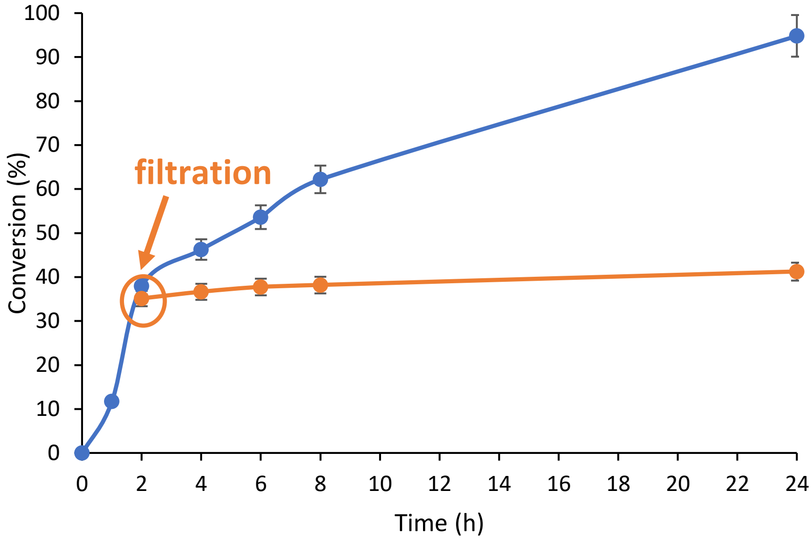

Figure S15. Hot filtration test for the synthesis of vinyl sulfone **4a** from styrene **1a** and phenyl sulfone **2** catalyzed by **$Fe^{3+}Ag^{0}_{2}$@MOF**, after adding the insoluble $K_2S_2O_8$ to the filtrates. The experiments were performed by duplicate. Error bars account for a 5% uncertainty.

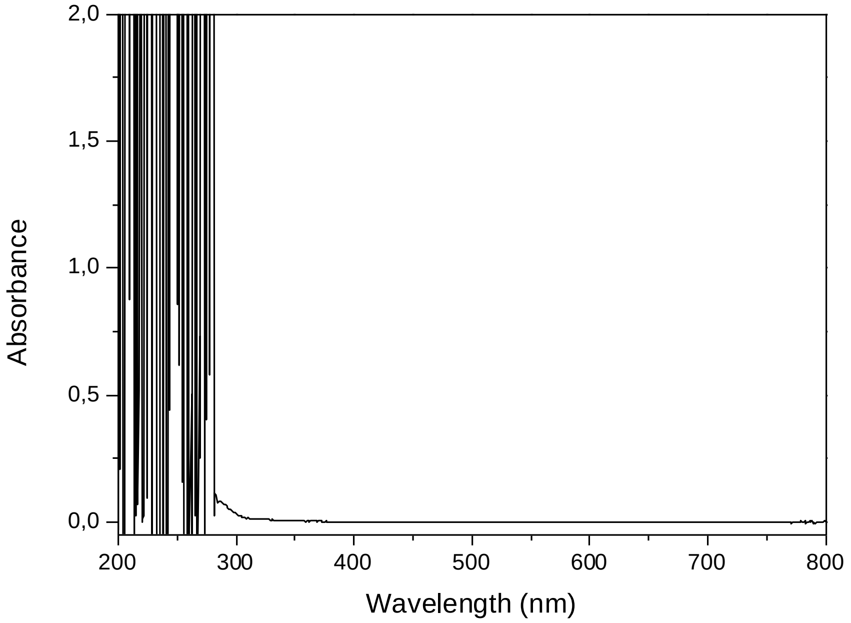

Figure S16. UV-vis absorption spectrum of the reaction mixture during the synthesis of vinyl sulfone **4a** from styrene **1a** and phenyl sulfone **2** with $AgNO_3$ and TEMPO as catalysts, in solution, do not showing the presence of any plasmonic Ag nanoparticles.

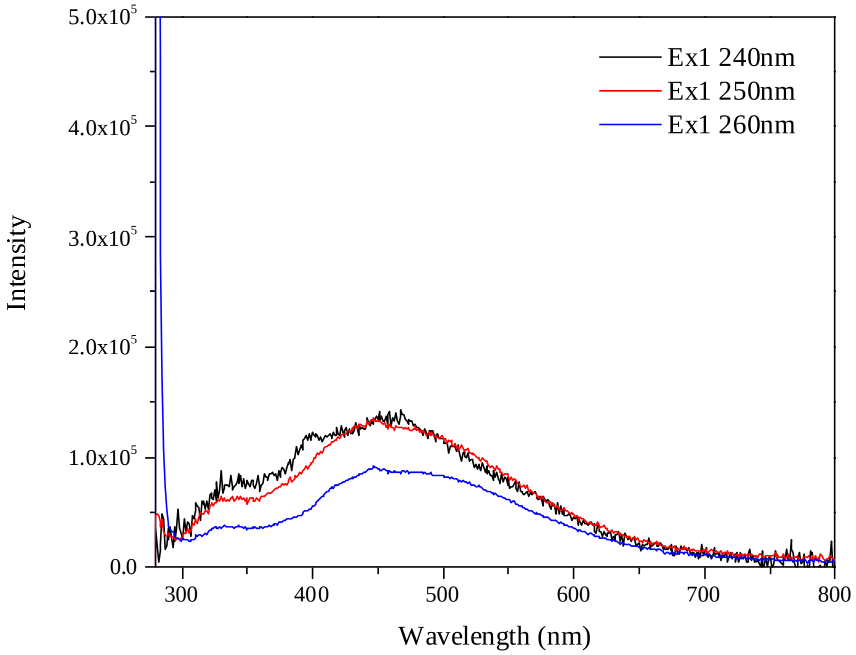

Figure S17. UV-vis emission spectrophotometry measurements of the reaction mixture during the synthesis of vinyl sulfone **4a** from styrene **1a** and phenyl sulfone **2** with $AgNO_3$ and TEMPO as catalysts, in solution, after irradiation at the indicated wavelengths. The presence of new fluorescence bands between 300 and 500 nm when irradiating the reaction mixture at 240-260 nm, is compatible with the formation in solution of Ag_{2-10} species.

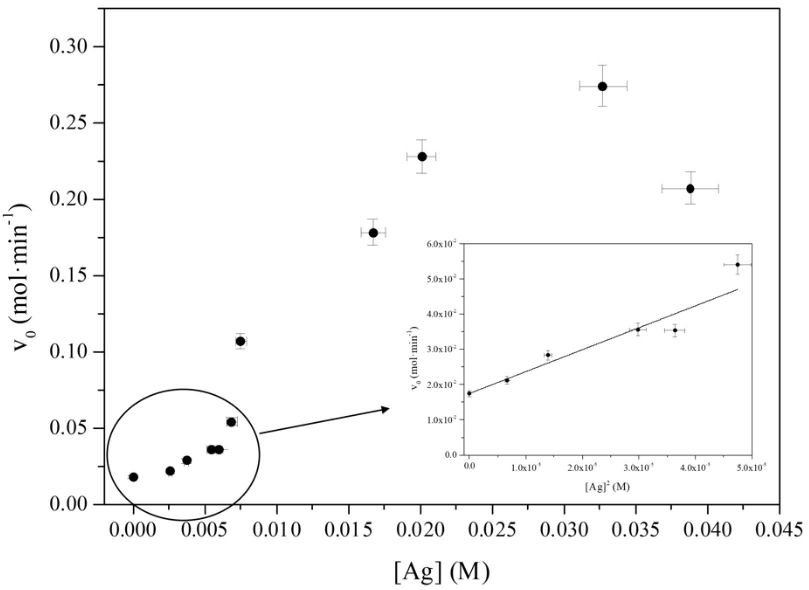

Figure S18. Kinetic plot of the initial rates during the oxidative cross-coupling reaction of styrene **1a** with phenyl sulfone **2** *vs* [Ag], under the conditions described in Table 1 (with $AgNO_3$ catalyst). All points were taken in individual experiments by triplicate. The inset shows the corresponding initial rates *vs* $[Ag]^2$. Error bars account for a 5% uncertainty. Lines are a guide to the eye.

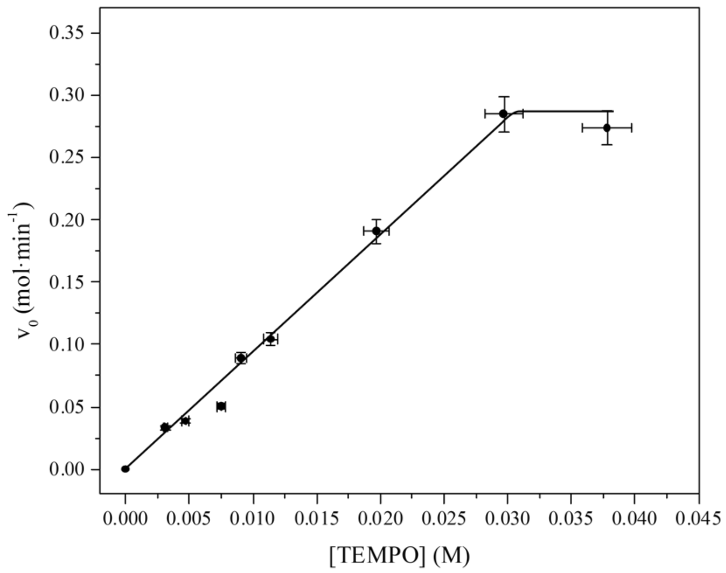

Figure S19. Kinetic plot initial rate *vs* [TEMPO] for the oxidative cross-coupling reaction of styrene **1a** with phenyl sulfone **2** under the conditions described in Table 1. All points were taken in individual experiments by duplicate. Error bars account for a 5% uncertainty. The line is a guide to the eye.

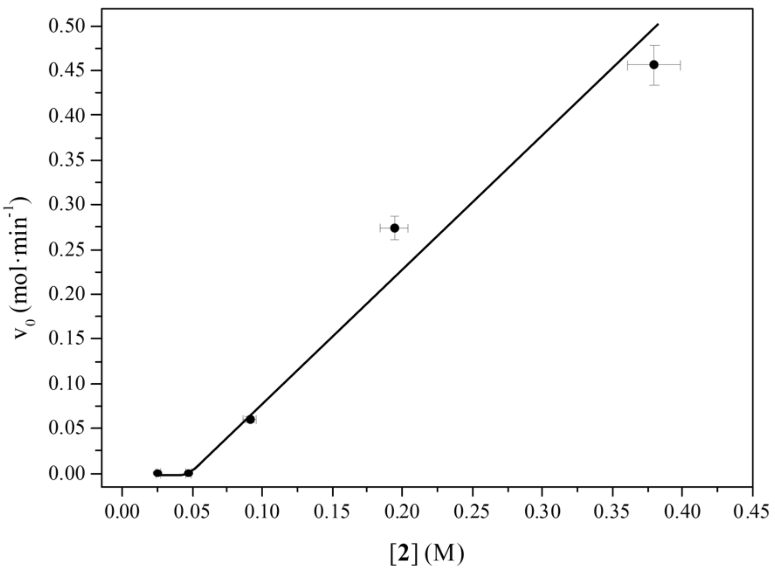

Figure S20. Kinetic plot initial rate *vs* [**2**] for the oxidative cross-coupling reaction of styrene **1a** with phenyl sulfone **2** under the conditions described in Table 1. All points were taken in individual experiments by duplicate. Error bars account for a 5% uncertainty. The line is a guide to the eye.

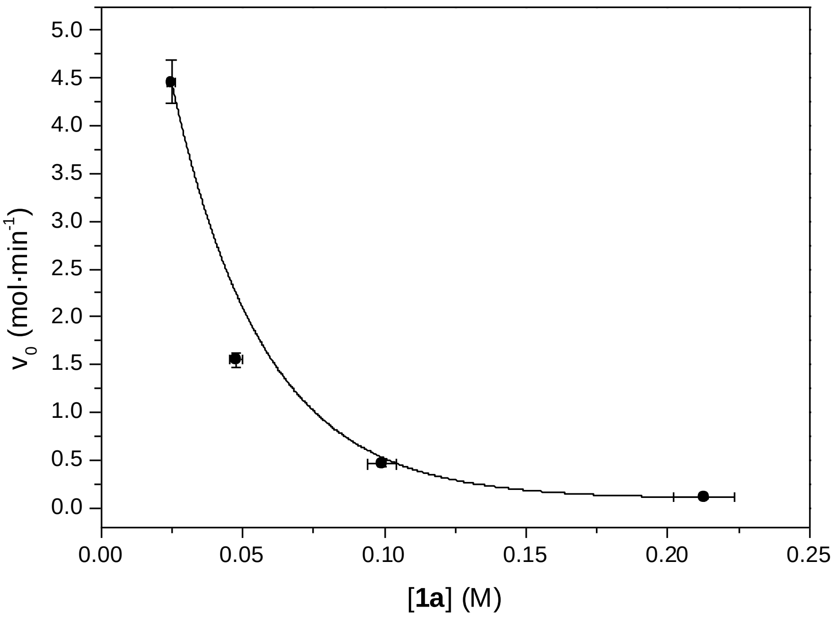

Figure S21. Kinetic plot initial rate *vs* [**1a**] for the oxidative cross-coupling reaction of styrene **1a** with phenyl sulfone **2** under the conditions described in Table 1. All points were taken in individual experiments by duplicate. Error bars account for a 5% uncertainty. The line is a guide to the eye.

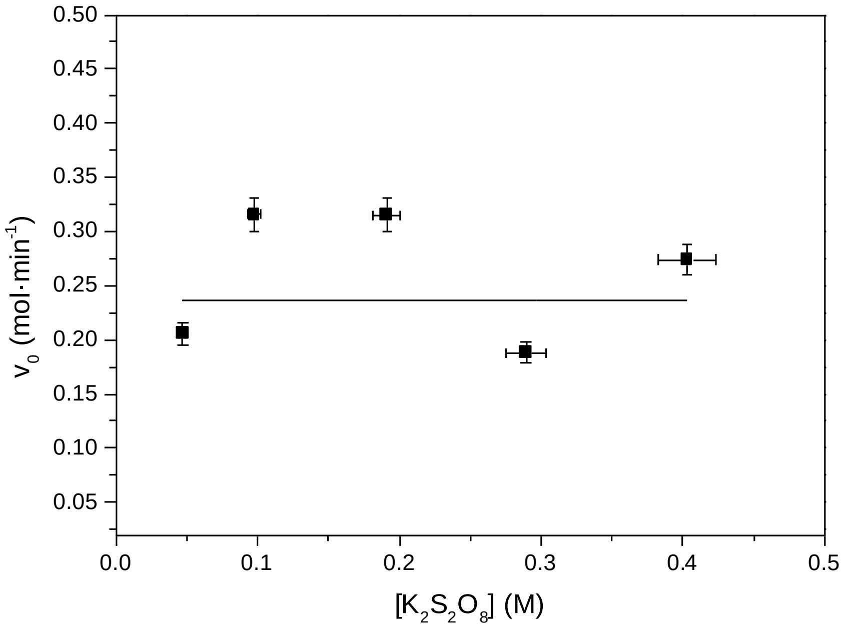

Figure S22. Kinetic plot initial rate *vs* [$K_2S_2O_8$] for the oxidative cross-coupling reaction of styrene **1a** with phenyl sulfone **2** under the conditions described in Table 1. All points were taken in individual experiments by duplicate. Error bars account for a 5% uncertainty. The line is a guide to the eye.

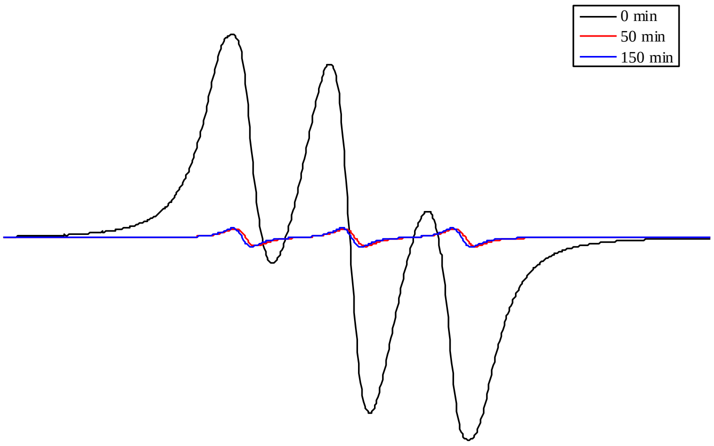

Figure S23. Electronic paramagnetic resonance (EPR) spectra of TEMPO during the oxidative cross-coupling reaction of styrene **1a** with phenyl sulfone **2** catalyzed by $AgNO_3$, without $K_2S_2O_8$, in order to prove if TEMPO is consumed during the first steps of the reaction.

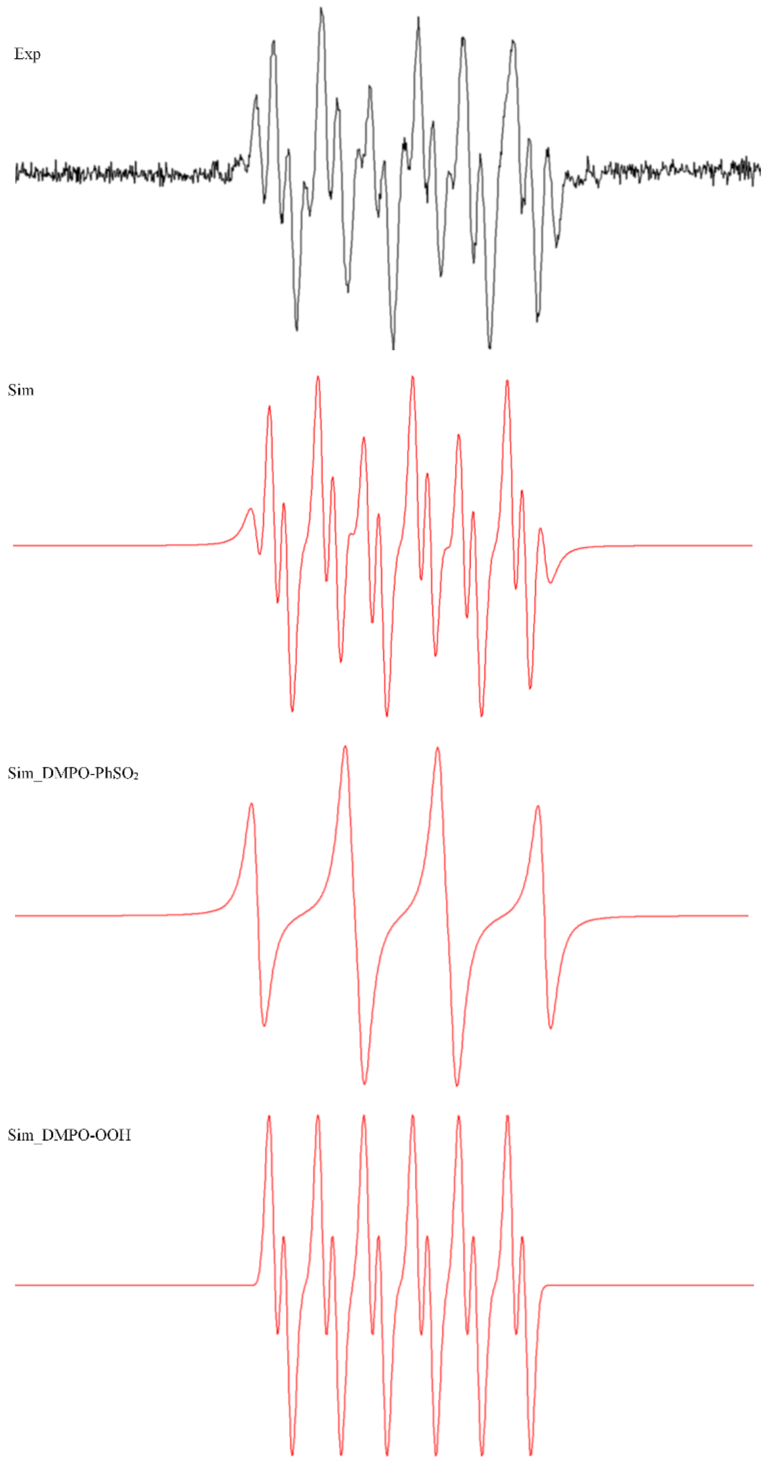

Figure S24. Electronic paramagnetic resonance (EPR) spectra of experiments (Exp) and simulation (Sim) for the oxidative cross-coupling reaction of styrene **1a** with phenyl sulfone **2** catalyzed by $AgNO_3$. The hyperfine parameters of the DMPO adduct radical are: DMPO-$PhSO_2$, A_N = 12.45 G, $A_{H\beta}$ = 13.68 G; DMPO-OOH, A_N = 12.73 G, $A_{H\beta}$ = 6.56 G, $A_{H\beta}$ = 1.74 G.

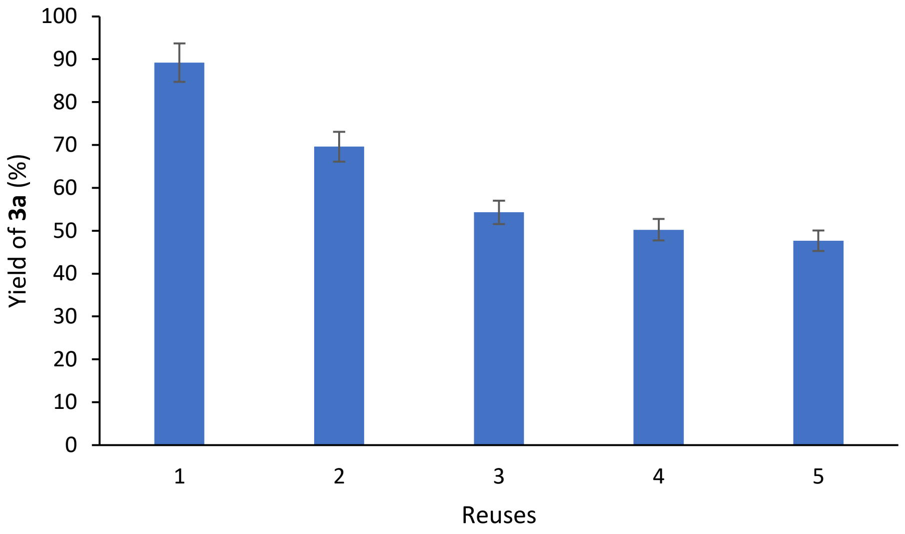

Figure S25. Reuses of the **$Fe^{3+}Ag^{0}_{2}$@MOF** catalyst for the synthesis of phenylacetylene **3a** from styrene **1a** through intermediate vinyl-sulfone **4a**, using K^{t}OBu in THF for the elimination step. All points were taken in individual experiments by duplicate. Error bars account for a 5% uncertainty.

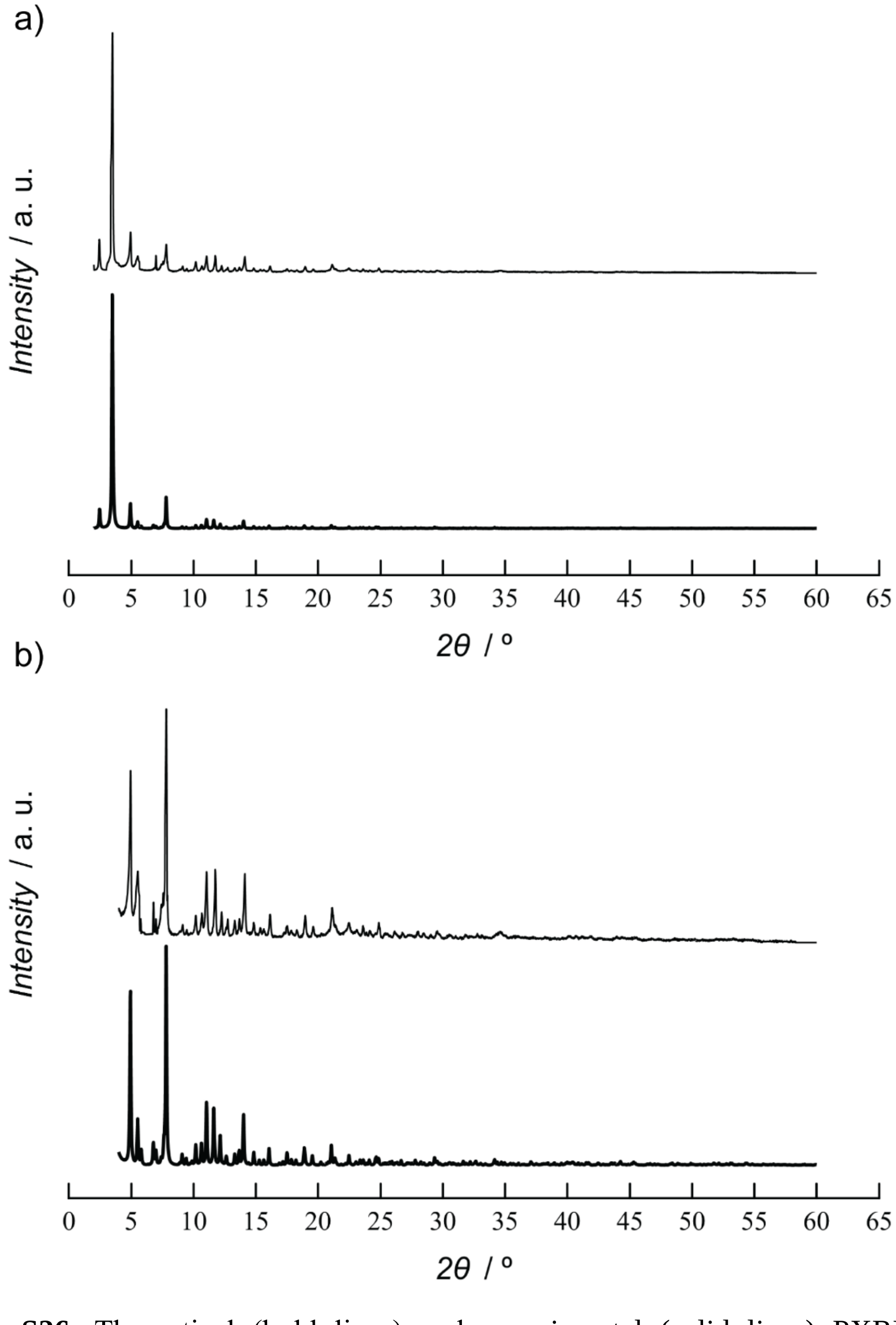

Figure S26. Theoretical (bold lines) and experimental (solid lines) PXRD pattern profiles of $\mathbf{Fe^{3+}Ag^{0}_{2}@MOF}$, after 5 catalytic cycles, in the 2θ range 2–60° (a) and 4–60° (b) for the sake of clarity.

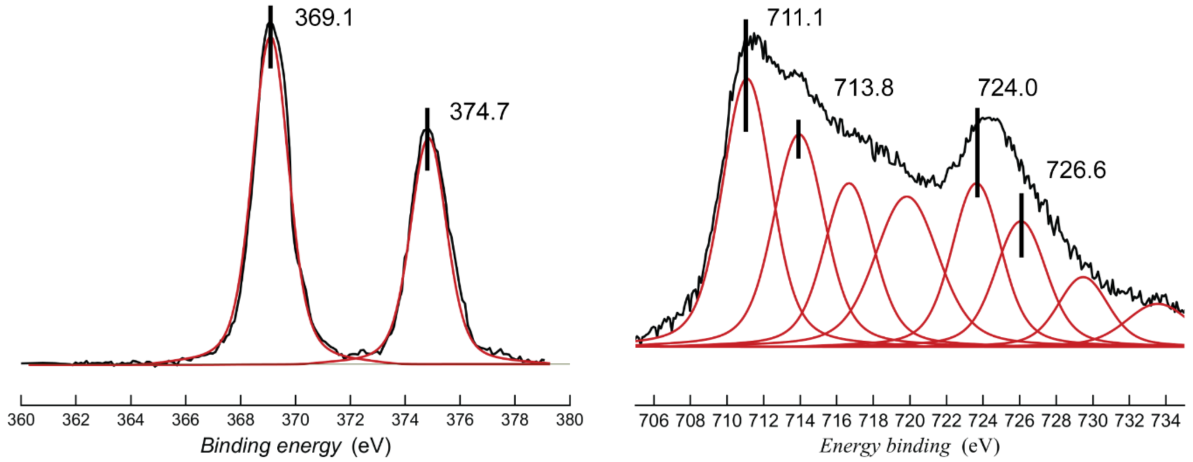

Figure S27. X–ray photoelectron spectroscopy (XPS) of **$Fe^{3+}Ag^{0}_{2}$@MOF**, after 5 catalytic cycles, showing the deconvoluted signals of Ag $3d_{5/2}$ and Ag $3d_{3/2}$ (left) and Fe $2p_{3/2}$ and Fe $2p_{1/2}$ (right).

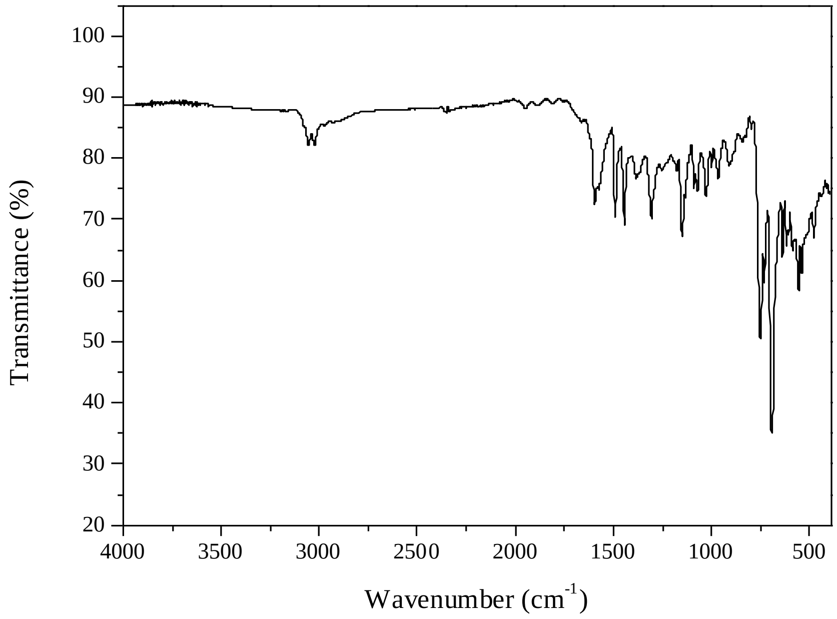

Figure S28. Fourier transformed infrared (FT-IR) spectrum of the product mixture obtained after the one-pot oxidative cross-coupling reaction of styrene **1a** with phenyl sulfone **2** and in-situ base treatment.

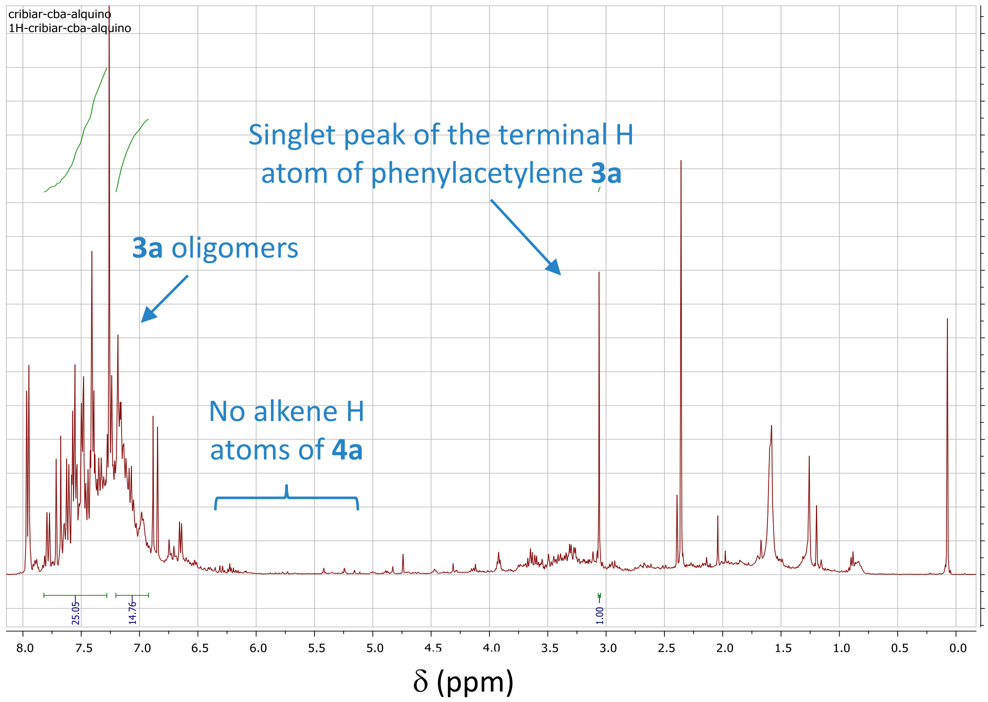

Figure S29. ^{1}H nuclear magnetic resonance (^{1}H-NMR) of the product mixture obtained after the one-pot oxidative cross-coupling reaction of styrene **1a** with phenyl sulfone **2** and in-situ base treatment.

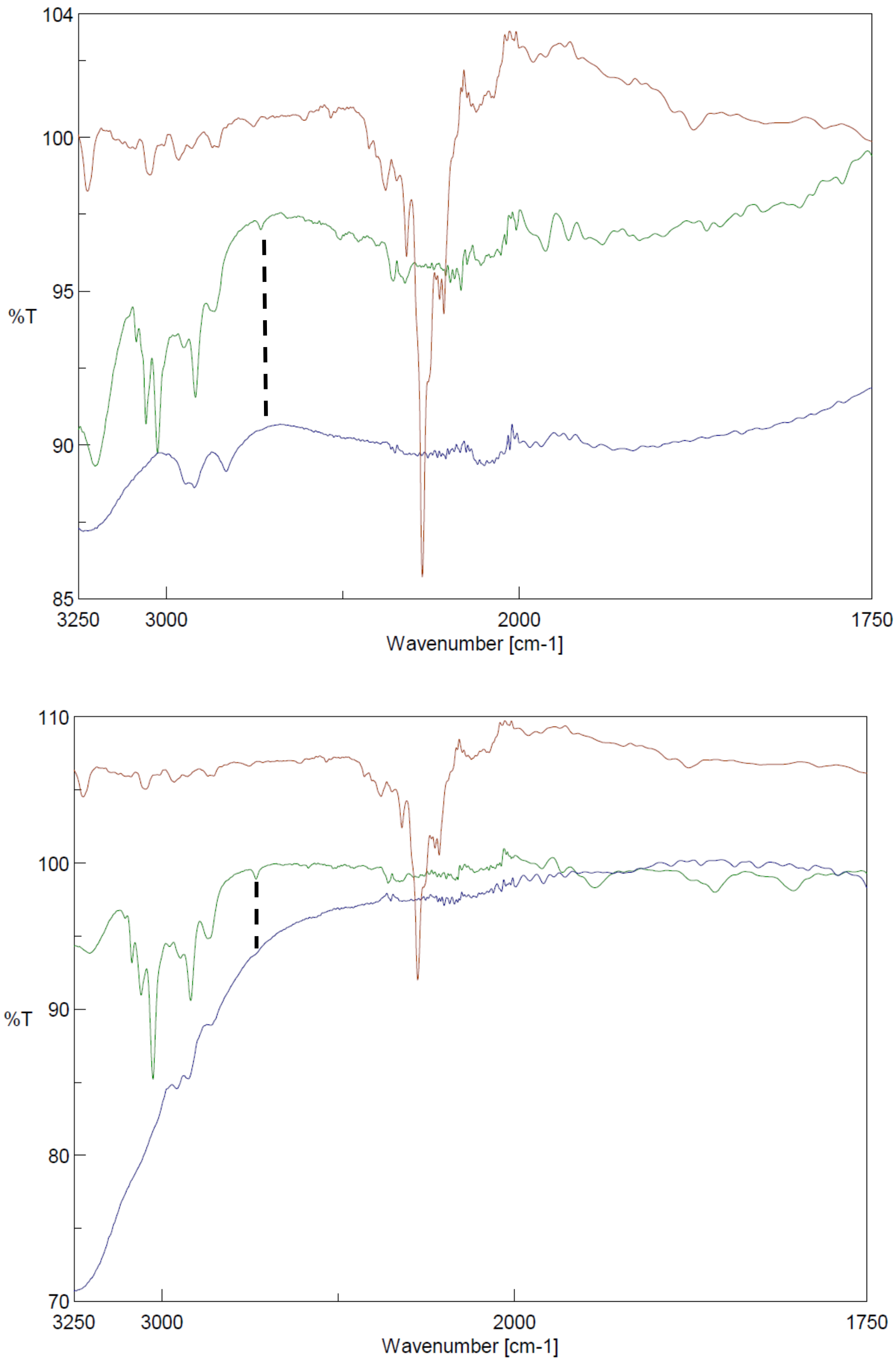

Figure S30. Fourier transformed infrared (FT-IR) part of the spectra (between 1700 and 3250 cm^{-1}) of the **$Fe^{3+}Ag^{0}_{2}$@MOF** solid (top) and the **Fe^{3+}@MOF** solid (bottom) after the oxidative cross-coupling reaction of isotopically labeled styrene–d^8 (**1a–*d*^{8}**, >98% deuterium incorporation) with phenyl sulfone **2** and without $K_2S_2O_8$. Fresh MOF (blue line), spent MOF (green line), **1a–*d*^{8}** (red line). The line indicates the new O-D band (~2750 cm^{-1}).

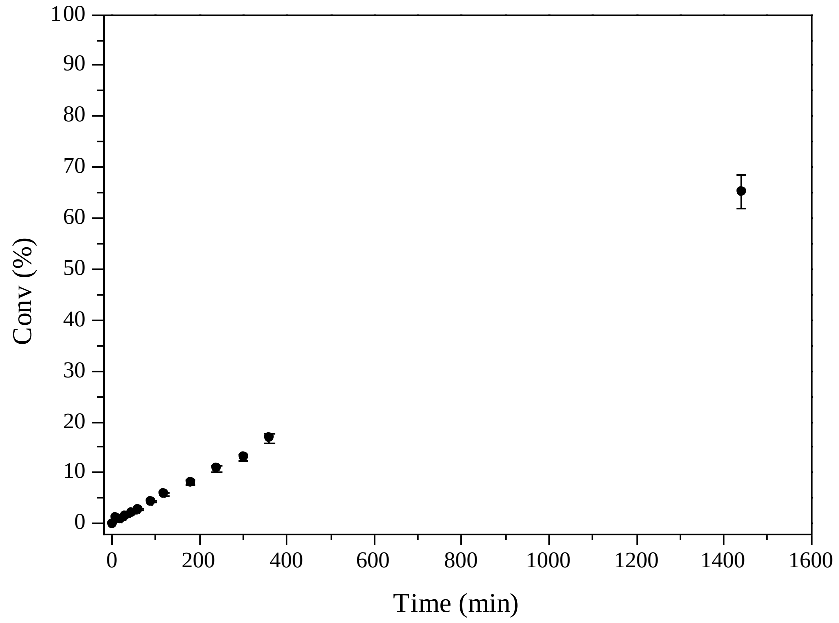

Figure S31. Kinetics for the one-pot oxidative cross-coupling reaction of styrene **1a** with phenyl sulfone **2** catalyzed by $AgNO_3$ in acetonitrile instead than in toluene, under the standard reaction conditions but without TEMPO. Error bars account for a 5% uncertainty.

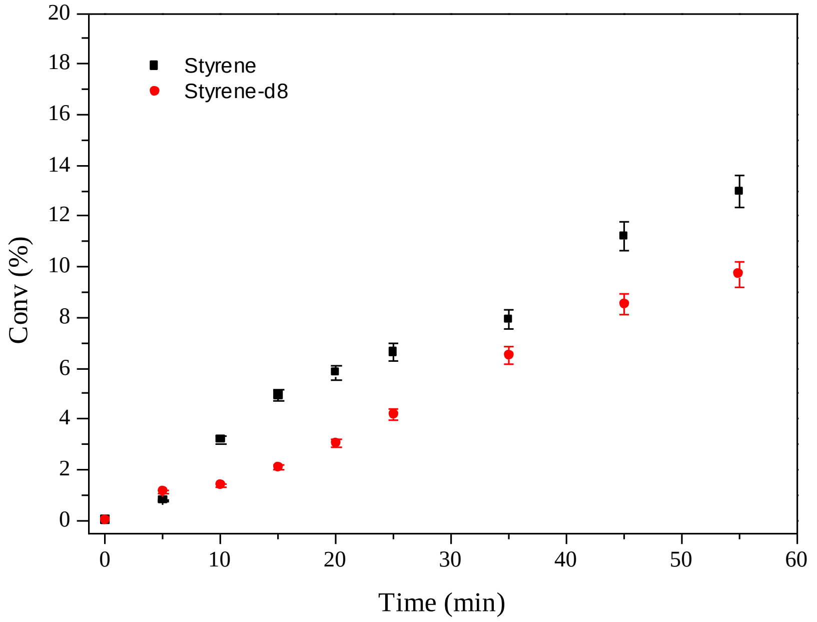

Figure S32. Kinetics for the one-pot oxidative cross-coupling reaction of styrene **1a** or styrene–d^8 (**1a–*d*⁸**, >98% deuterium incorporation) with phenyl sulfone **2** catalyzed by $AgNO_3$ under standard reaction conditions. Obtained values: y= 0.246x, R^2= 0.9912 for **1a** and y= 0.1778x, R^2 =0.9949 for **1a–d^8**. KIE= 1.3(9). Error bars account for a 5% uncertainty.

<u>**Tables.**</u>

Table S1. Selected data from the ICP–MS[a] and SEM/EDX[b] analyses for **$Fe^{3+}Ag^{0}_{2}$@MOF**.

**Metal**	*% mass*[a]	*Metal stoichiometry*[a]	*% mass*[b]	*Metal stoichiometry*[b]
**Cu**	9.678 (9.692)	6.01 (6.02)	9.81	6.09
**Ni**	5.953 (5.961)	4.00 (4.01)	6.01	4.04
**Fe**	0.949 (0.941)	0.67 (0.66)	0.91	0.64
**Ag**	9.435 (9.399)	3.45 (3.44)	9.59	3.51
**Na**	1.181 (1.168)	2.02 (2.00)	–	–

[a]Solid samples were digested with 0.5 mL of HNO_3 69% at 60°C for 4 hours followed by the addition of 0.5 mL of HCl 37% and digestion 80°C for 1 hour. Values in parentheses correspond to ICP-MS measurements for **$Fe^{3+}Ag^{0}_{2}$@MOF** after catalytic experiments. [b]SEM/EDX measurements were carried out on a solid policrystalline sample of **$Fe^{3+}Ag^{0}_{2}$@MOF**.

Table S2. Summary of Crystallographic Data for **$Fe^{3+}Ag^{0}_{2}$@MOF.**

Compound	**$Fe^{3+}Ag^{0}_{2}$@MOF**
Formula	$C_{78}H_{186}Ag_{3.44}Fe_{0.66}Na_{2}Cu_{6}Ni_{4}N_{12}O_{99}$
M (g mol^{-1})	3946.37
λ (Å)	0.71073
Crystal system	tetragonal
Space group	*P*4/*mmm*
a (Å)	35.8023(16)
c (Å)	15.2143(9)
V (Å^3)	19502(2)
Z	4
ρ_{calc} (g cm^{-3})	1.344
μ (mm^{-1})	1.492
T (K)	150
θ range for data collection (°)	1.137- 20.848
Completeness to θ = 25.0	94%
Measured reflections	35590
Unique reflections (Rint)	5756(0.1219)
Observed reflections [$I > 2\sigma(I)$]	2992
Goof	0.951
R^a [$I > 2\sigma(I)$] (all data)	0.0872 (0.1469)
wR^b [$I > 2\sigma(I)$] (all data)	0.2363 (0.2619)
CCDC	2157534

[a] $R = \sum(|F_o| - |F_c|)/\sum|F_o|$. [b] $wR = [\sum w(|F_o| - |F_c|)^2/\sum w|F_o|^2]^{1/2}$.

Table S3. Fe K-edge EXAFS fitting results for $Fe^{3+}Ag^0_2$@MOF.

	**CN**	**R (Å)**	**ΔE0 (eV)**	**ss²**
Fe-O	2.63 ± 0.21	1.97	6.96 ± 1.07	0.011 ± 0.001

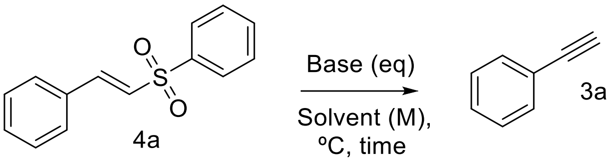

Table S4. Results for the synthesis of alkyne **3a** from vinyl sulfone **4a** under basic conditions. GC yields; average of, at least, two runs.[a] Some oligomers of **3a** were also found and are included in the yield.[b] Commercially available KO^{t}Bu in THF gives the same result than solid KO^{t}Bu dispersed in THF.

Entry	Base (equivalents)	Solvent (M)	Temperature (ºC)	Time (min)	3a (yield, %)[a]
1	K_2CO_3 (2)	THF (2)	70	60	<1
2	Cs_2CO_3 (2)				<1
3	K_3PO_4 (2)				<1
4	NaO^{t}Bu (2)				8
5[b]	KO^{t}Bu (2)				>99
6	KO^{t}Bu (1.5)				>99
7			40		93
8			25		80
9			0		6
10		THF (3)	25		85
11		THF (1)			62
12		THF (0.75)			20
12	NaO^{t}Bu (1.5)				2
14	KO^{t}Bu (1)	THF (3)	70		68
15	KO^{t}Bu (2)				>99
16				15	>99
17		Toluene (3)		60	<5
18		Acetonitrile (3)			<5

Characterization of isolated compounds.

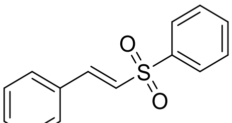

4a

(*E*)-(2-(phenylsulfonyl)vinyl)benzene (4a). Yield: 95.0 %. ^{1}H NMR (300 MHz, $CDCl_3$): δ = 7.97–7.94 (m, 2H), 7.69 (d, J = 15.4 Hz, 1H), 7.65–7.47 (m, 5H), 7.44–7.36 (m, 3H), 6.86 (d, J = 15.4 Hz, 1 H) ppm. ^{13}C NMR (75 MHz, $CDCl_3$): δ = 141.5, 139.8, 132.3, 131.4, 130.2, 128.3, 128.1, 127.6, 126.7, 126.3 ppm. IR: ν = 3062, 1611, 1575, 1447, 1305, 1177, 1141, 1083, 977, 857, 748, 690 cm^{-1}.

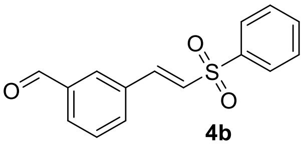

4b

(*E*)-3-(2-(phenylsulfonyl)vinyl)benzaldehyde (4b). Yield: 87.8 %. ^{1}H NMR (401 MHz, $CDCl_3$): δ = 10.01 (s, 1H), 8.05–7.92 (m, 3H), 7.90 (d, J = 7.6 Hz, 1H), 7.77–7.68 (m, 2H), 7.65–7.53 (m, 4H), 6.96 (d, J = 15.5 Hz, 1H) ppm. ^{13}C NMR (101 MHz, $CDCl_3$): δ = 191.4, 140.6, 140.4, 137.2, 134.2, 133.8, 133.6, 132.2, 130.1, 129.6, 129.5, 129.1, 128.0 ppm. IR: ν = 3063, 1698, 1576, 1444, 1304, 1148, 1084, 915, 847, 749, 685 cm^{-1}.

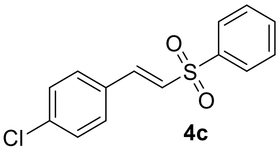

4c

(*E*)-1-chloro-4-(2-(phenylsulfonyl)vinyl)benzene (4c). Yield: 78.5 %. ^{1}H NMR (300 MHz, $CDCl_3$): δ = 8.05–7.85 (m, 2H), 7.65–7.51 (m, 4H), 7.43–7.32 (m, 4H), 6.82 (d, J = 15.4 Hz, 1H). ^{13}C NMR (75 MHz, $CDCl_3$): δ = 141.1, 140.7, 137.4, 133.7, 131.0, 129.9, 129.6, 129.5, 128.1, 127.9 ppm. IR: ν = 3060, 1616, 1587, 1486, 1441, 1302, 1298, 1145, 1084, 975, 860, 798, 685, 581 cm^{-1}.

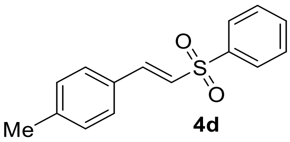

4d

(*E*)-1-methyl-4-(2-(phenylsulfonyl)vinyl)benzene (4d). Yield: 92.2 %. ^{1}H NMR (300 MHz, CD_2Cl_2): δ = 7.99–7.88 (m, 2H), 7.66–7.53 (m, 4H), 7.41 (d, J = 8.2 Hz, 2H), 7.22 (d, J = 8.1 Hz, 2H), 6.84 (d, J = 15.4 Hz, 1H), 2.37 (s, 3H) ppm. ^{13}C NMR (75 MHz, CD_2Cl_2): δ = 142.9, 142.4, 141.6, 133.7, 130.2, 129.7, 129.0, 127.9, 127.8, 126.7, 21.6. IR: ν = 3058, 2923, 1605, 1595, 1511, 1445, 1306, 1145, 1082, 980, 827, 788, 687 cm^{-1}.

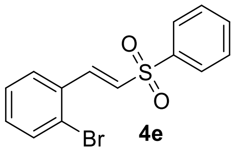

4e

(*E*)-1-bromo-2-(2-(phenylsulfonyl)vinyl)benzene (4e). Yield: >99.0 %. ^{1}H NMR (300 MHz, CD_2Cl_2): δ = 8.01 (d, J = 15.4 Hz, 1H), 7.96-7.86 (m, 2H), 7.66-7.52 (m, 5H), 7.34-7.27 (m, 2H), 6.88 (d, J = 15.4 Hz, 1H) ppm. ^{13}C NMR (75 MHz, CD_2Cl_2): δ = 141.2, 140.9, 134.0, 134.0, 133.0, 132.5, 130.9, 129.8, 128.7, 128.4, 128.2, 125.9 ppm. IR: ν = 3057, 1669, 1465, 1445, 1304, 1145, 1084, 970, 820, 748, 686, 568 cm^{-1}.

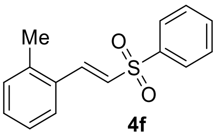

4f

(*E*)-1-methyl-2-(2-(phenylsulfonyl)vinyl)benzene (4f). Yield: >99.0 %. ^{1}H NMR (401 MHz, CD_2Cl_2): δ = 8.01–7.87 (m, 3H), 7.66–7.55 (m, 3H), 7.46 (d, J = 7.9 Hz, 1H), 7.33 – 7.29 (m, 1H), 7.26–7.18 (m, 2H), 6.82 (d, J = 15.4 Hz, 1H), 2.46 (s, 3H) ppm. ^{13}C NMR (101 MHz, CD_2Cl_2): δ = 141.3, 140.5, 138.7, 133.7, 131.8, 131.4, 131.3, 129.7, 128.7, 128.0, 127.2, 126.8, 19.9 ppm. IR: ν = 3057, 2918, 1613, 1442, 1301, 1147, 1082, 970, 834 734, 687 cm^{-1}.

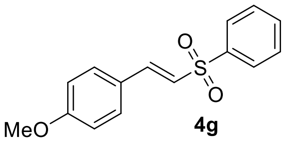

4g

(*E*)-1-methoxy-4-(2-(phenylsulfonyl)vinyl)benzene (4g). Yield: 96.8 %. ^{1}H NMR (300 MHz, $CDCl_3$): δ = 7.96–7.93 (m, 2H), 7.64 (d, J = 15.2 Hz, 1H), 7.58–7.49 (m, 3H), 7.42 (d, J = 8.8 Hz, 2H), 6.90 (d, J = 8.8 Hz, 2H), 6.70 (d, J = 15.4 Hz, 1H), 3.83 (s, 3H) ppm. ^{13}C NMR (75 MHz, $CDCl_3$): δ = 162.1, 142.3, 141.2, 133.1, 130.4, 129.3, 127.5, 125.0, 124.5, 114.5, 55.4 ppm. IR: ν = 3057, 2920, 2850, 1601, 1587, 1510, 1443, 1304, 1255, 1173, 1131, 1082, 1024, 976, 863, 743, 689 cm^{-1}.

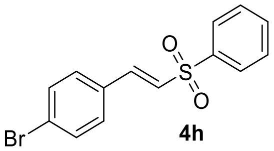

4h

(*E*)-1-bromo-4-(2-(phenylsulfonyl)vinyl)benzene (4h). Yield: 76.7 %. ^{1}H NMR (401 MHz, $CDCl_3$): δ = 7.96–7.93 (m, 2H), 7.67–7.51 (m, 6H), 7.35 (d, J = 8.5 Hz, 2H), 6.85 (d, J = 15.4 Hz, 1H) ppm. ^{13}C NMR (101 MHz, $CDCl_3$): δ = 140.0, 139.4, 132.5, 131.4, 130.3, 128.9, 128.4, 127.0, 126.7,

124.7 ppm. IR: ν = 3054, 2921, 2854, 1670, 1586, 1486, 1445, 1400, 1305, 1143, 1071, 1009, 970, 857, 791, 721, 685, 649, 573, 548 cm^{-1}.

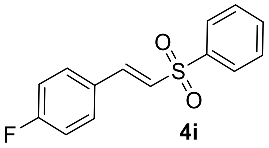

4i

(*E*)-1-fluoro-4-(2-(phenylsulfonyl)vinyl)benzene (4i). Yield: 79.4 %. ^{1}H NMR (300 MHz, CD_2Cl_2): δ = 7.97–7.88 (m, 2H), 7.68–7.50 (m, 6H), 7.11 (t, J = 8.7 Hz, 2H), 6.83 (d, J = 15.4, 1H) ppm. ^{13}C NMR (75 MHz, CD_2Cl_2): δ = 165.3, 161.9, 140.3, 132.6, 130.0, 129.8, 128.6, 126.8, 126.6, 126.6, 115.6, 115.3 ppm. ^{19}F NMR (377 MHz, CD_2Cl_2): δ = -108.8 ppm. IR: ν = 3055, 2922, 1600, 1507, 1446, 1305, 1220, 1150, 1143, 1082, 971, 750, 686, 594 cm^{-1}.

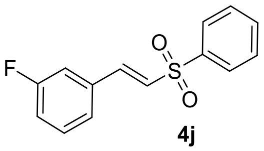

4j

(*E*)-1-fluoro-3-(2-(phenylsulfonyl)vinyl)benzene (4j). Yield: 77.7 %. ^{1}H NMR (300 MHz, CD_2Cl_2): δ = 7.96–7.92 (m, 2H), 7.66–7.61 (m, 1H), 7.61–7.56 (m, 3H), 7.49–7.25 (m, 2H), 7.25–7.20 (m, 1H), 7.19–7.09 (m, 1H), 6.91 (d, J = 15.5 Hz, 1H) ppm. ^{13}C NMR (75 MHz, CD_2Cl_2): δ = 165.0, 161.7, 141.3, 141.2, 140.9, 135.2, 135.1, 134.0, 133.9, 131.2, 131.1, 129.8, 128.0, 125.0, 125.0, 118.4, 118.1, 115.3, 115.0 ppm. ^{19}F NMR (377 MHz, CD_2Cl_2): δ = -112.8 ppm. IR: ν = 3061, 2925, 1716, 1612, 1583, 1482, 1446, 1304, 1261, 1141, 1083, 963, 869, 780, 686, 536 cm^{-1}.

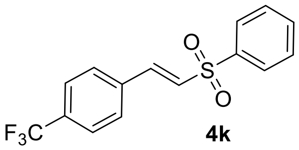

4k

(*E*)-1-(2-(phenylsulfonyl)vinyl)-4-(trifluoromethyl)benzene (4k). Yield: 84.8 %. ^{1}H NMR (300 MHz, CD_2Cl_2): δ = 7.98–7.93 (m, 2H), 7.73–7.56 (m, 8H), 7.00 (d, J = 15.5 Hz, 1H) ppm. ^{13}C NMR (75 MHz, CD_2Cl_2): δ = 140.8, 140.7, 136.4, 134.0, 132.9, 132.4, 130.6, 129.8, 129.2, 128.1, 126.4, 126.4, 126.3, 126.3, 126.0, 122.4 ppm. ^{19}F NMR (282 MHz, CD_2Cl_2): δ = -63.3 ppm. IR: ν = 3053, 2923, 1615, 1446, 1413, 1319, 1144, 1064, 1015, 974, 861, 819, 802, 715, 687, 567, 548, 504 cm^{-1}.

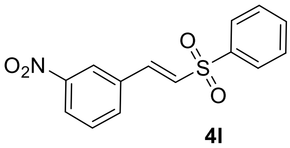

4l

(*E*)-1-nitro-3-(2-(phenylsulfonyl)vinyl)benzene (4l). Yield: 29.6 %. ^{1}H NMR (300 MHz, CD_2Cl_2): δ = 8.36–8.34 (m, 1H), 8.25–8.23 (m, 1H), 7.98–7.94 (m, 2H), 7.84 (m, 1H), 7.76–7.56 (m, 5H), 7.05 (d, J = 15.5 Hz, 1H) ppm. ^{13}C NMR (75 MHz, CD_2Cl_2): δ = 149.1, 140.5, 139.8, 134.7, 134.6, 134.2, 131.2, 130.6, 129.9, 128.2, 125.6, 123.2 ppm. IR: ν = 3062, 2923, 2852, 1689, 1615, 1525, 1446, 1349, 1304, 1145, 1085, 970, 843, 809, 733, 686, 567, 534 cm^{-1}.

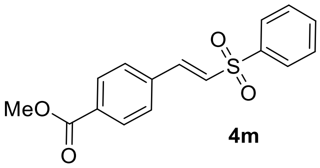

4m

Methyl (*E*)-4-[(2-phenylethenyl)sulfonyl]benzoate (4m). Yield: 82.9 %. ^{1}H NMR (300 MHz, CD_2Cl_2): δ = 8.07– 8.00 (d, J = 8.4 Hz, 2H), 7.97– 7.91 (m, 2H), 7.66–7.43 (m, 6H), 6.90 (d, J = 15.5 Hz, 1H), 3.81 (s, 3H) ppm. ^{13}C NMR (101 MHz, C_6D_6): δ = 167.9, 142.7, 142.2, 138.4, 135.4, 134.1, 131.9, 131.7, 131.3, 130.3, 129.5, 54.0 ppm. IR: ν = 3046, 2919, 2849, 1715, 1608, 1566, 1434, 1277, 1191, 1143, 1106, 1183, 1016, 959, 863, 826, 752, 718, 686, 654, 575, 546 cm^{-1}.

NMR spectra.

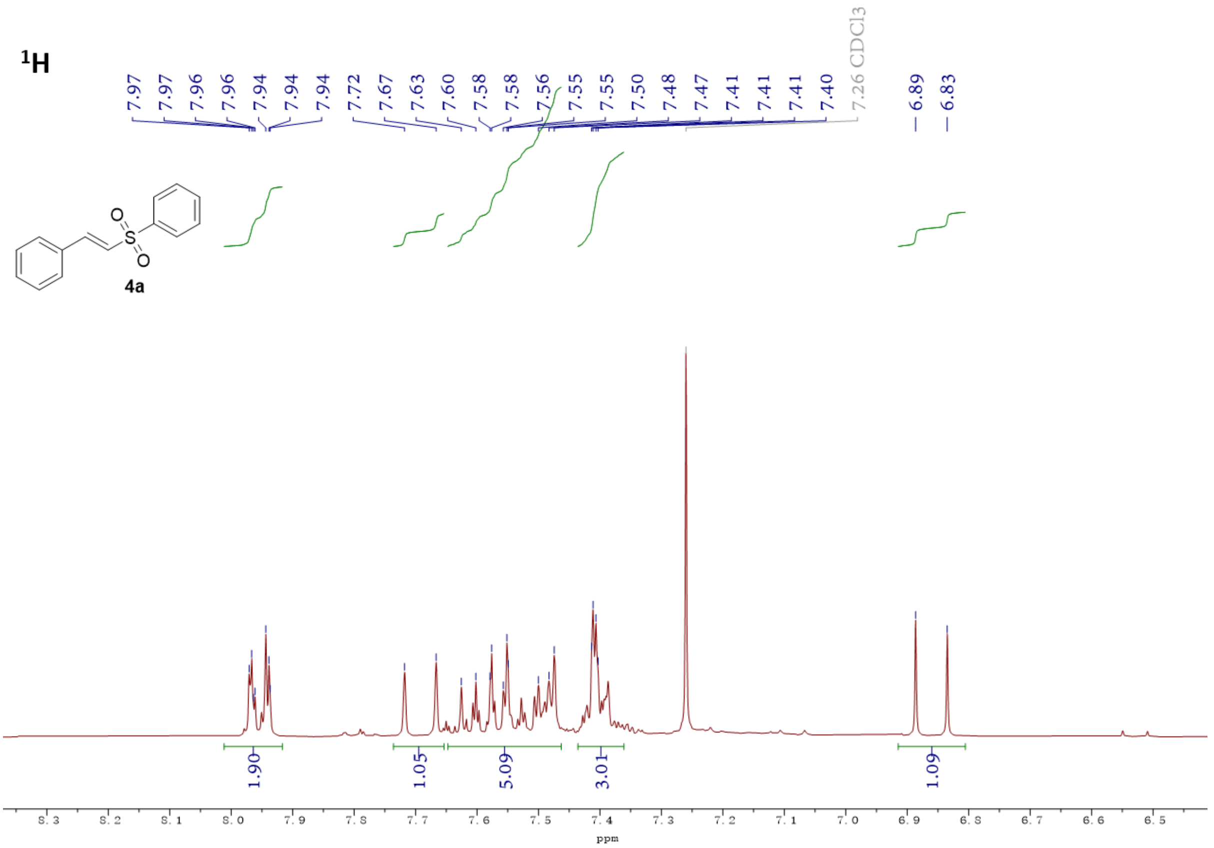
1H
7.97
7.97
7.96
7.96
7.94
7.94
7.94
7.72
7.67
7.63
7.60
7.58
7.58
7.56
7.55
7.55
7.50
7.48
7.47
7.41
7.41
7.41
7.40
7.26 CDCl3
6.89
6.83
4a
1.90
1.05
5.09
3.01
1.09
ppm

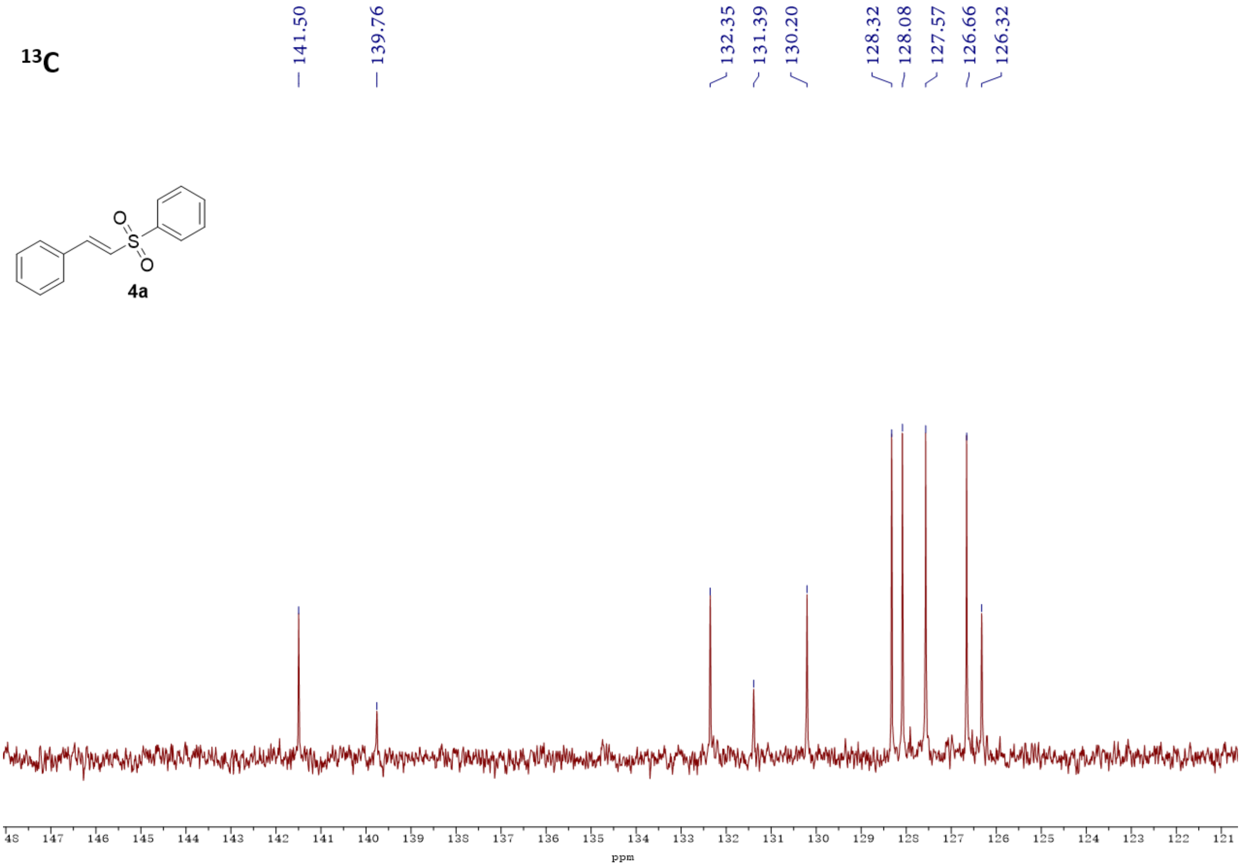
13C
141.50
139.76
132.35
131.39
130.20
128.32
128.08
127.57
126.66
126.32
4a
ppm

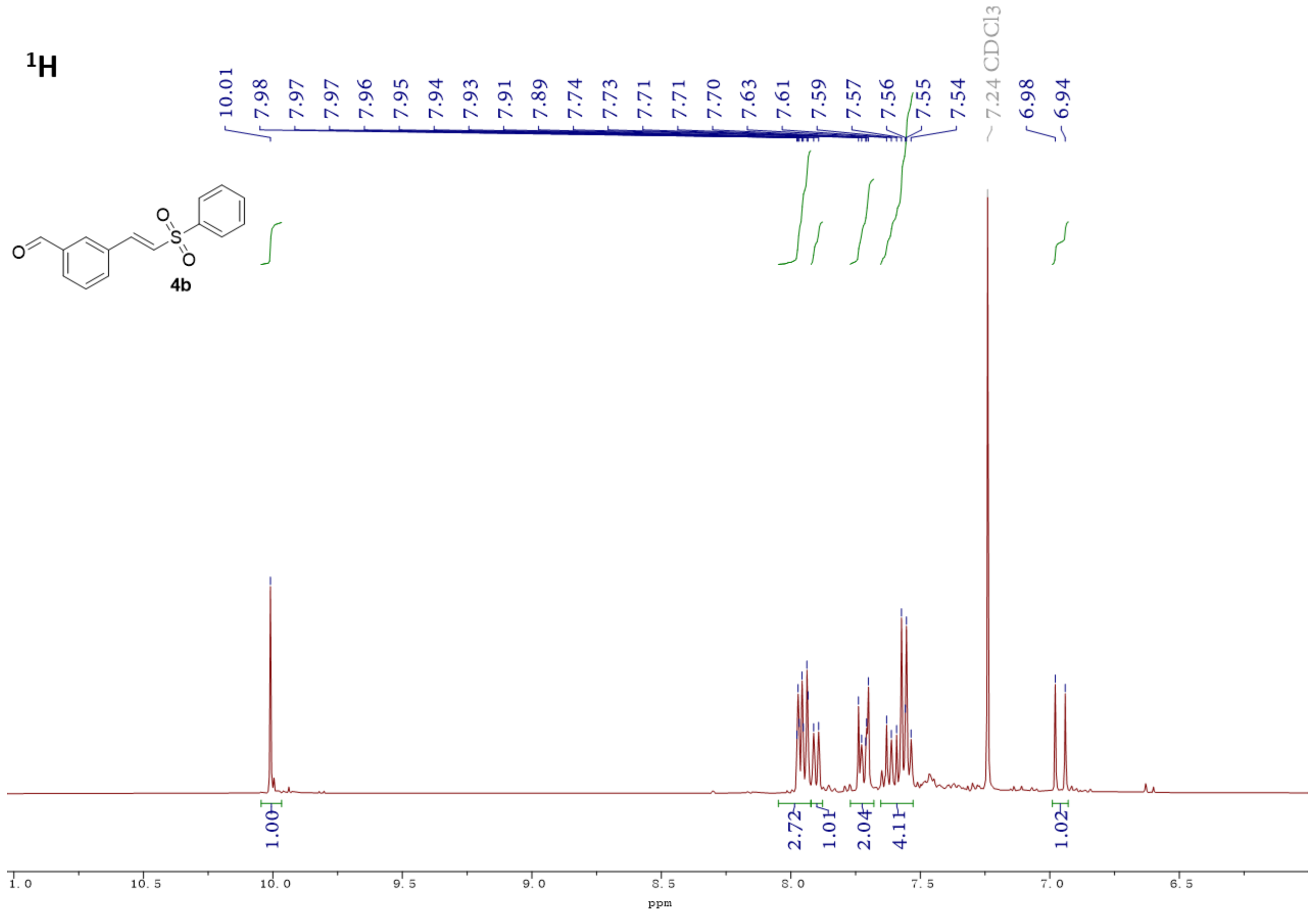
1H
4b
10.01
7.98
7.97
7.97
7.96
7.95
7.94
7.93
7.91
7.89
7.74
7.73
7.71
7.71
7.70
7.63
7.61
7.59
7.57
7.56
7.55
7.54
7.24 CDCl3
6.98
6.94
1.00
2.72
1.01
2.04
4.11
1.02
ppm

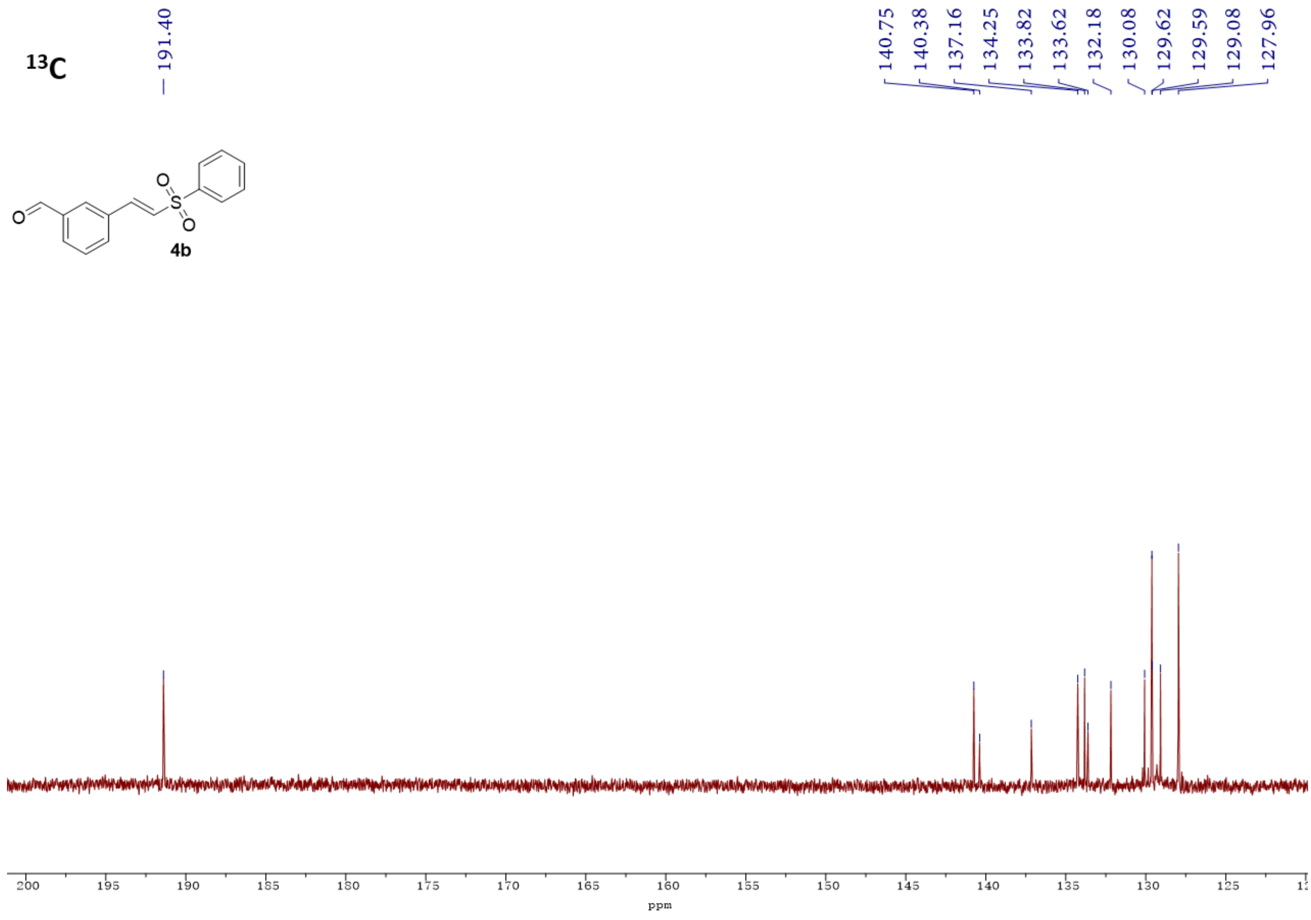
13C
4b
191.40
140.75
140.38
137.16
134.25
133.82
133.62
132.18
130.08
129.62
129.59
129.08
127.96
ppm

^{1}H

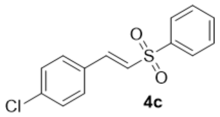

4c

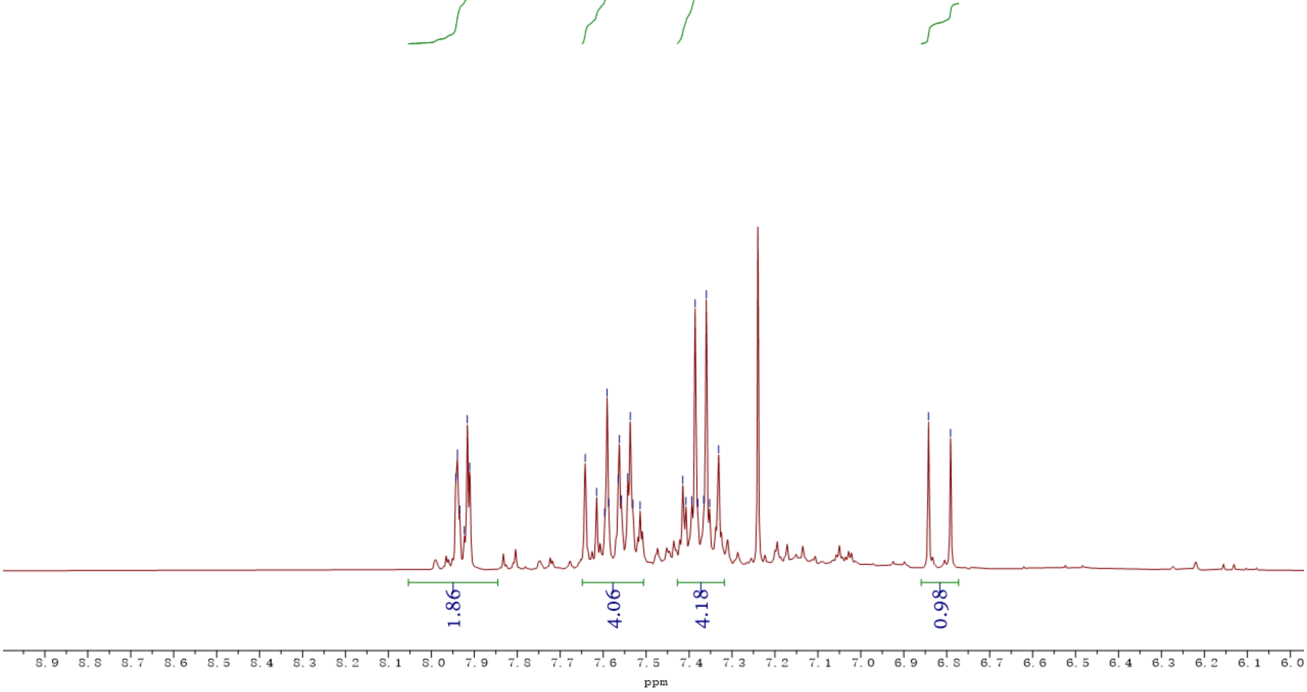
7.94
7.94
7.93
7.92
7.92
7.91
7.64
7.62
7.59
7.59
7.57
7.56
7.56
7.54
7.54
7.53
7.41
7.41
7.39
7.39
7.38
7.37
7.36
7.35
7.33
7.24 CDCl3
6.84
1.86
4.06
4.18
0.98
8.9 8.8 8.7 8.6 8.5 8.4 8.3 8.2 8.1 8.0 7.9 7.8 7.7 7.6 7.5 7.4 7.3 7.2 7.1 7.0 6.9 6.8 6.7 6.6 6.5 6.4 6.3 6.2 6.1 6.0
ppm

^{13}C

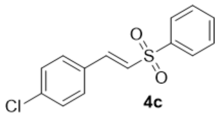

4c

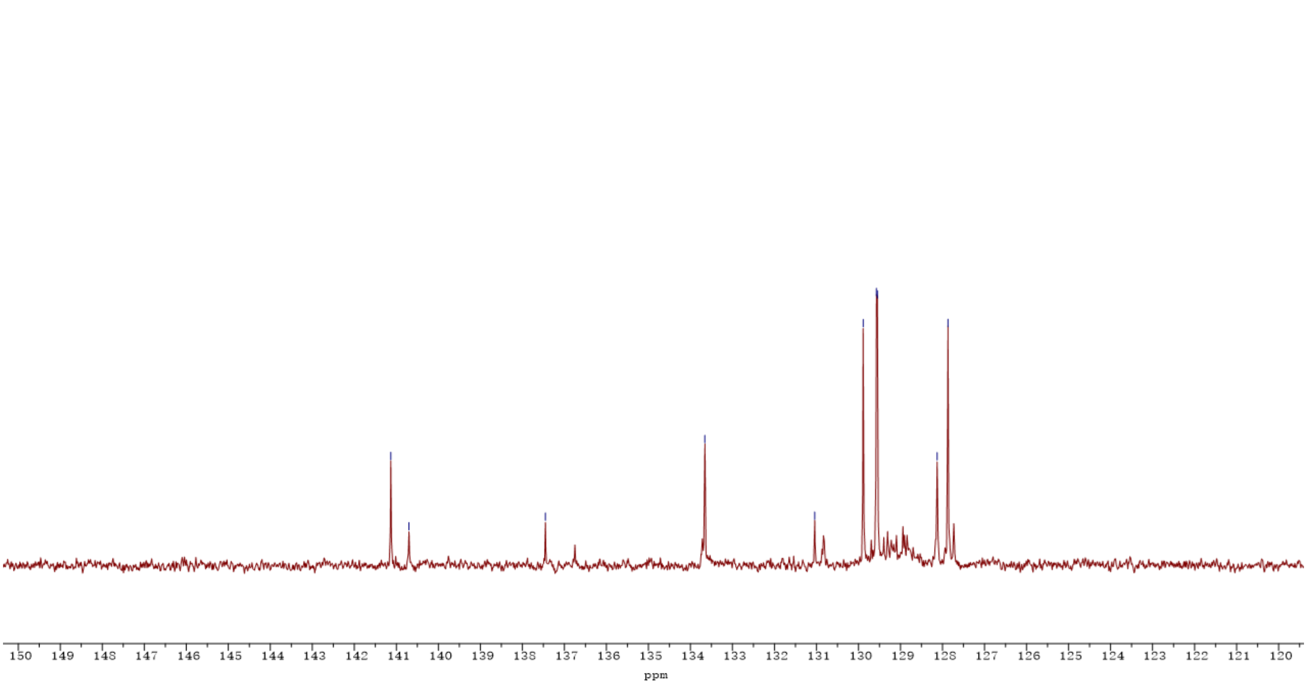
141.13
140.70
137.45
133.66
131.04
129.89
129.57
129.55
128.13
127.87
150 149 148 147 146 145 144 143 142 141 140 139 138 137 136 135 134 133 132 131 130 129 128 127 126 125 124 123 122 121 120
ppm

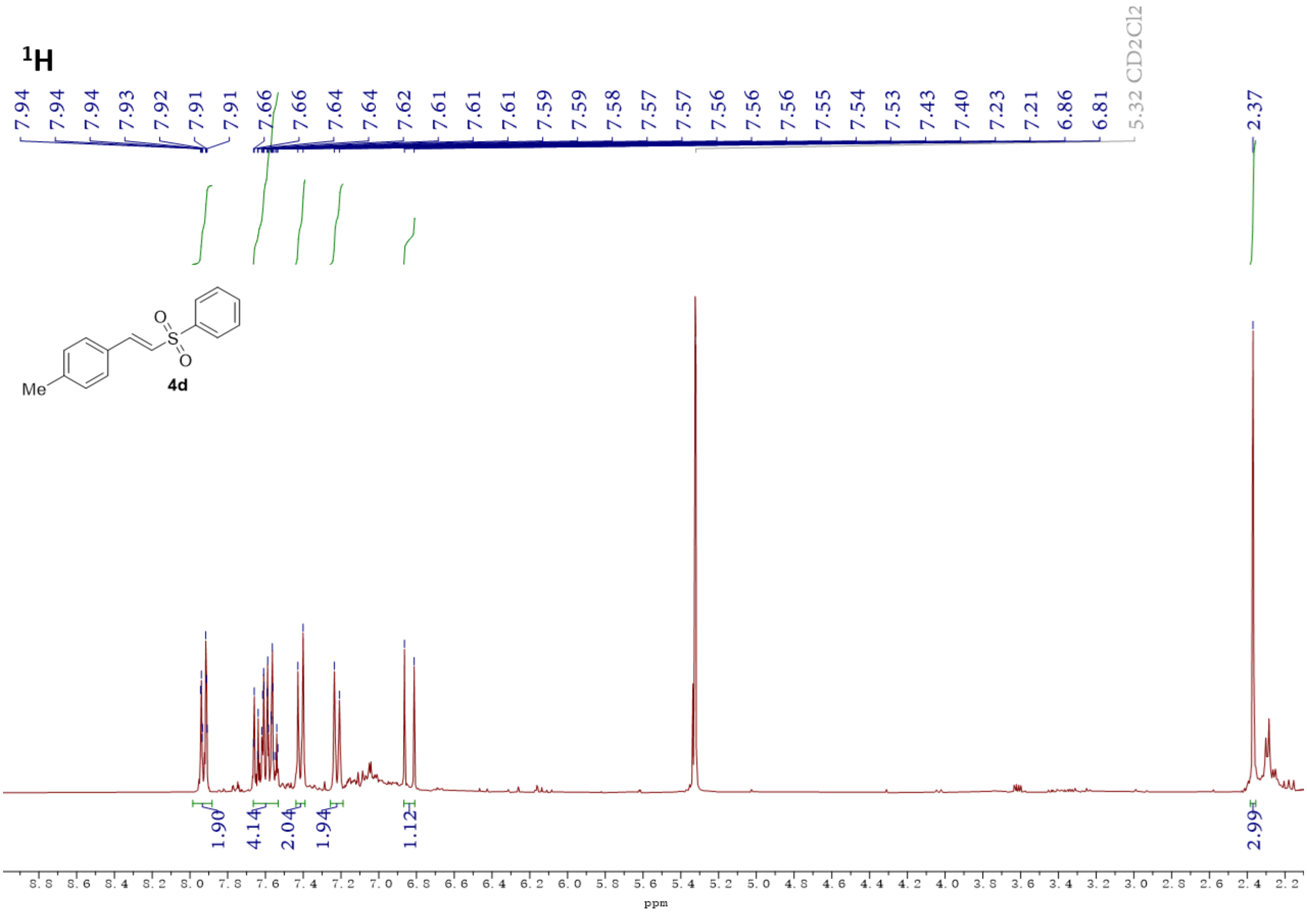

1H
7.94
7.94
7.94
7.93
7.92
7.91
7.91
7.66
7.66
7.64
7.64
7.62
7.61
7.61
7.61
7.59
7.59
7.58
7.57
7.57
7.56
7.56
7.56
7.55
7.54
7.53
7.43
7.40
7.23
7.21
6.86
6.81
5.32 CD2Cl2
2.37
Me
4d
1.90
4.14
2.04
1.94
1.12
2.99
ppm

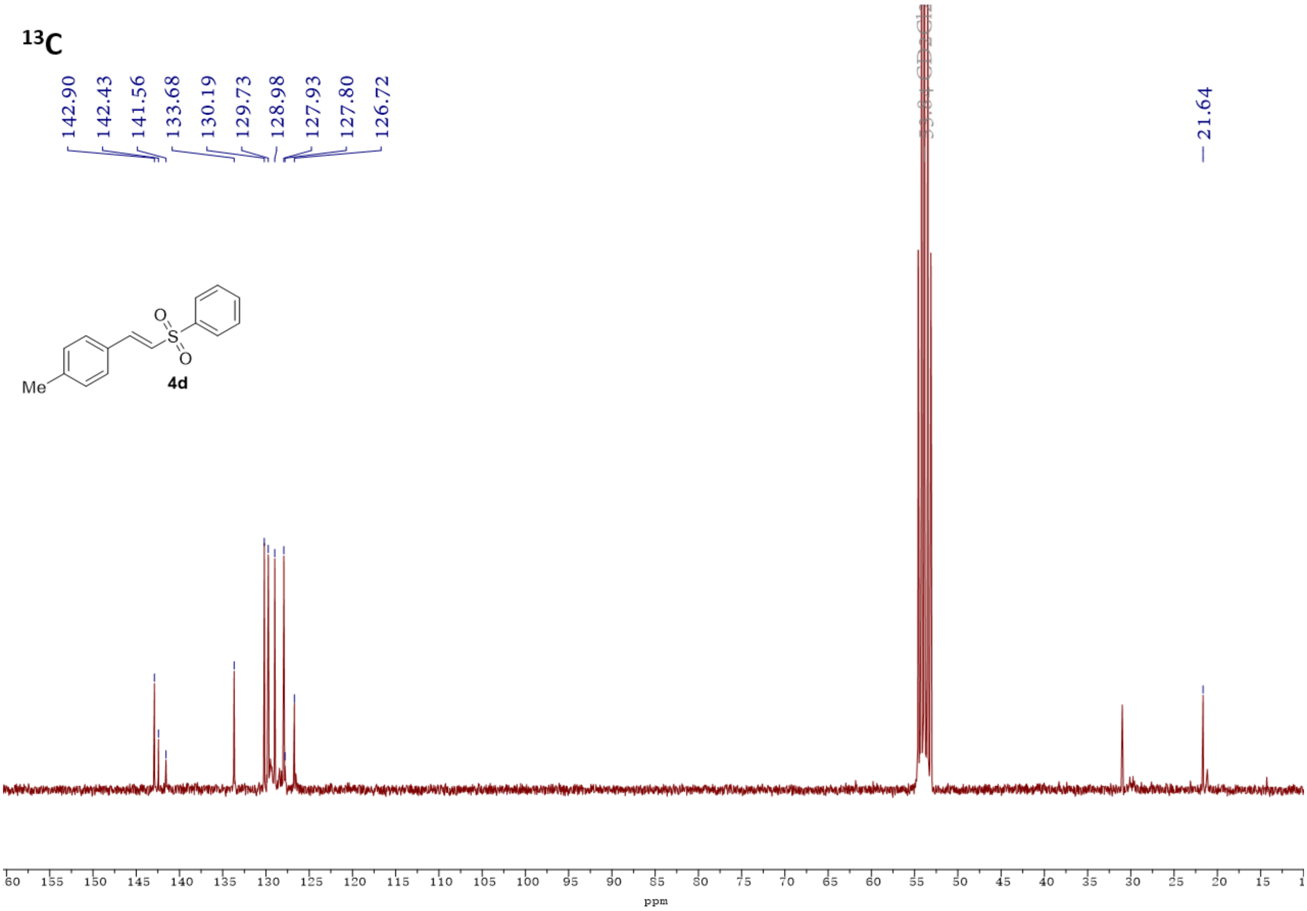

13C
142.90
142.43
141.56
133.68
130.19
129.73
128.98
127.93
127.80
126.72
21.64
Me
4d
ppm

[1]H

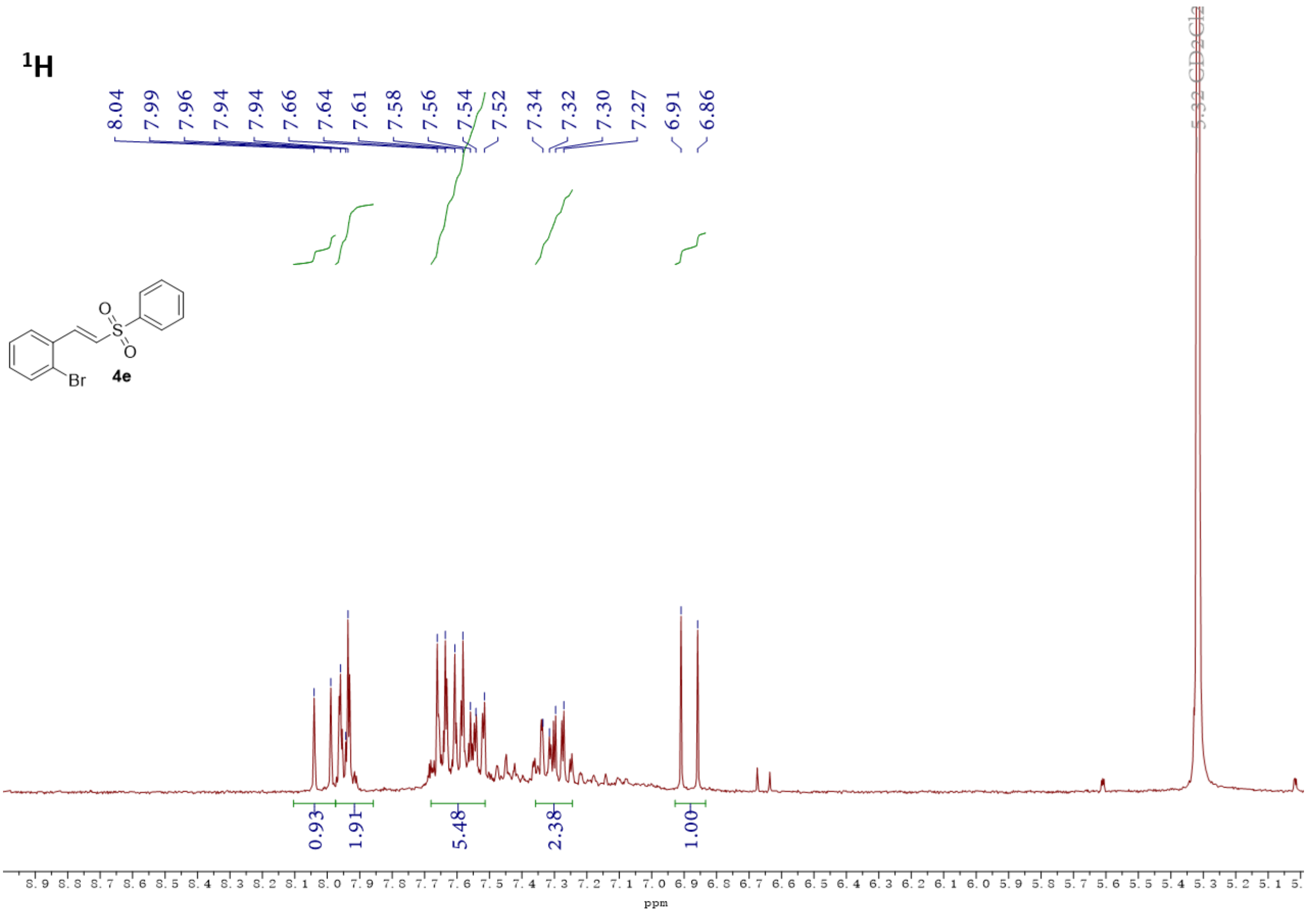
8.04 7.99 7.96 7.94 7.94 7.66 7.64 7.61 7.58 7.56 7.54 7.52 7.34 7.32 7.30 7.27 6.91 6.86
5.32 CD2Cl2
Br
4e
0.93 1.91 5.48 2.38 1.00
ppm

[13]C

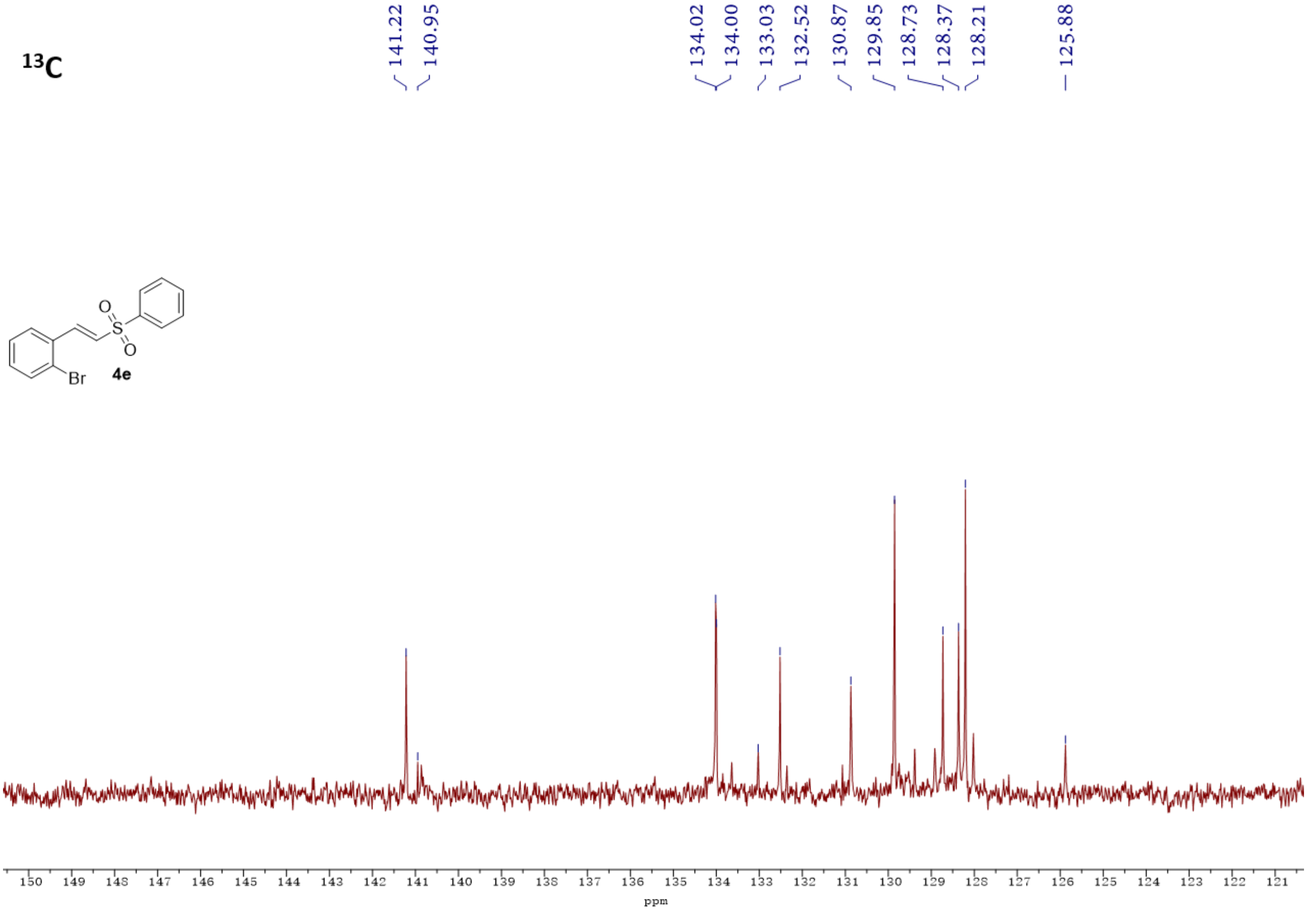
141.22 140.95 134.02 134.00 133.03 132.52 130.87 129.85 128.73 128.37 128.21 125.88
Br
4e
ppm

^{1}H

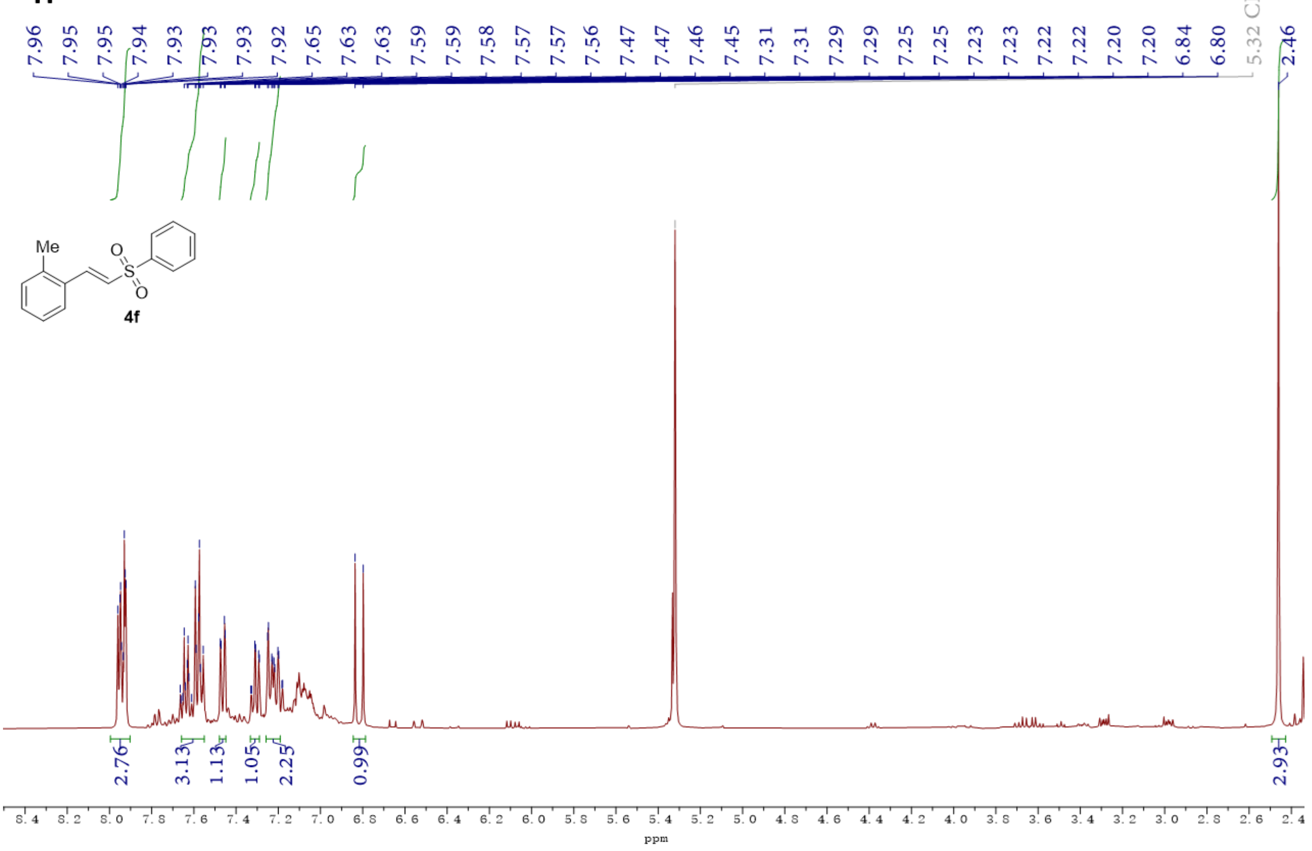
7.96 7.95 7.95 7.94 7.93 7.93 7.93 7.92 7.65 7.63 7.63 7.59 7.59 7.58 7.57 7.57 7.56 7.47 7.47 7.46 7.45 7.31 7.31 7.29 7.29 7.25 7.25 7.23 7.23 7.22 7.22 7.20 7.20 6.84 6.80
5.32 CD2Cl2
2.46
Me
O
S
O
4f
2.76
3.13
1.13
1.05
2.25
0.99
2.93
ppm

^{13}C

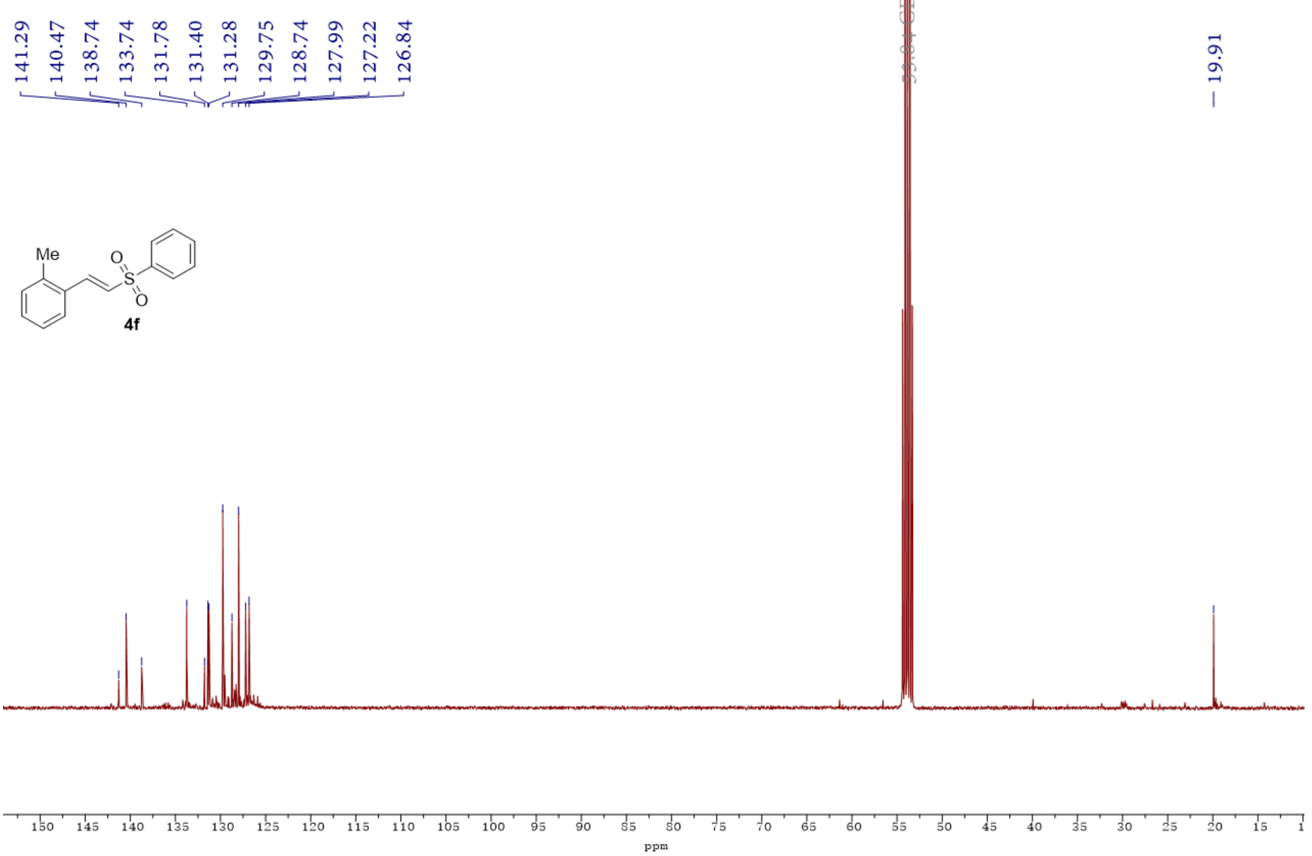
141.29 140.47 138.74 133.74 131.78 131.40 131.28 129.75 128.74 127.99 127.22 126.84
19.91
Me
O
S
O
4f
ppm

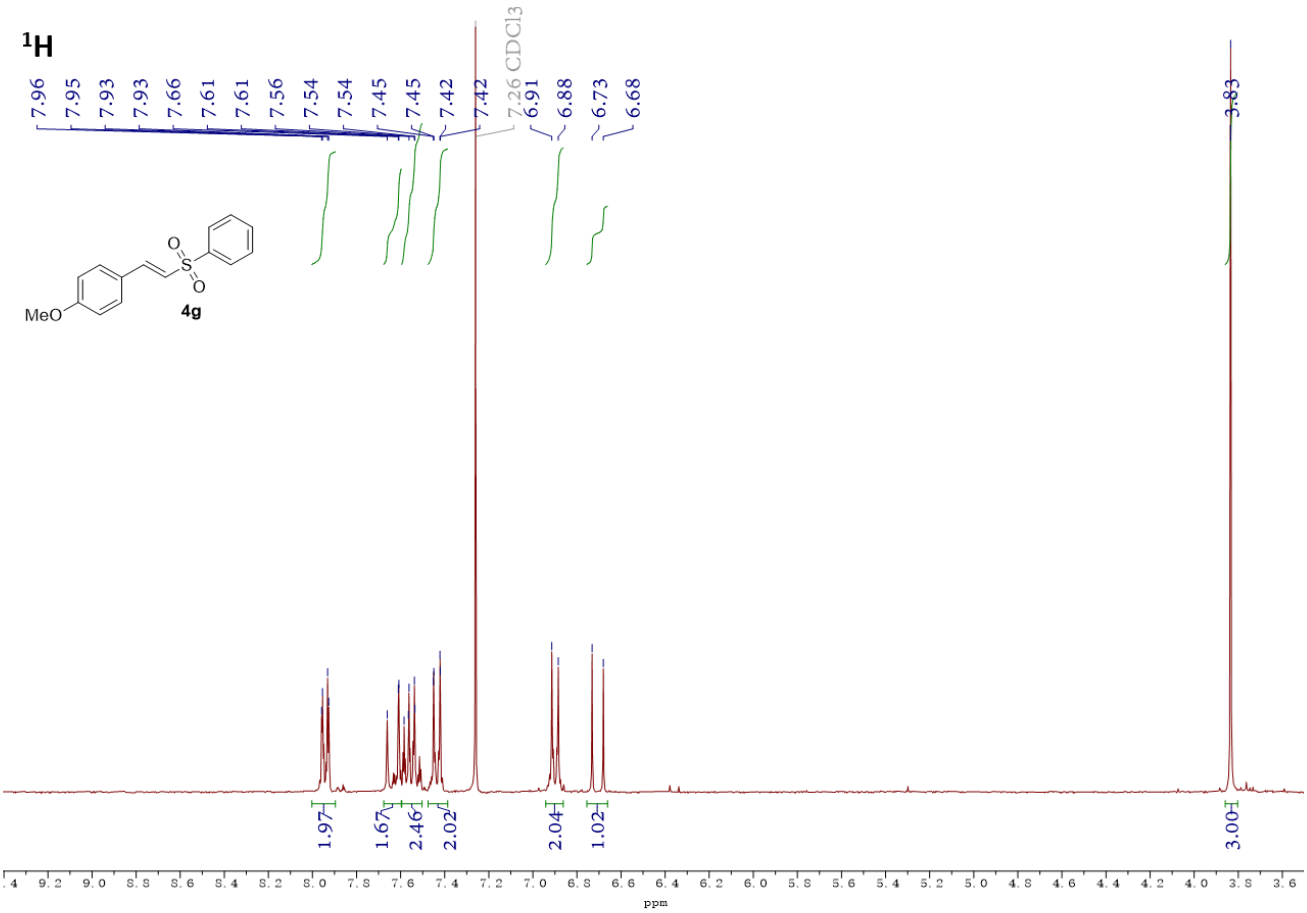
1H
7.96 7.95 7.93 7.93 7.66 7.61 7.61 7.56 7.54 7.54 7.45 7.45 7.42 7.42 7.26 CDCl3 6.91 6.88 6.73 6.68 3.83
MeO
4g
1.97 1.67 2.46 2.02 2.04 1.02 3.00
ppm

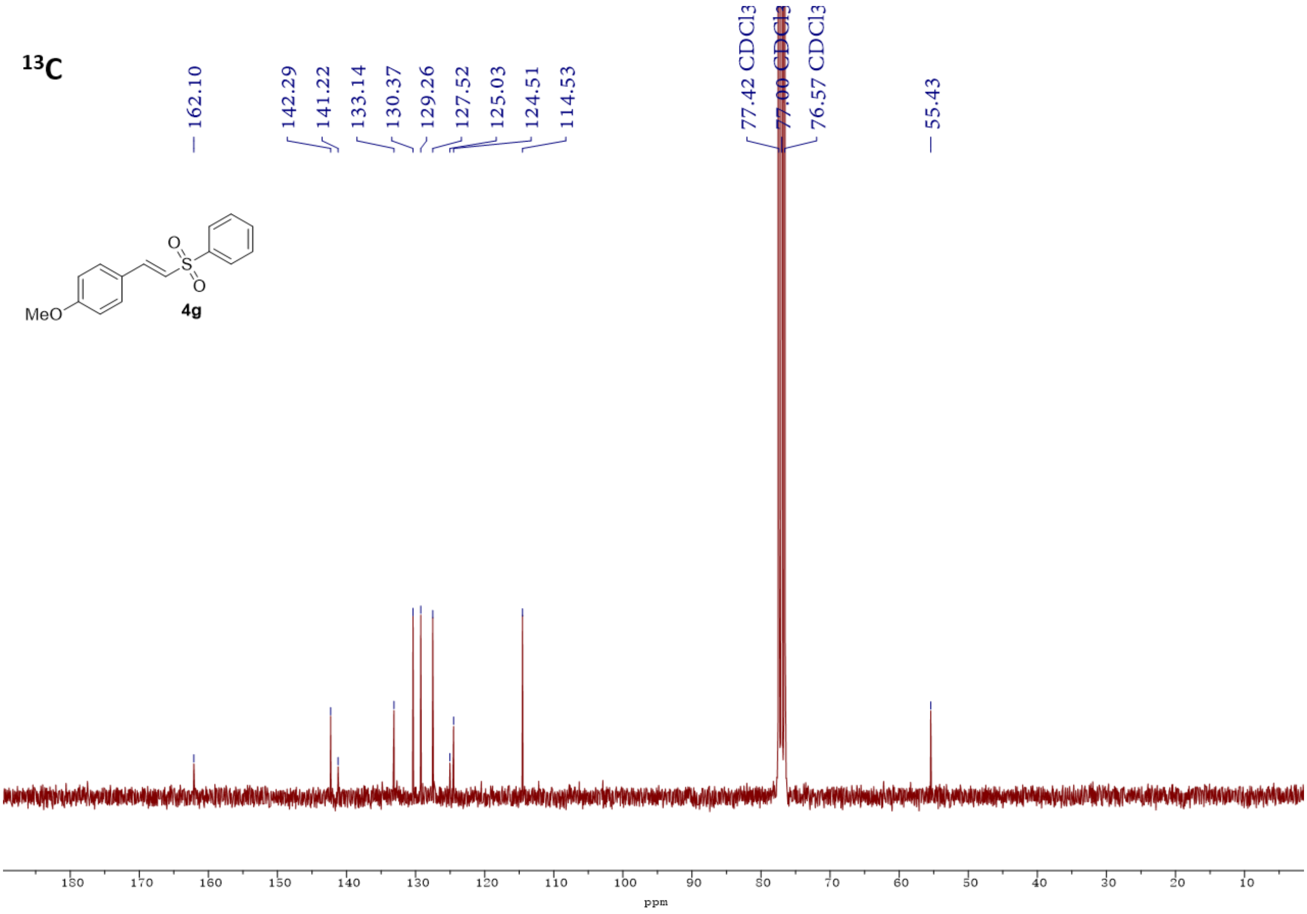
13C
162.10
142.29 141.22 133.14 130.37 129.26 127.52 125.03 124.51 114.53
77.42 CDCl3 77.00 CDCl3 76.57 CDCl3
55.43
MeO
4g
ppm

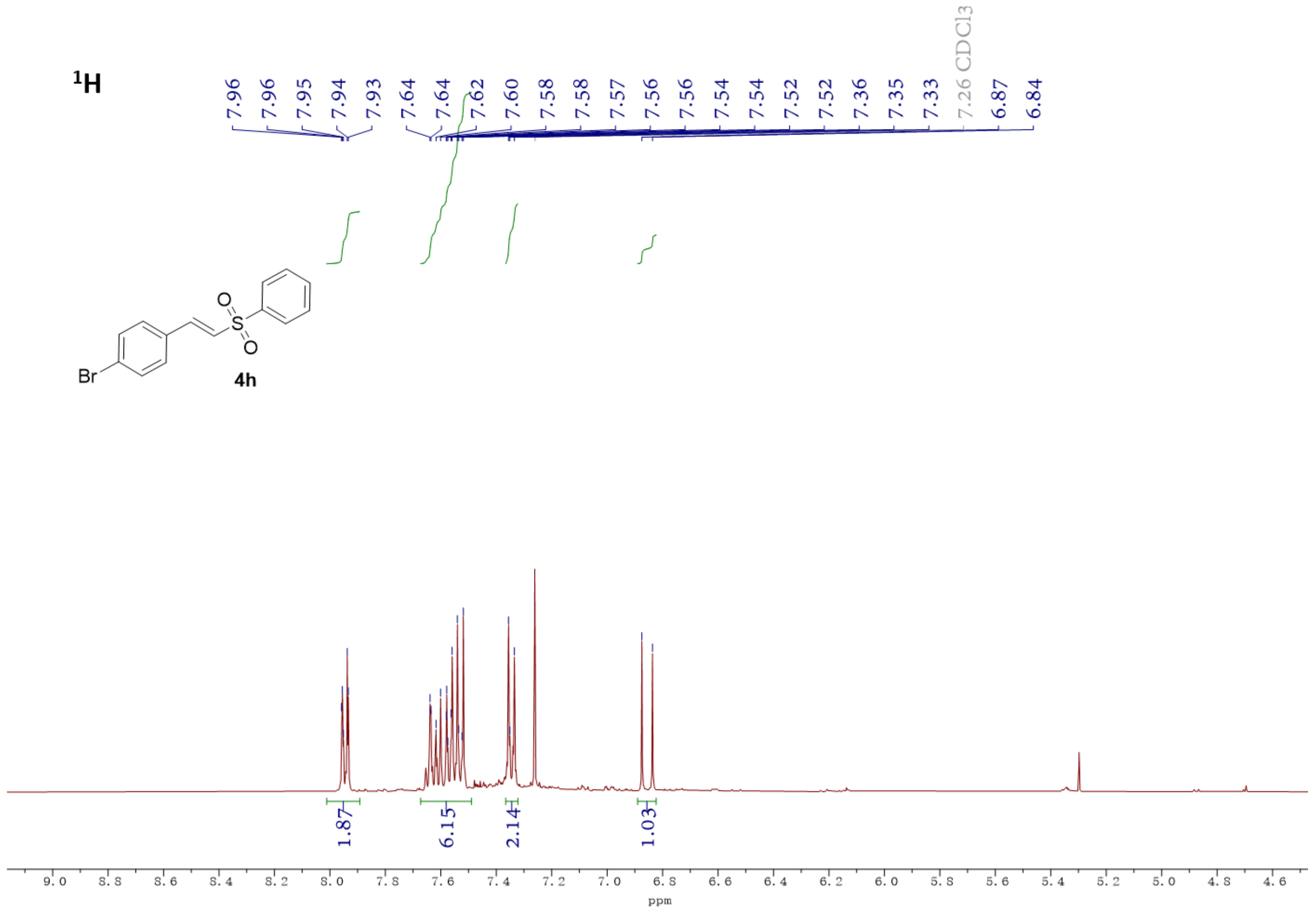
1H
7.96
7.96
7.95
7.94
7.93
7.64
7.64
7.62
7.60
7.58
7.58
7.57
7.56
7.56
7.54
7.54
7.52
7.52
7.36
7.35
7.33
7.26 CDCl3
6.87
6.84
Br
O
S
O
4h
1.87
6.15
2.14
1.03
ppm

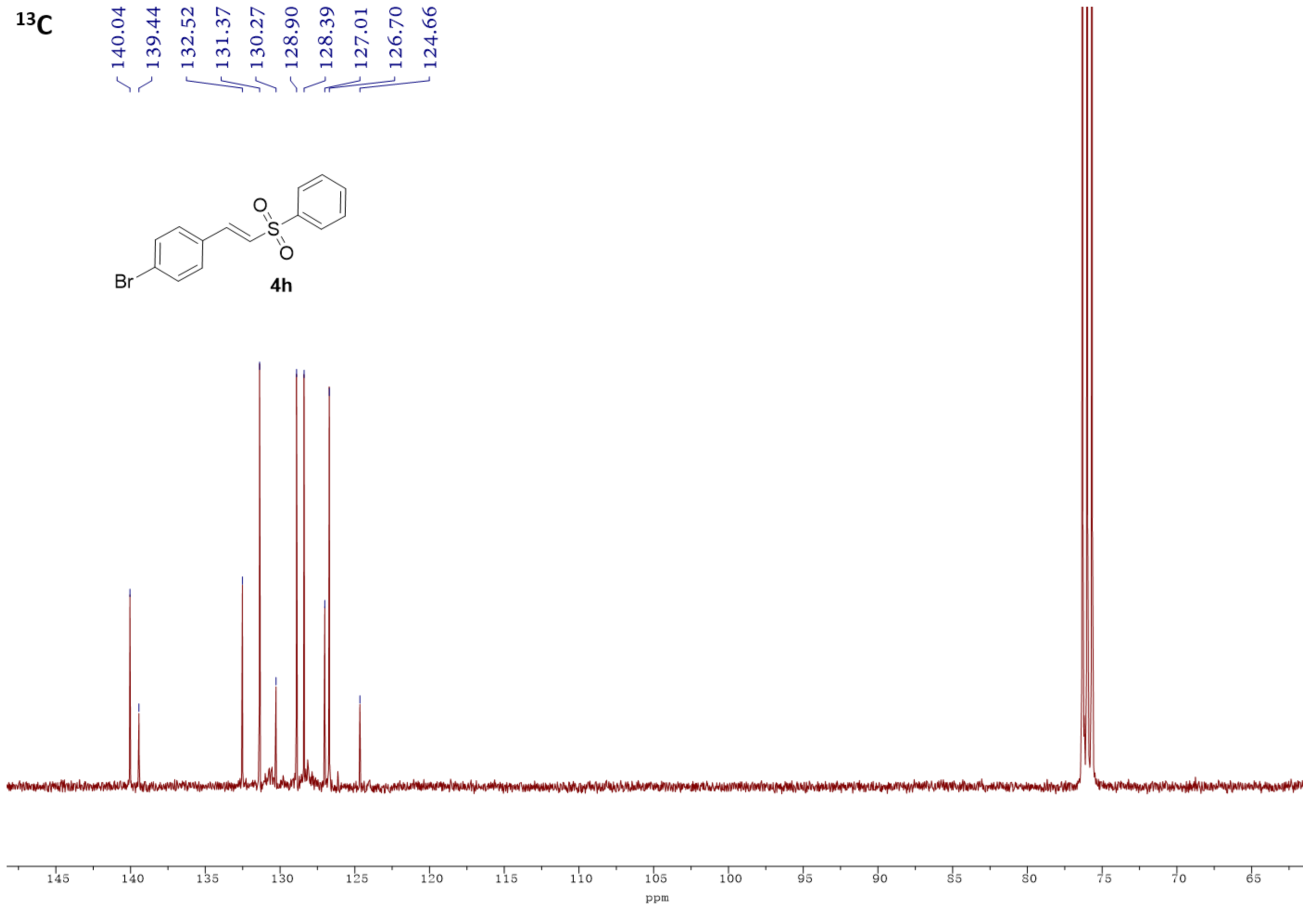
13C
140.04
139.44
132.52
131.37
130.27
128.90
128.39
127.01
126.70
124.66
Br
O
S
O
4h
ppm

^{1}H

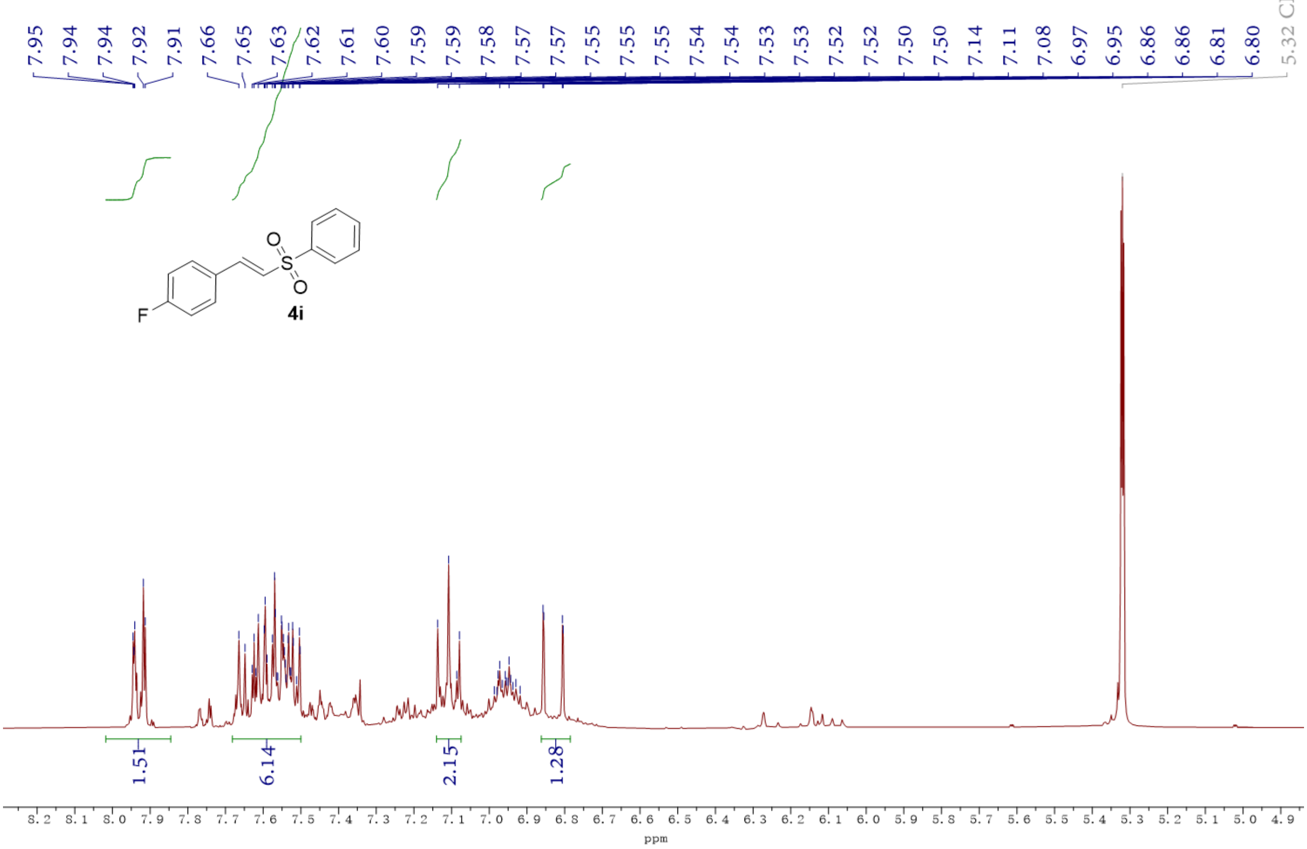

7.95 7.94 7.94 7.92 7.91 7.66 7.65 7.63 7.62 7.61 7.60 7.59 7.59 7.58 7.57 7.57 7.55 7.55 7.55 7.54 7.54 7.53 7.53 7.52 7.52 7.50 7.50 7.14 7.11 7.08 6.97 6.95 6.86 6.86 6.81 6.80
5.32 CD2Cl2
F
O
S
O
4i
1.51
6.14
2.15
1.28
8.2 8.1 8.0 7.9 7.8 7.7 7.6 7.5 7.4 7.3 7.2 7.1 7.0 6.9 6.8 6.7 6.6 6.5 6.4 6.3 6.2 6.1 6.0 5.9 5.8 5.7 5.6 5.5 5.4 5.3 5.2 5.1 5.0 4.9
ppm

^{13}C

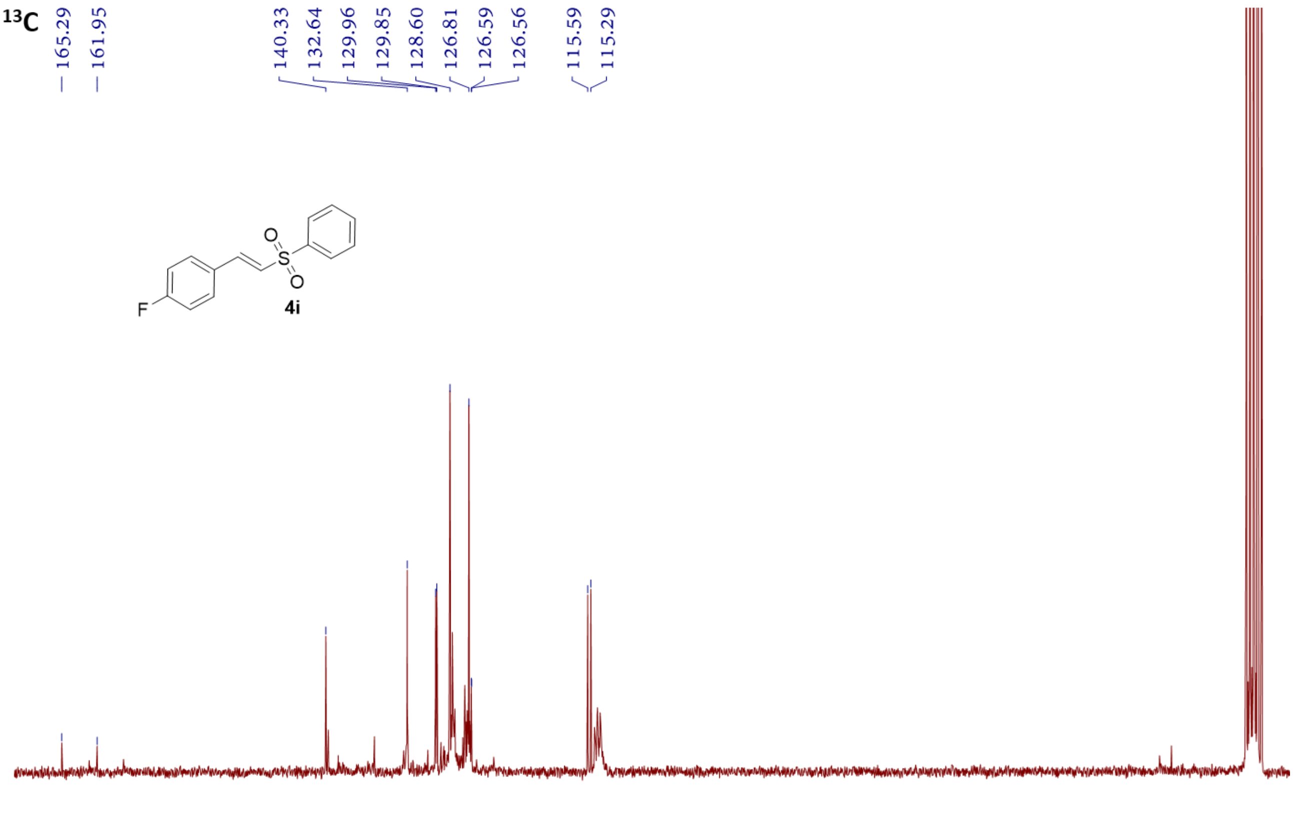

165.29
161.95
140.33 132.64 129.96 129.85 128.60 126.81 126.59 126.56
115.59 115.29
F
O
S
O
4i
165 160 155 150 145 140 135 130 125 120 115 110 105 100 95 90 85 80 75 70 65 60 55 50
ppm

^{19}F

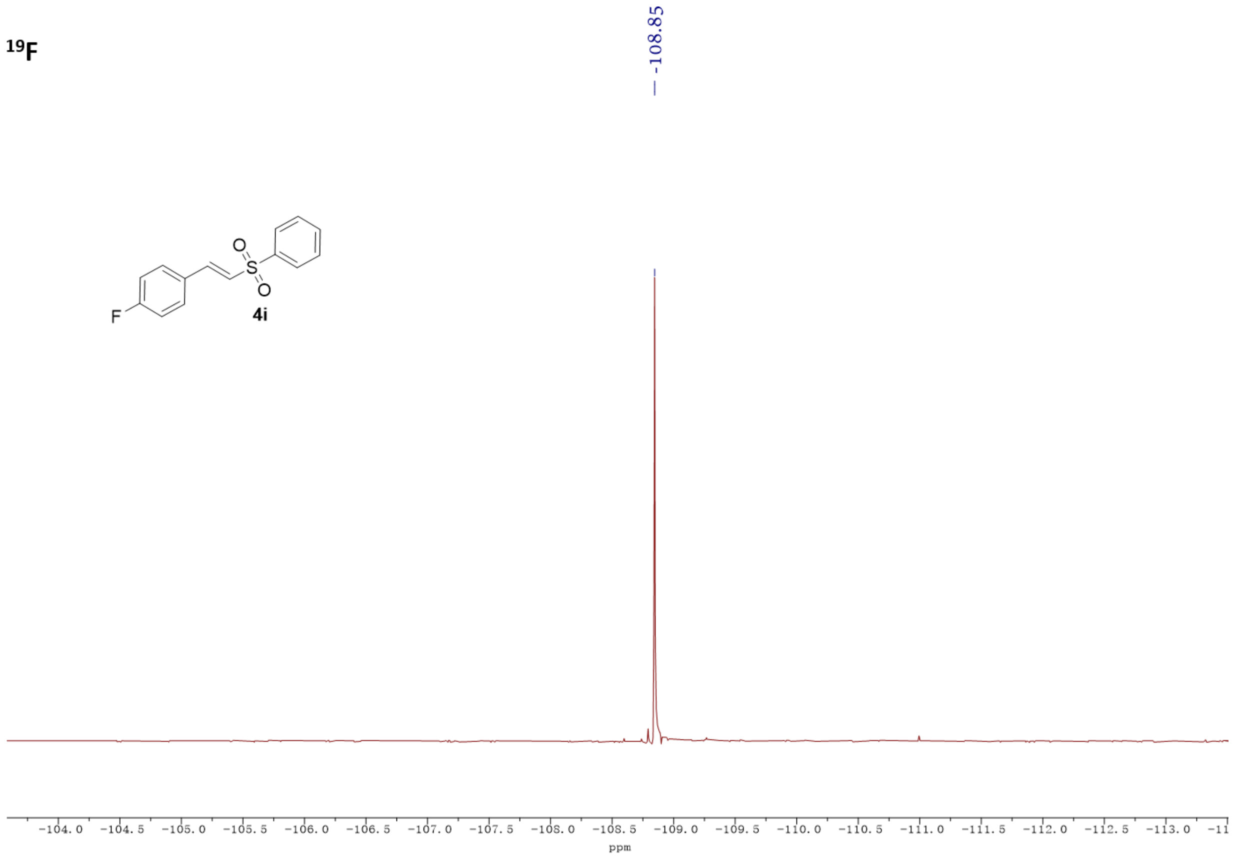

-108.85
F
O
S
O
-104.0 -104.5 -105.0 -105.5 -106.0 -106.5 -107.0 -107.5 -108.0 -108.5 -109.0 -109.5 -110.0 -110.5 -111.0 -111.5 -112.0 -112.5 -113.0
ppm

4i

^{1}H

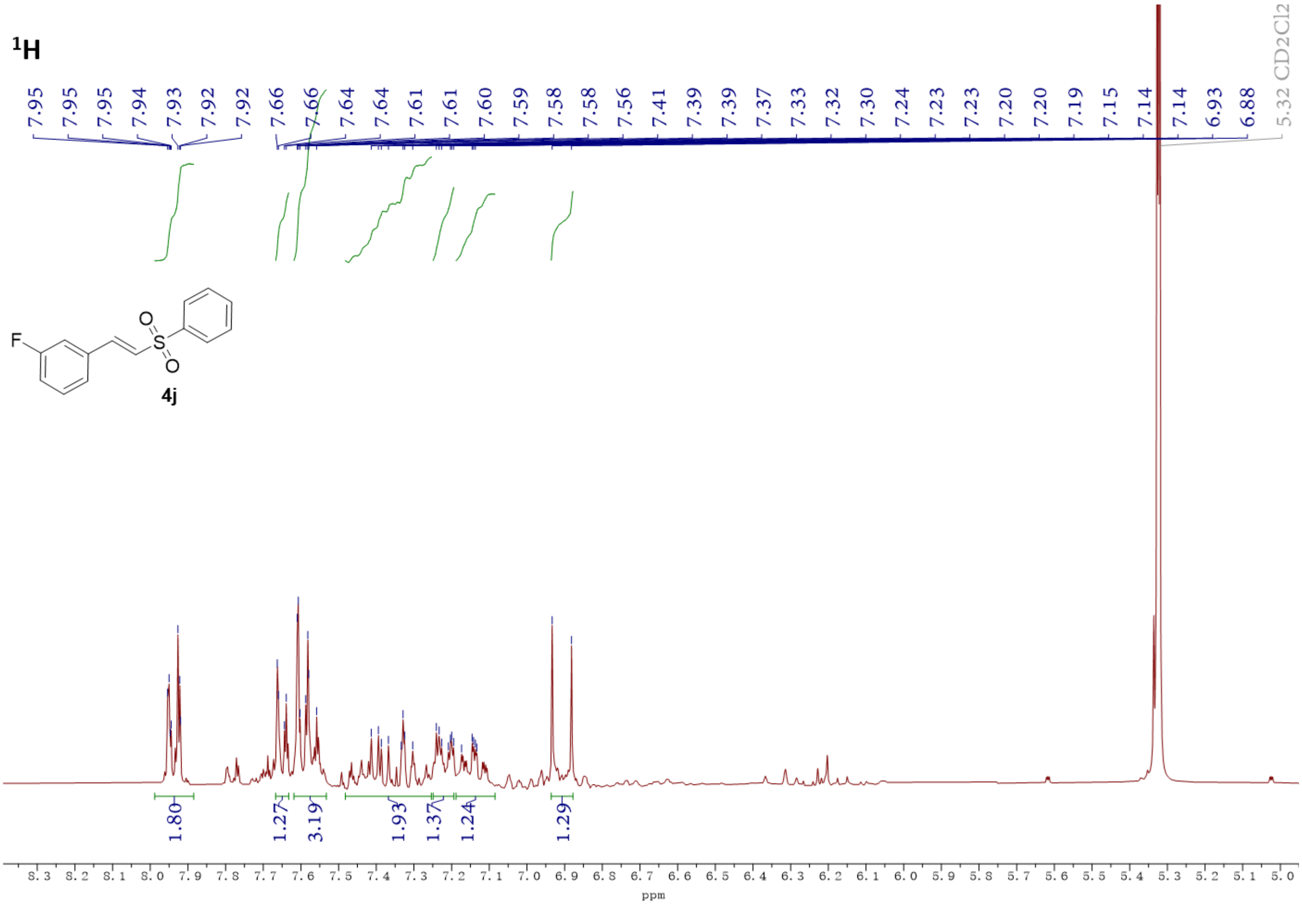

7.95 7.95 7.95 7.94 7.93 7.92 7.92 7.66 7.66 7.64 7.64 7.61 7.61 7.60 7.59 7.58 7.58 7.56 7.41 7.39 7.39 7.37 7.33 7.32 7.30 7.24 7.23 7.23 7.20 7.20 7.19 7.15 7.14 7.14 6.93 6.88
5.32 CD2Cl2
F
O
S
O
1.80
1.27
3.19
1.93
1.37
1.24
1.29
8.3 8.2 8.1 8.0 7.9 7.8 7.7 7.6 7.5 7.4 7.3 7.2 7.1 7.0 6.9 6.8 6.7 6.6 6.5 6.4 6.3 6.2 6.1 6.0 5.9 5.8 5.7 5.6 5.5 5.4 5.3 5.2 5.1 5.0
ppm

4j

^{13}C

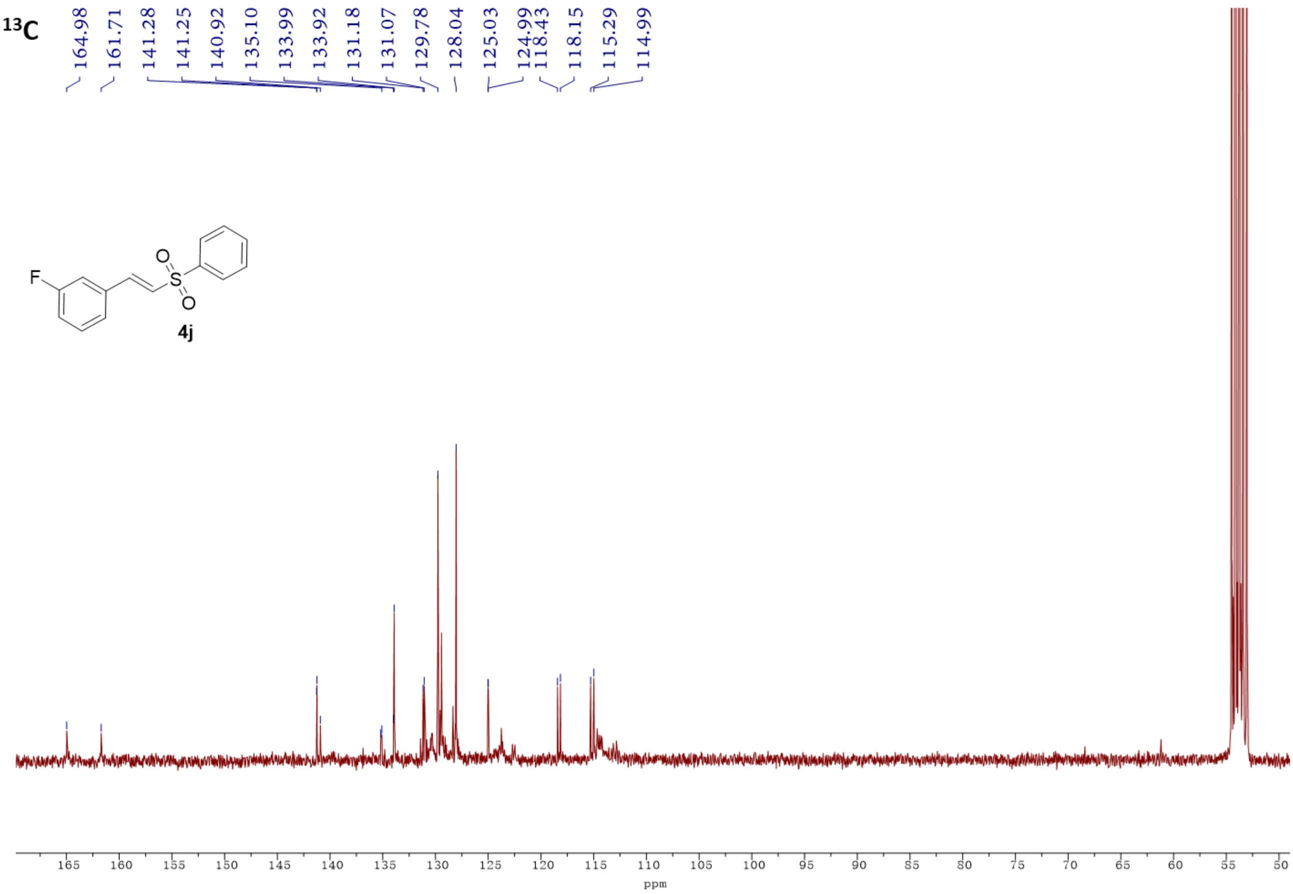
164.98
161.71
141.28
141.25
140.92
135.10
133.99
133.92
131.18
131.07
129.78
128.04
125.03
124.99
118.43
118.15
115.29
114.99
F
O
S
O
4j
ppm

^{19}F

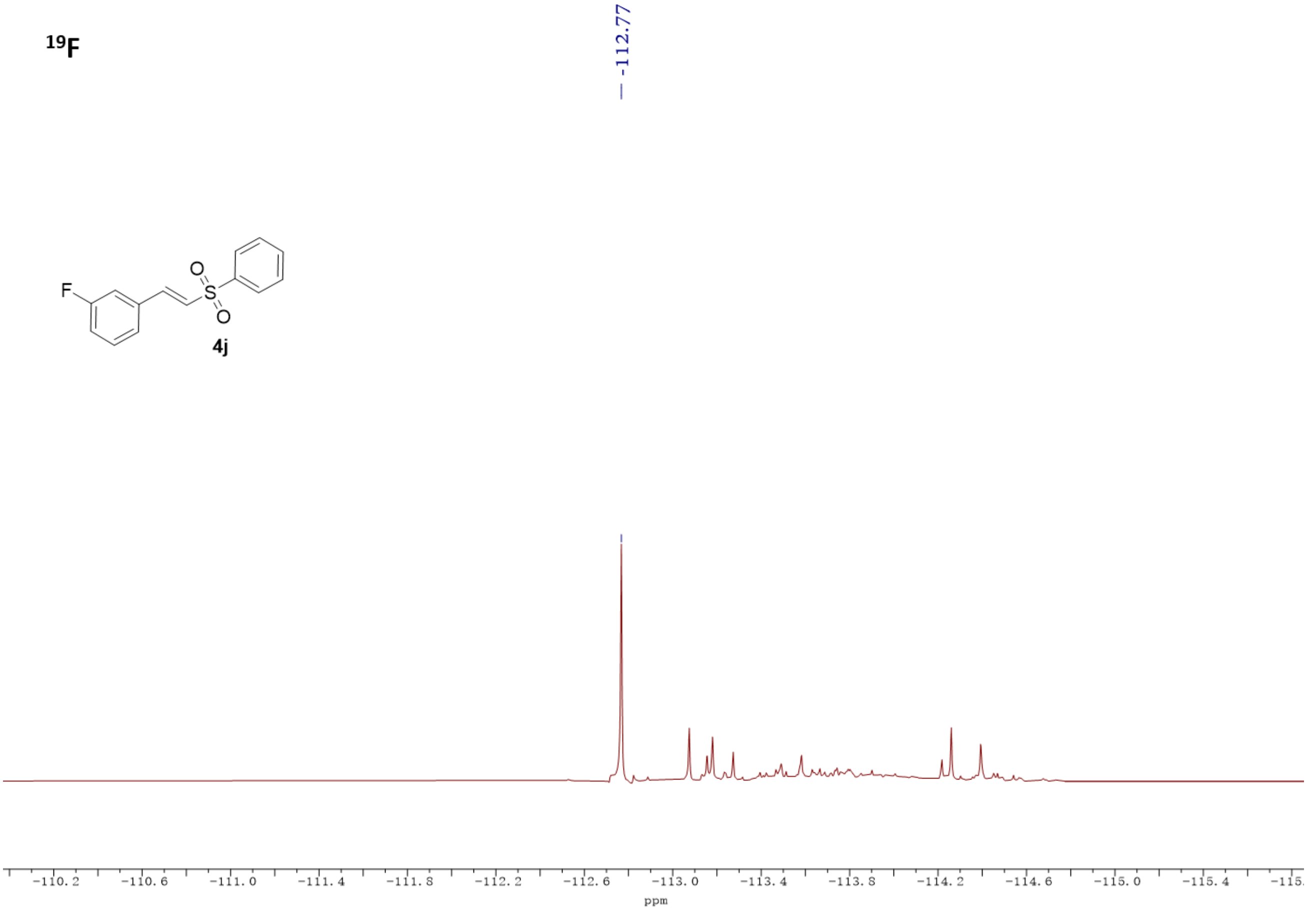
-112.77
F
O
S
O
4j
ppm

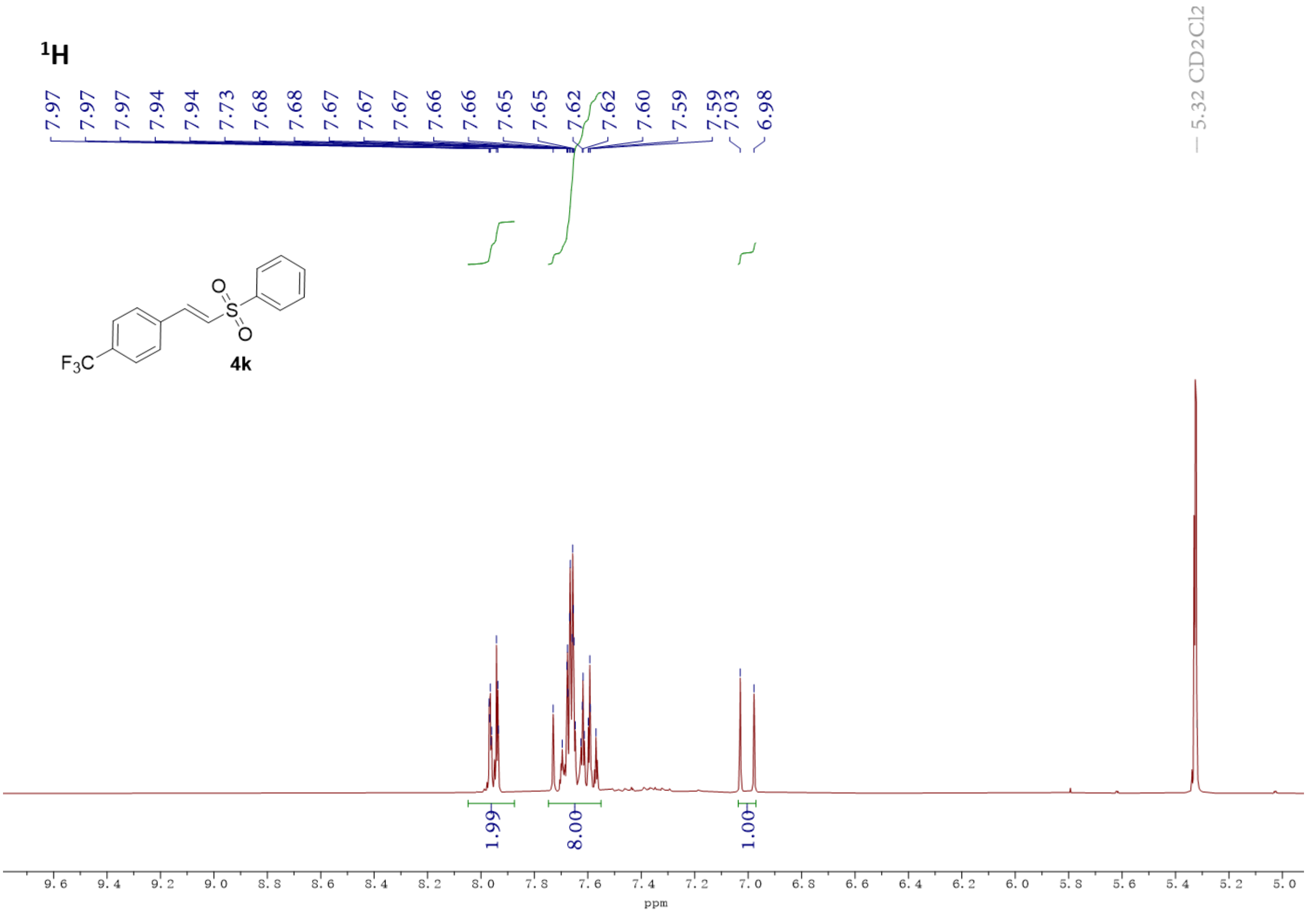
1H
7.97
7.97
7.97
7.94
7.94
7.73
7.68
7.68
7.67
7.67
7.67
7.66
7.66
7.65
7.65
7.62
7.62
7.60
7.59
7.59
7.03
6.98
5.32 CD2Cl2
F3C
4k
1.99
8.00
1.00
ppm

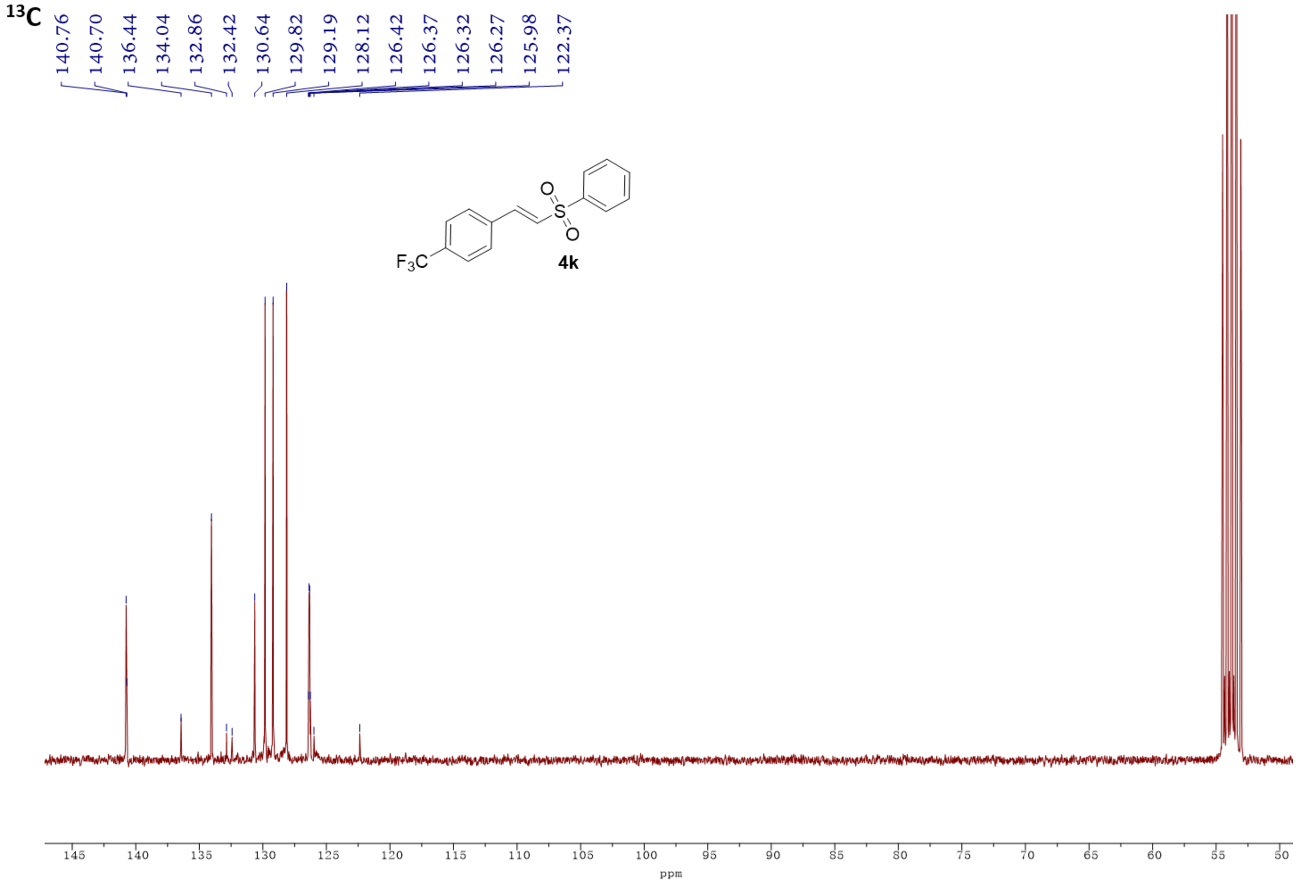
13C
140.76
140.70
136.44
134.04
132.86
132.42
130.64
129.82
129.19
128.12
126.42
126.37
126.32
126.27
125.98
122.37
F3C
4k
ppm

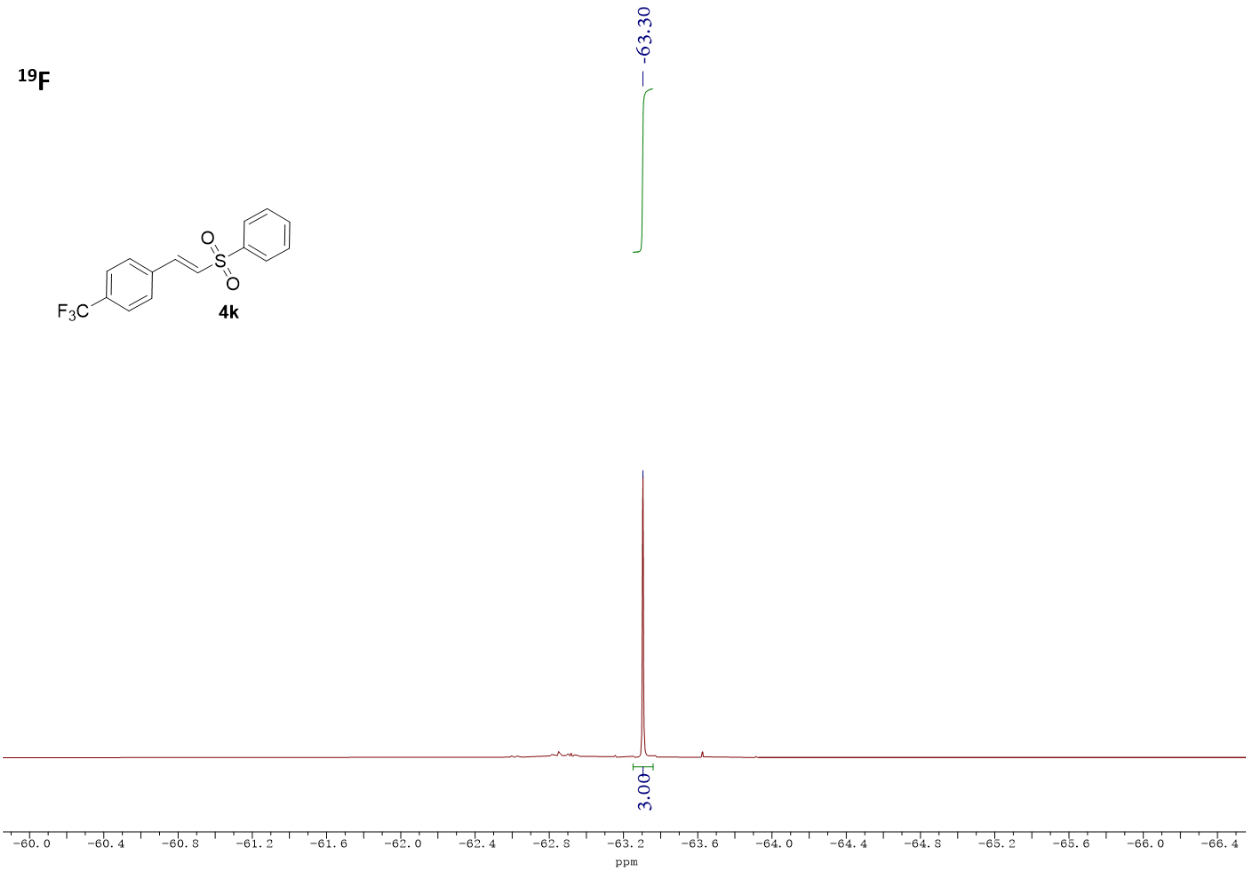

19F
-63.30
F3C
4k
3.00
ppm

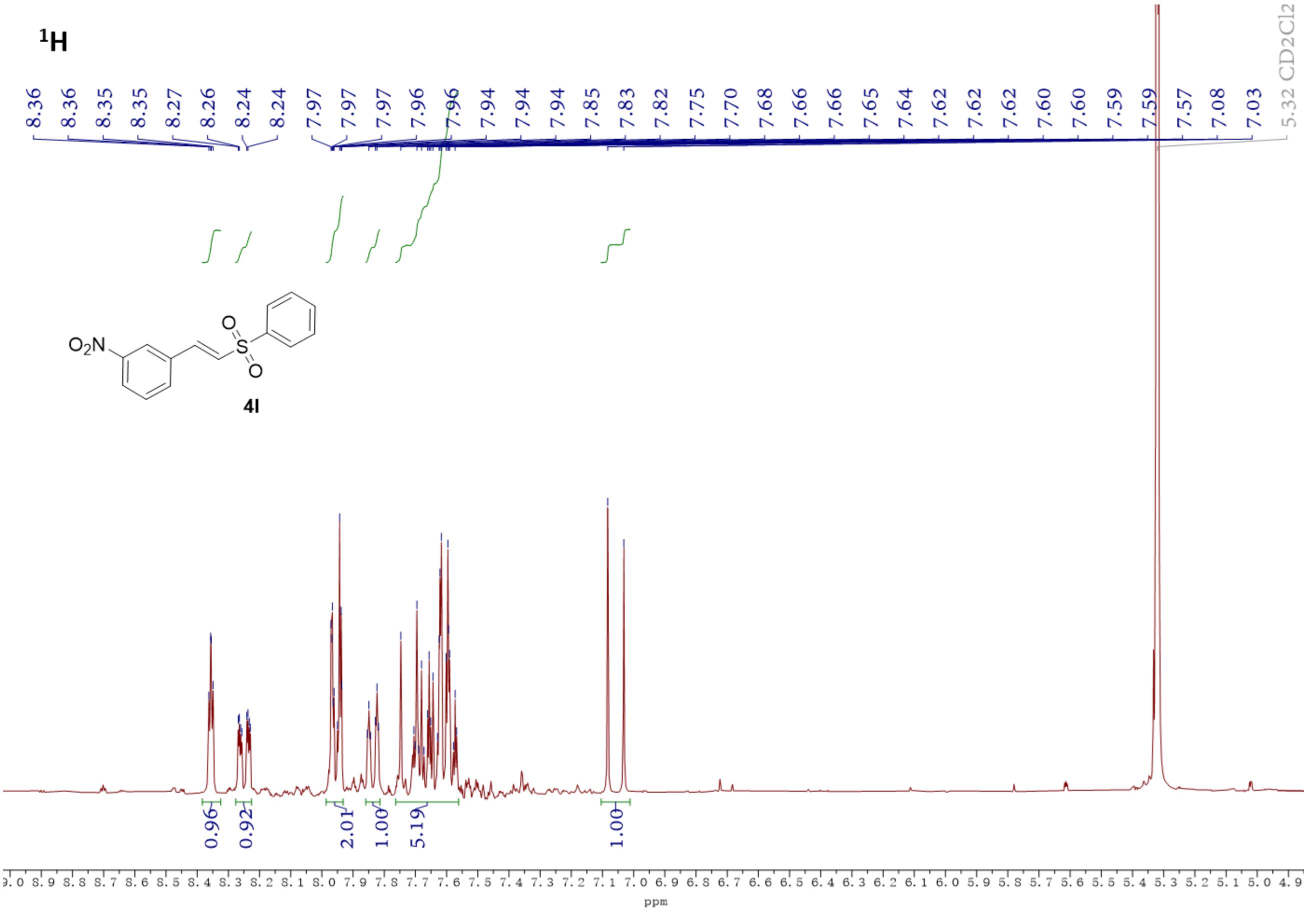

1H
8.36 8.36 8.35 8.35 8.27 8.26 8.24 8.24 7.97 7.97 7.97 7.96 7.96 7.94 7.94 7.94 7.85 7.83 7.82 7.75 7.70 7.68 7.66 7.66 7.65 7.64 7.62 7.62 7.62 7.60 7.60 7.59 7.59 7.57 7.08 7.03
5.32 CD2Cl2
O2N
4l
0.96 0.92 2.01 1.00 5.19 1.00
ppm

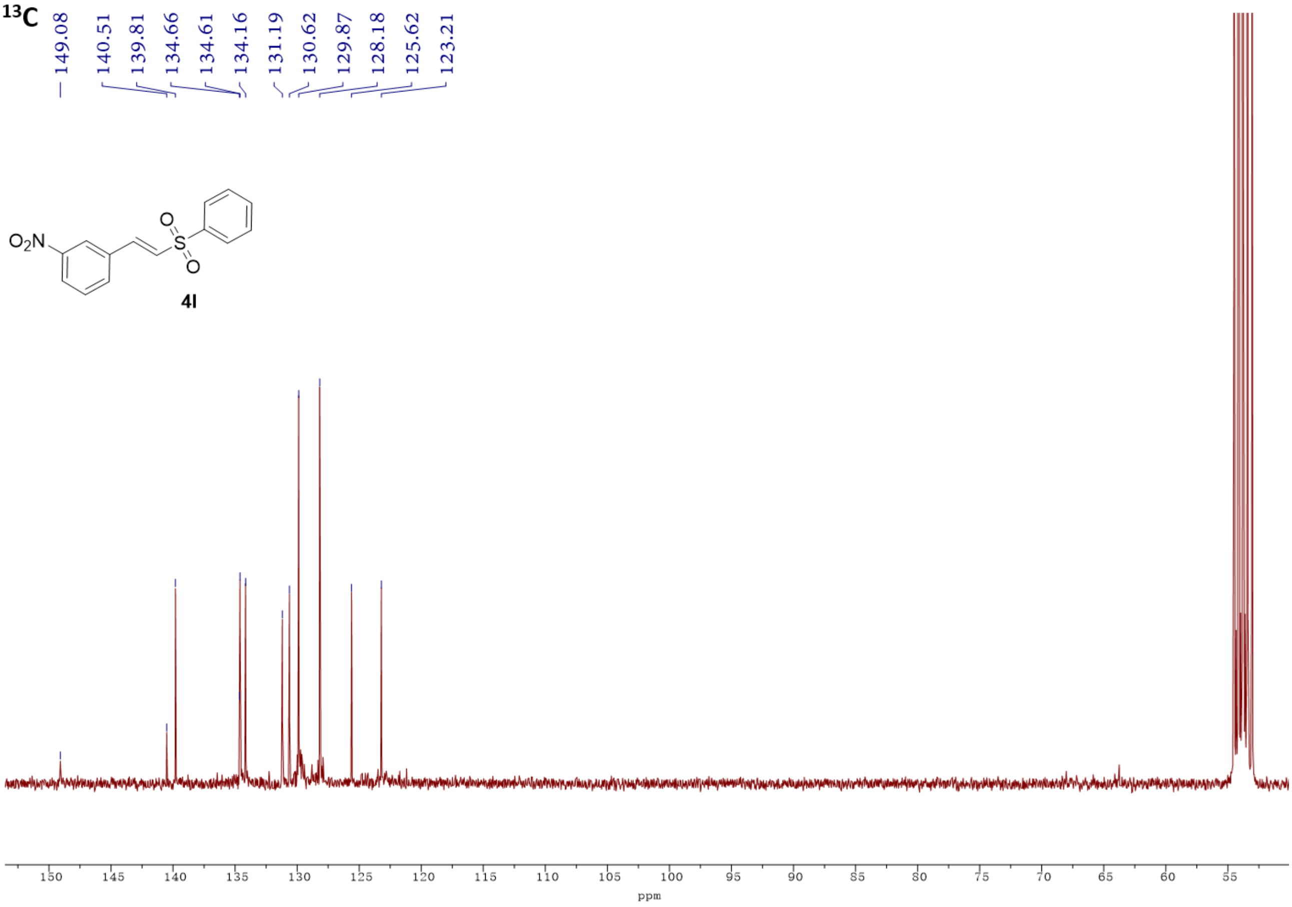
13C
149.08
140.51
139.81
134.66
134.61
134.16
131.19
130.62
129.87
128.18
125.62
123.21
O2N
O
S
O
4l
150 145 140 135 130 125 120 115 110 105 100 95 90 85 80 75 70 65 60 55
ppm

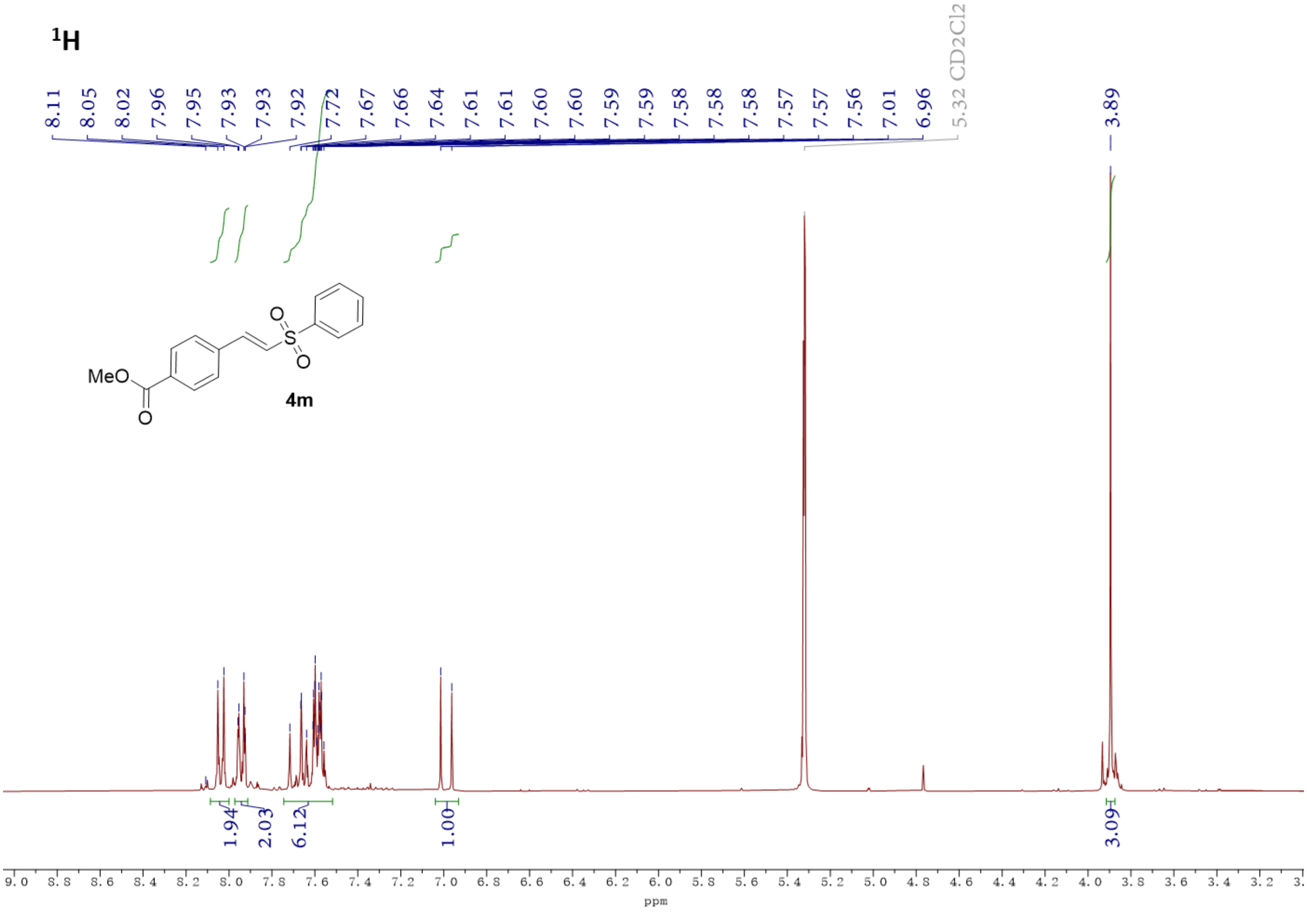
1H
8.11
8.05
8.02
7.96
7.95
7.93
7.93
7.92
7.72
7.67
7.66
7.64
7.61
7.61
7.60
7.60
7.59
7.59
7.58
7.58
7.58
7.57
7.57
7.56
7.01
6.96
5.32 CD2Cl2
3.89
MeO
O
O
S
O
4m
1.94
2.03
6.12
1.00
3.09
9.0 8.8 8.6 8.4 8.2 8.0 7.8 7.6 7.4 7.2 7.0 6.8 6.6 6.4 6.2 6.0 5.8 5.6 5.4 5.2 5.0 4.8 4.6 4.4 4.2 4.0 3.8 3.6 3.4 3.2
ppm

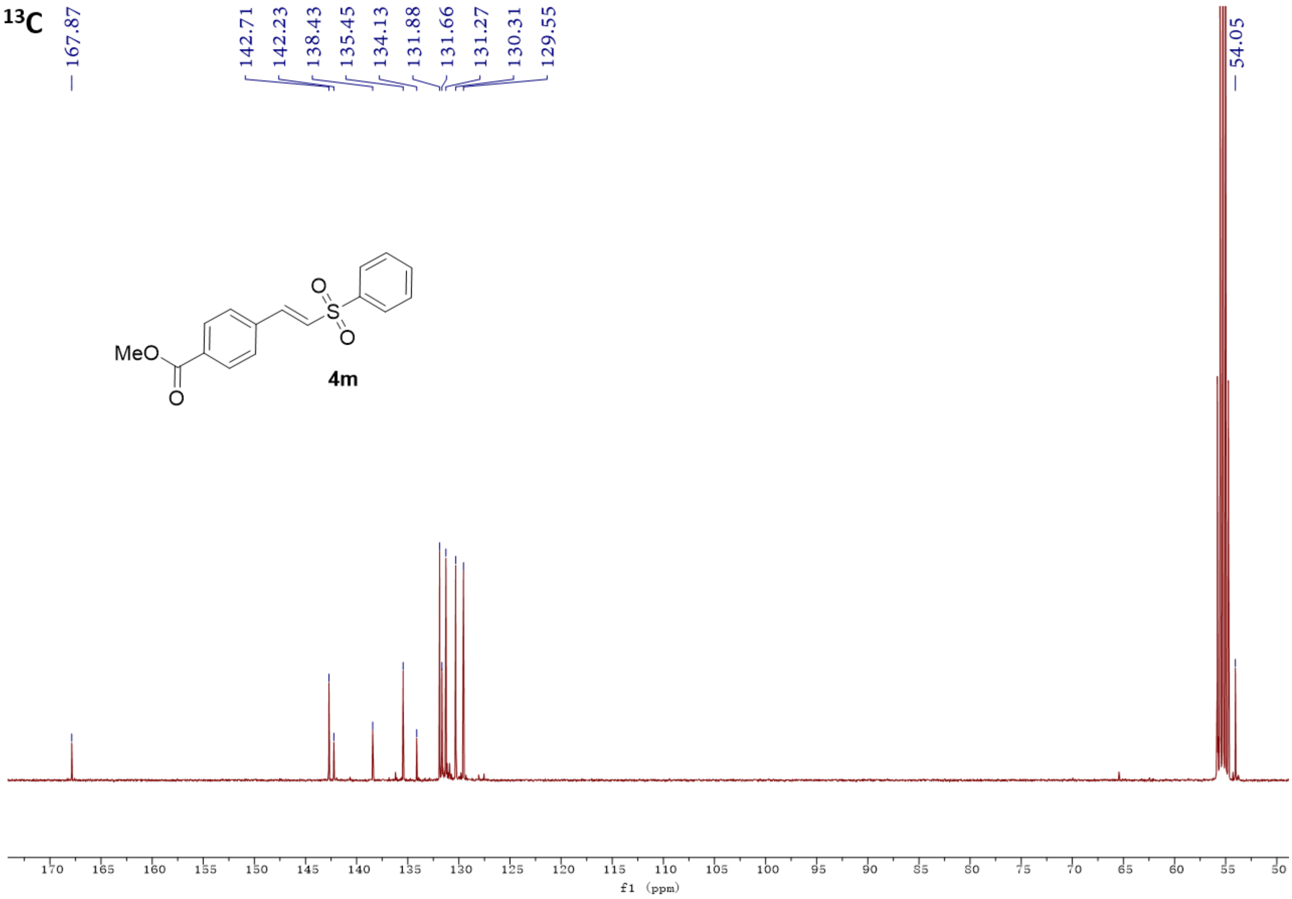
13C
167.87
142.71
142.23
138.43
135.45
134.13
131.88
131.66
131.27
130.31
129.55
54.05
MeO
O
S
4m
170
165
160
155
150
145
140
135
130
125
120
115
110
105
100
95
90
85
80
75
70
65
60
55
50
f1 (ppm)
